# The efficacy of XEN gel stent implantation in glaucoma: a systematic review and meta-analysis

**DOI:** 10.1186/s12886-022-02502-y

**Published:** 2022-07-15

**Authors:** Xiang Yang, Yang Zhao, Yu Zhong, Xuanchu Duan

**Affiliations:** 1 Changsha Aier Eye Hospital, Changsha, Hunan China; 2 Aier School of Ophthalmology, Central South University, Changsha, Hunan China

**Keywords:** Minimally invasive glaucoma surgery, XEN, Meta-analysis

## Abstract

**Background:**

Xen is a device for minimally invasive glaucoma surgery, and is used to treat POAG, pseudoexfoliative or pigmentary glaucoma, as well as refractory glaucoma. The efficacy of XEN in treating glaucoma remains to be confirmed and clarified. Hence, we conducted a systematic review and meta-analysis to examine the efficacy and associated complication of XEN implantations.

**Methods:**

We conducted a literature search in PubMed, EMBASE, the Cochrane Library of Systematic Reviews, Web of Science, China National Knowledge Infrastructure, WanFang and SinoMed databases to identify studies, published before May 15, 2021, which evaluated XEN in glaucoma, and parameters for measurements included intra-ocular pressure (IOP), number of anti-glaucoma medications (NOAM), and bleb needling rate. We compared the measurements of XEN-only procedure between phaco-XEN and trabeculectomy, and we also did sub-analysis based on time points, glaucoma types, ethnics, etc. Sensitivity analyses and publication bias were conducted for evaluating bias.This study followed the Preferred Reporting Items for Systematic Reviews and Meta-analyses (PRISMA 2020) reporting guideline.

**Results:**

We identified 78 eligible studies, analysis revealed obvious IOP reduction after XEN stent implantation (SMD: 1.69, 95% CI 1.52 to 1.86, *p* value < 0.001) and NOAM reduction (SMD: 2.11, 95% CI 1.84 to 2.38, *p* value < 0.001). Sub-analysis showed no significant difference with respect to time points, ethnicities, and economic status. No significant difference was found between XEN treatment effect on POAG and PEXG eyes and between pseudo-phakic and phakic eyes. Also no significant difference was found between XEN and phaco-XEN surgery in terms of IOP after surgery (SMD: -0.01, 95% CI -0.09 to 0.08, *p* value 0.894). However, NOAM (after publication bias correction) and bleb needling rate (RR: 1.45, 95% CI 1.06to 1.99, *p* value 0.019) were lower in phaco-XEN group compared to XEN only group. Compared to trabeculectomy, XEN implantation had similar after-surgery IOP, however bleb needling rate (RR: 2.42, 95% CI 1.33 to 4.43, *p* value 0.004) was higher.

**Conclusion:**

Our results confirmed that XEN is effective in lowering both IOP and NOAM till 48 months after surgery. It is noteworthy that XEN implantation leads to higher needling rate, compared to phaco-XEN or trabeculectomy. Further research, studying complications of XEN on non-European ethnicities, especially on Asian, are in urgent need before XEN is widely applied.

**Supplementary Information:**

The online version contains supplementary material available at 10.1186/s12886-022-02502-y.

## Introduction

Glaucoma is known as the global leading cause of irreversible blindness [1], and statistics shows that people of African ancestry are more sensitive to primary open angle glaucoma (POAG) than people of European ancestry [2].Intra-ocular pressure(IOP) lowering-laucoma treatments include anti-glaucoma medications, laser, surgery, and the combinations. Surgery is required when medications fail to control IOP or visual loss has reached a serious threshold.

Trabeculectomy and drainage device implantation are two methods widely adopted by clinicians. Trabeculectomy has become a standard surgical for glaucoma [3], it bypasses trabecular mesh and builds a drainage to help aqueous humor flow from anterior chamber to subconjunctival space. However, it can lead to high rates of complications including hypotony, anterior chamber hyphemia [4], etc. Minimally invasive glaucoma surgery (MIGS) has become surgical trend in recent years. As a type of MIGS, XEN Gel Stent (Allergan INC, Dublin, Ireland) implantations mimic the subconjunctival drainage of trabeculectomy, and are applied in real world since FDA approval in 2016. XEN implant is a 6-mm tube, made of porcine-gelatin cross-linked with glutaraldehyde, and has advantages of non-degrading and no tissue reaction [5] XEN45, the type of XEN that is now being merchandised, is designed to prevent hypotony and to maintain IOP around 6-8 mmHg with inner diameter of 45 μm [6]. XEN45 and XEN63, which is the new type of XEN, also have the indication of treating refractory POAG, and other types of OAG including pseudo-exfoliative glaucoma (PEXG). However, there are, currently, different opinions on the efficacy of XEN compared to traditional surgery in glaucoma according to previous studies [7–9], and its complications are also remain to be further investigated.

XEN is much easier to operate than trabeculectomy, thus it may help ophthalmologists, not specialists to treat glaucoma. Still, more evidence is required on the efficacy and complications of XEN before the device is widely applied. In this review, we did the most comprehensive meta-analysis on qualified clinical trials on this theme. With the data extracted, we did analysis to compare IOP-lowering and medication-lowering efficacy of XEN-only to XEN combined with phacoemulsification (phaco-XEN) and trabeculectomy surgeries respectively. Needling rate in different surgeries was also compared to study complications. Sub-analyses were carried out according to different study design, type of glaucoma, ethnicities, populations, economic status, and time points of follow-up to reduce confounding from those factors.

## Material and method

This review is written according to Preferred Reporting Items for Systematic Reviews statement for reporting systematic reviews and meta-analyses [10] (Additional file 1: Appendix 1).

### Search strategy

Electronic databases, including PubMed, EMBASE, the Cochrane Library of Systematic Reviews, Web of Science, China National Knowledge Infrastructure, WanFang and SinoMed databases were searched up to May 2021 for all clinical studies assessing XEN implant in glaucoma. The search strategy included the Medical Subject Headings terms and/or text words. The following combined search term was used: (XEN implant, XEN Gel Stent, gelatin stent) and (Glaucoma) (for the full search strategy, see the Additional file 2: Appendix 2 in the Supplement). The studies were restricted to human, but not restricted by date, language, or publication status.

### Study selection

Studies were selected by two independent reviewers (Xiang Yang andYang Zhao using following criteria:

1. patients were clearly diagnosed with glaucoma (no matter for POAG or PEXG, etc.);
2. the study had a control design;
3. XEN stent (XEN-45 or XEN-63) was used;
4. sufficient information to calculate the effect size was available;
5. the manuscript was published in a peer-reviewed journal as a full paper.

And criteria for excluding studies were:

1. Animal studies;
2. No original studies (case report, letter and response, review and meta-analysis or meeting abstract)

In the first stage, the titles and abstracts of all retrieved articles were screened. Disagreements were referred to a third reviewer (Yu Zhong) to achieve a resolution. In the second stage, full texts of the potentially relevant studies were retrieved and reviewed using the same methods as in first stage.

### Data extraction and quality assessment

The following information was independently extracted from the included studies by two investigators (Xiang Yang andYang Zhao) and jointly verified for accuracy: author, year of publication, country of study, eyes included, female/male ratio, age, surgical implantation, follow-up period, etc. We contacted authors when there was unclear information. JADAD Scale [11] (for Randomized Controlled Trials) or Newcastle–Ottawa Scale [12] (for non-randomized Studies) were used for evidence quality assessment.

### Outcome measures

The final included outcomes were: IOP before and after surgery; number of antiglaucoma medications (NOAM); bleb needling rate.

### Statistical analysis

The pooled relative risk (RR) or standardized mean difference (SMD) in the meta-analysis were calculated by weighting individual risk ratio (RR)/SMD by the inverse of their variance. The RRs as well as 95% CIs were calculated using the random-effects model as it assumes that true effect might vary from study to study and thus, estimates the mean of a distribution of true effects, assigning a more balanced weight to each study. All tests were two-tailed with a *p* value < 0.05 considered statistically significant. Analysis using the fixed-effects model was carried out in the absence of heterogeneity. The Cochran’s Q test was used to test for heterogeneity (*p* value < 0.10 is indicative of heterogeneity). Given that the power of this statistical test is low when a meta-analysis includes a small number of studies, the Higgins test (*I*^*2*^) was also used, that describes the percentage of total variation across studies due to heterogeneity rather than chance (low heterogeneity: < 25%, moderate heterogeneity: 25–75% and high heterogeneity: > 75%) [13]. Leave-one-out sensitivity as well as stratified analyses were conducted to assess statistical robustness and to detect the possible causes of heterogeneity between studies. The Begg rank correlation [14] and *Egger* regression asymmetry test [15] were used to examine publication bias (*P* < 0.05 was considered statistically significant). If publication bias was confirmed, a trim-and-fill method developed by Duval and Tweedie was implemented to adjust the bias. Then, we replicated the funnel plot with their ‘‘missing’’ counterparts around the adjusted summary estimate. All those were conducted with the software Stata 15.0.

## Results

### Literature search

The search strategy for this meta-analysis yielded 725 publications, and 429 studies were excluded because of duplication. After reading the titles and abstracts, 57 studies were excluded. 239 possible full-text studies were carefully reviewed (Animal study [*n* = 4]; Case report [60]; Letter and Response [*n*

**Fig. 1:**
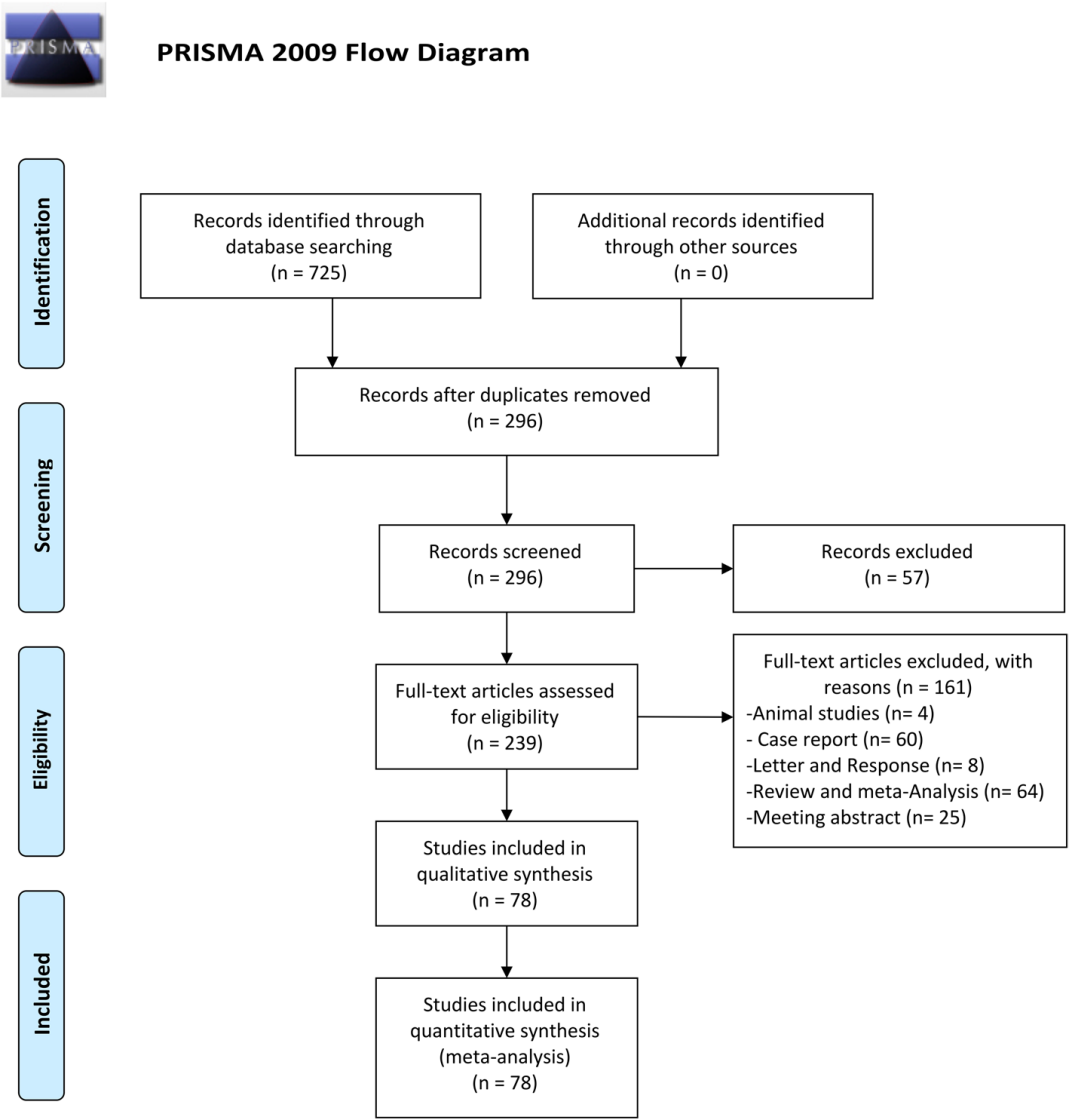
PRISMA 2009 flow diagram. The search strategy for this meta-analysis yielded 725 publications, and 429 studies were excluded because of duplication. After reading the titles and abstracts, 57 studies were excluded. Two hundred and thirty-nine possible full-text studies were carefully reviewed (Animal study [*n* = 4]; Case report [60]; Letter and Response [*n* = 8]; Review and meta-Analysis [*n* = 64]). Finally, 78 trials were included for quantitative analysis

 = 8]; Review and meta-Analysis [*n* = 64]; Meeting abstract [*n* = 25]). Finally, 78 trials were included for quantitative analysis [5, 16–92] (Fig. 1). The characteristics of included lectures are summarized in Table 1.

**Table 1:** Main characteristics of the included studies in the meta-analysis

First Author	Publish Year	Country	Study design	Eyes included	Male/Female	Age (Mean ± SD)	Surgical Implantation	Follow-up	JADA/NOS score
Sheybani, A [5]	2015	US	Prospective	37	14/23	69.6 ± 7.7	phaco-XEN	12 m	1
Pérez-Torregrosa, V. T [70]	2016	Spain	Prospective	30	5/13	76 ± 5.85	phaco-XEN	12 m	1
Sheybani, A [81]	2016	US	Prospective	49	20/29	64.3	XEN	12 m	1
Fea, A. M [29]	2017	Italy	Prospective	12	5/6	71.3 ± 10	XEN	12 m	1
Galal, A [33]	2017	Germany	Prospective	13	6/4	73.1 ± 10	phaco-XEN	12 m	1
Grover, D [37]	2017	US	Prospective	65	30/35	70 ± 12.3	XEN	12 m	0
Hengerer, F. H [41]	2017	Germany	Retrospective	242	100/142	67.6 ± 13.6	XEN; phaco-XEN	12 m	5
Ilveskoski, L [47]	2017	Finland	Retrospective	10	4/6	77.4 ± 5.7	XEN	6 m	/
Olate-Pérez, Á. [66]	2017	Spain	Prospective	30	5/13	76 ± 5.85	phaco-XEN	12 m	1
Ozal, S.A [69]	2017	Turkey	Retrospective	15	10/5	63.6 ± 13.3	XEN; phaco-XEN	12 m	/
Schlenker, M. B [79]	2017	Canada	Retrospective	354	176/178	66.4	XEN; TB	36 m	7
Arnljots, TS [17]	2018	Sweden	Retrospective	19	7/12	74.2 ± 8.4	XEN; phaco-XEN	12 m	/
De Gregorio, A [26]	2018	Italy	Prospective	41	13/20	74 ± 7.1	phaco-XEN	12 m	0
Hengerer, F. H [40]	2018	Germany	Retrospective	110	46/64	69.6 ± 13.7	XEN	12 m	5
Hohberger, B [42]	2018	Germany	Retrospective	111	64/47	68 ± 14	XEN	6 m	
Karimi, A [49]	2018	UK	Retrospective	17	9/8	76.1	XEN	12 m	
Karimi, A [50]	2018	UK	Retrospective	259	144/115	74.8	XEN; phaco-XEN	18 m	
Mansouri, K [59]	2018	Switzerland	Prospective	110	24/61	74.8 ± 9.4	XEN	12 m	1
Mansouri, K [60]	2018	Switzerland	Prospective	149	32/81	74.4 ± 9.4	XEN; phaco-XEN	12 m	1
Sng, C. C [83]	2018	UK	Prospective	24	9/15	45.3 ± 18.1	XEN	12 m	0
Tan, S. Z [85]	2018	UK	Retrospective	43	18/21	70.1 ± 13.8	XEN	12 m	
Widder, R. A [91]	2018	Germany	Retrospective	261	92/141	73 ± 11	XEN; phaco-XEN	18 m	
Arad, T [16]	2019	Germany	Retrospective	10	4/6	6.4 ± 4.7	XEN	24 m	
Gillmann, K [34]	2019	Switzerland	Prospective	110	24/61	74.8 ± 9.4	XEN; phaco-XEN	24 m	1
Heidinger, A [38]	2019	Austria	Retrospective	199	84/115	74.8 ± 10.5	XEN	18 m	
Hengerer, F. H [39]	2019	Germany	Retrospective	148	89/59	68.4 ± 13.9	XEN	12 m	
Ibáñez-Muñoz, A [45]	2019	Spain	Retrospective	21	13/7	80.9 ± 8.1	XEN; phaco-XEN	12 m	
Kalina, AG [48]	2019	USA	Prospective	47	14/28	78.15 ± 8.55	XEN; phaco-XEN	12 m	1
Laroche, D [52]	2019	US	Retrospective	12	-	-	XEN	12 m	
Lenzhofer, M [54]	2019	AustriaTalbel	Prospective	64	35/29	-	XEN	48 m	1
Lenzhofer, M [55]	2019	Austria	Prospective	137	67/70	75.2 ± 7.0	XEN; phaco-XEN	24 m	1
Lenzhofer, M [56]	2019	Austria	Prospective	66	28/38	72.2 ± 12.5	XEN; phaco-XEN	12 m	0
Mansouri, K [58]	2019	Switzerland	Prospective	149	32/81	74.4 ± 9.4	XEN; phaco-XEN	24 m	1
Marcos Parra, M.T [61]	2019	Spain	Retrospective	121	59/62	71.2 ± 11.7	XEN; phaco-XEN; TB	12 m	
Marques, RE [62]	2019	Portugal	Retrospective	60	26/34	73	XEN; phaco-XEN	6 m	
Midha, N [64]	2019	Switzerland	Prospective	149	63/70	74.4 ± 9.6	XEN; phaco-XEN	24 m	1
Qureshi, A [72]	2019	UK	Retrospective	37	-	45.97 ± 15.24	XEN	12 m	
Reitsamer, H [75]	2019	Austria	Prospective	161	90/95	71.8 ± 10.5	XEN; phaco-XEN	24 m	1
Smith, M [82]	2019	UK	Retrospective	68	35/33	76 ± 10	XEN	12 m	
Teus, M. A [87]	2019	Spain	Retrospective	48	27/21	72.7 ± 12.51	XEN	48 m	
Barão, R.C [18]	2020	Portugal	Retrospective	42	12/30	71.7 ± 12	XEN; phaco-XEN	18 m	
Başer, E. F [19]	2020	Turkey	Retrospective	29	17/12	67.5 ± 10.3	XEN	24 m	
Bravetti, G.E [20]	2020	Switzerland	Retrospective	60	32/28	64.7 ± 23.1	XEN	12 m	
Buffault, J [21]	2020	France	Retrospective	107	58/49	68.3 ± 10.8	XEN; phaco-XEN	6 m	
Busch, T [22]	2020	Sweden	Retrospective	113	53/50	70.8 ± 11.8	XEN	12 m	
Cutolo, CA [24]	2020	Italy	Prospective	123	58/65	74.5 (67.1–81.3)	XEN	12 m	1
Dar, N [25]	2020	Israel	Retrospective	46	22/24	74 ± 9.4	XEN	6 m	
Do, A [27]	2020	US	Retrospective	137	76/61	72 ± 13.2	XEN	12 m	
Fea, A. M [28]	2020	Italy	Prospective	298	149/149	70.3 ± 11.8	XEN; phaco-XEN	12 m	1
Fernández-García, A [30]	2020	Spain	Retrospective	40	17/23	77.31 ± 6.33	XEN	36 m	
Fernández-García, A [31]	2020	Spain	Retrospective	93	22/41	74 ± 8	XEN	36 m	
Gabbay, I. E [32]	2020	UK	Retrospective	151	82/69	74.3 ± 11.0	XEN; phaco-XEN	24 m	
Gillmann, K [35]	2020	Switzerland	Prospective	92	23/45	76.3 ± 9.1	XEN; phaco-XEN	36 m	1
Gillmann, K [36]	2020	Switzerland	Prospective	37	10/27	77.7 ± 9.1	XEN	24 m	1
Hong, K [43]	2020	US	Prospective	28	11/17	66.6 ± 11	XEN	12 m	0
Hu, J. Y [44]	2020	Singapore	Retrospective	63	50/13	71.9 ± 7.1	XEN; phaco-XEN	6 m	
Ibáñez-Muñoz, A [46]	2020	Spain	Retrospective	73	39/34	79.7 ± 8.2	XEN; phaco-XEN	12 m	
Laborda-Guirao, T [51]	2020	Spain	Retrospective	80	42/38	74.0 + 10.4	XEN; phaco-XEN	12 m	
Lavin-Dapena, C [53]	2020	Spain	Prospective	11	2/9	78.8	XEN; phaco-XEN	18 m	0
Linton, E [57]	2020	UK	Retrospective	151	38/113	71 ± 12.6	XEN	12 m	
Midha, N [63]	2020	Switzerland	Prospective	51	15/36	74.4 ± 9.4	XEN; phaco-XEN	24 m	0
Olgun, A [67]	2020	Turkey	Retrospective	221	42/72	65.8 ± 10.6	XEN; phaco-XEN; TB	24 m	
Olgun, A [68]	2020	Turkey	Retrospective	80	29/35	61.1 ± 12.1	XEN; TB	3 m	
Post, M [71]	2020	Poland	Prospective	20	6/11	69.85 ± 4.69	XEN	12 m	1
Rather, P.A. [73]	2020	US	Retrospective	92	31/35	75.3	XEN; phaco-XEN	12 m	
Rauchegger, T [74]	2020	Austria	Retrospective	79	49/30	-	XEN; phaco-XEN	24 m	
Schargus, M [77]	2020	Germany	Retrospective	113	73/80	70.2 ± 10.8	XEN	12 m	
Scheres, M. J [78]	2020	Netherlands	Retrospective	82	41/41	69 ± 8	XEN	24 m	
Sharpe, R [80]	2020	US	Retrospective	179	88/91	74.5 ± 7.6	XEN; TB	6 m	
Teixeira, F.J [86]	2020	Portugal	Prospective	12	6/6	59 ± 19	XEN	12 m	1
Theillac, V [88]	2020	France	Retrospective	105	47/58	72.1 ± 8.7	XEN	6 m	
Wałek, E [90]	2020	Poland	Prospective	39	19/20	67	XEN; phaco-XEN	24 m	1
Widder, R. A [92]	2020	Germany	Retrospective	90	-	72 ± 13	XEN	48 m	
Chao, Y.J [23]	2021	China	Retrospective	37	24/14	53.4 ± 13.6	XEN	12 m	
Oddone, F [65]	2021	Italy	Prospective	108	84/84	69.1 ± 12.9	XEN; phaco-XEN	6 m	0
Reitsamer, H [76]	2021	Austria	Retrospective	212	83/94	76 ± 7.1	XEN; phaco-XEN	36 m	
Tan, N.E [84]	2021	US	Retrospective	50	-	71.0 ± 13.4	XEN	12 m	
Urcola, A [89]	2021	Spain	Retrospective	20	3/7	76.1 ± 12	XEN; phaco-XEN	12 m	

### Efficacy of XEN standalone surgery for the management of glaucoma

6554 eyes from 65 studies and 4385 eyes from 42 studies were included for IOP and NOAM analysis before and after XEN standalone procedure. There were no significant differences in IOP or NOAM between prospective and retrospective study (SFig1 and SFig2). Therefore, we combined them in the further analyses.

The total study sample included 3432 eyes before surgery and 3122 eyes after surgery. Overall analysis showed IOP had an obvious improvement after XEN stent implantation (SMD: 1.69, 95% CI 1.52 to 1.86, *p* value < 0.001) (sFig3). Fewer NOAM was also achieved in glaucoma patients after XEN standalone procedure (SMD: 2.11, 95% CI 1.84 to 2.38, *p* value < 0.001) (sFig4). Based on the follow-up duration, studies were divided into six categories: 6 m, 12 m, 18 m, 24 m, 36 m and 48 m. Considering IOP, no big difference was found at different time point (sFig5). As for NOAM, the difference became less and less with time, although significance was still not reached (sFig6). 6 studies addressing the IOP-lowering effect of XEN in Asian populations (168 eyes in before-surgery group and 160 eyes in after-surgery group), 8 studies addressing the treatment effect of XEN in North American populations (351 eyes in before surgery group and 284eyes in after surgery group) and 45 studies addressing this association in European populations (2913 eyes in before surgery group and 2678 eyes in after surgery group), were included in the stratified analyses by ethnicity (SFig7). Subgroup was further done by developed vs. developing country (SFig8). No statistical difference was found in different gene background and medical care, the patients could get. NOAM reduction had no difference indeveloped *vs*developing country subgroup analysis (SFig9) orethnicity subgroup analysis (SFig10). Heterogeneity was high in most of the stratified analyses.

Given that differences in the pseudo-exfoliation glaucoma (PEXG) and primary open angle glaucoma (POAG) could potentially bias the current meta-analysis, analyses by different glaucoma were also conducted. Three studies with 237 POAG eyes and 118 PEXG eyes were included. Interestingly, no different treatment effect was found in these analyses on IOP and Medication (SFig11-14). Furthermore, analysis was conducted in patients with or without prior interventional therapies and patients with pseudophakic and phakic eyes. IOP before and after XEN surgery, medication before and after procedure and bleb needling rate shown no difference in pseudophakic and phakic eyes (SFig15-19).

### Efficacy and safety of XEN combined with cataract surgery for glaucoma patients

**Fig. 2:**
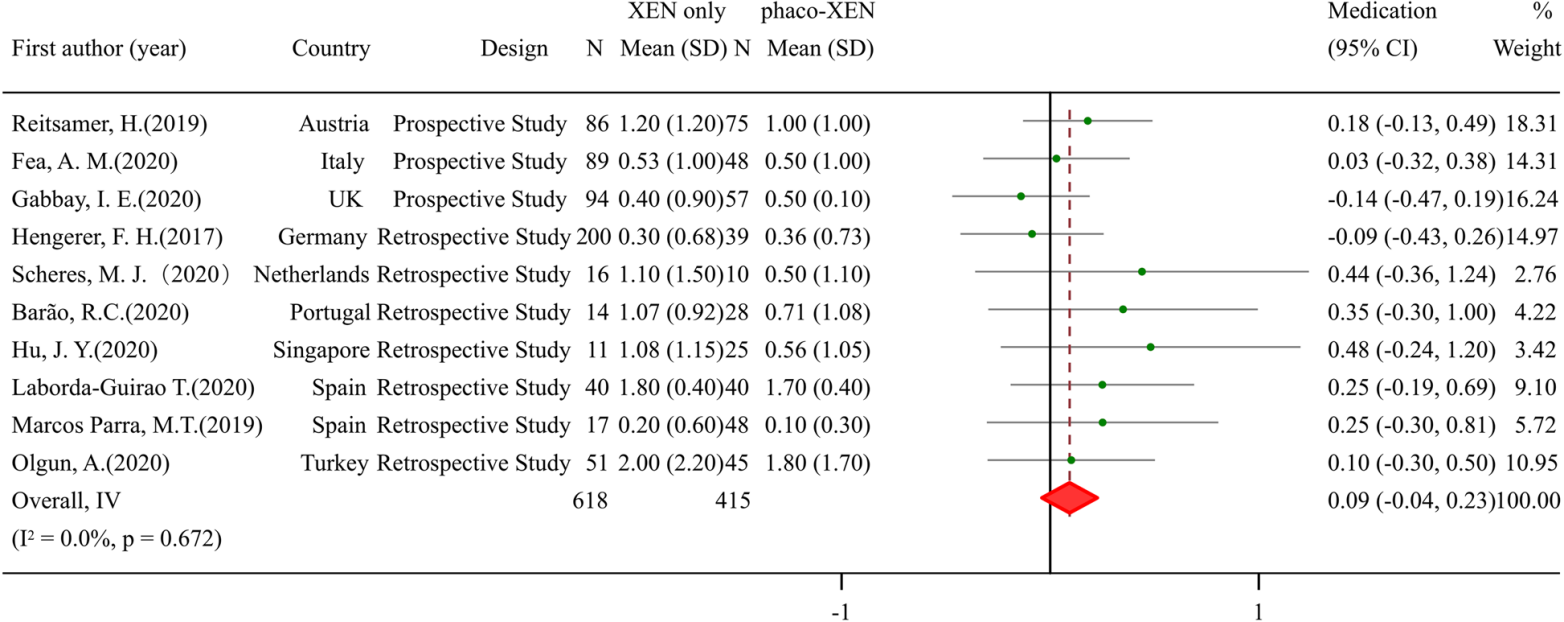
Meta-analysis of XEN-only surgery compared with phaco-XEN for NOAM after surgery. The total study sample included 618 eyes undergoing XEN only and 415 eyes undergoing phaco-XEN. Overall analysis of NOAM after surgery (SMD: 0.09, 95% CI -0.04 to 0.23, *p* value 0.170)had no difference between two groups

**Fig. 3:**
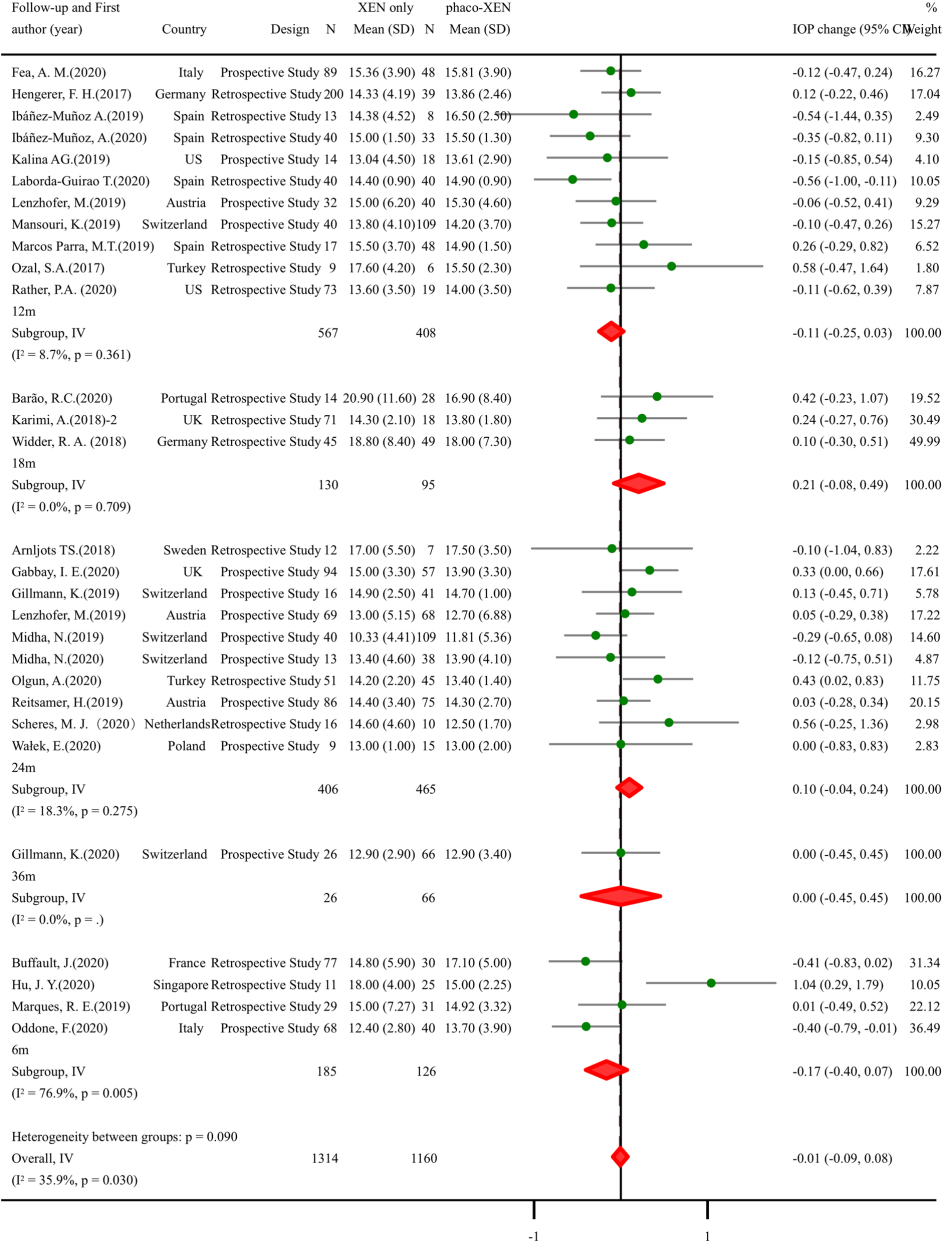
Meta-analysis of XEN-only surgery compared with phaco-XEN for IOP by follow-up duration after surgery. The total study sample included 1314 eyes undergoing XEN-only surgery and 1160 eyes undergoing phaco-XEN. Overall analysis of IOP after surgery (SMD: -0.01, 95% CI -0.09 to 0.08, *p* value 0.894)had no difference between two groups

**Fig. 4:**
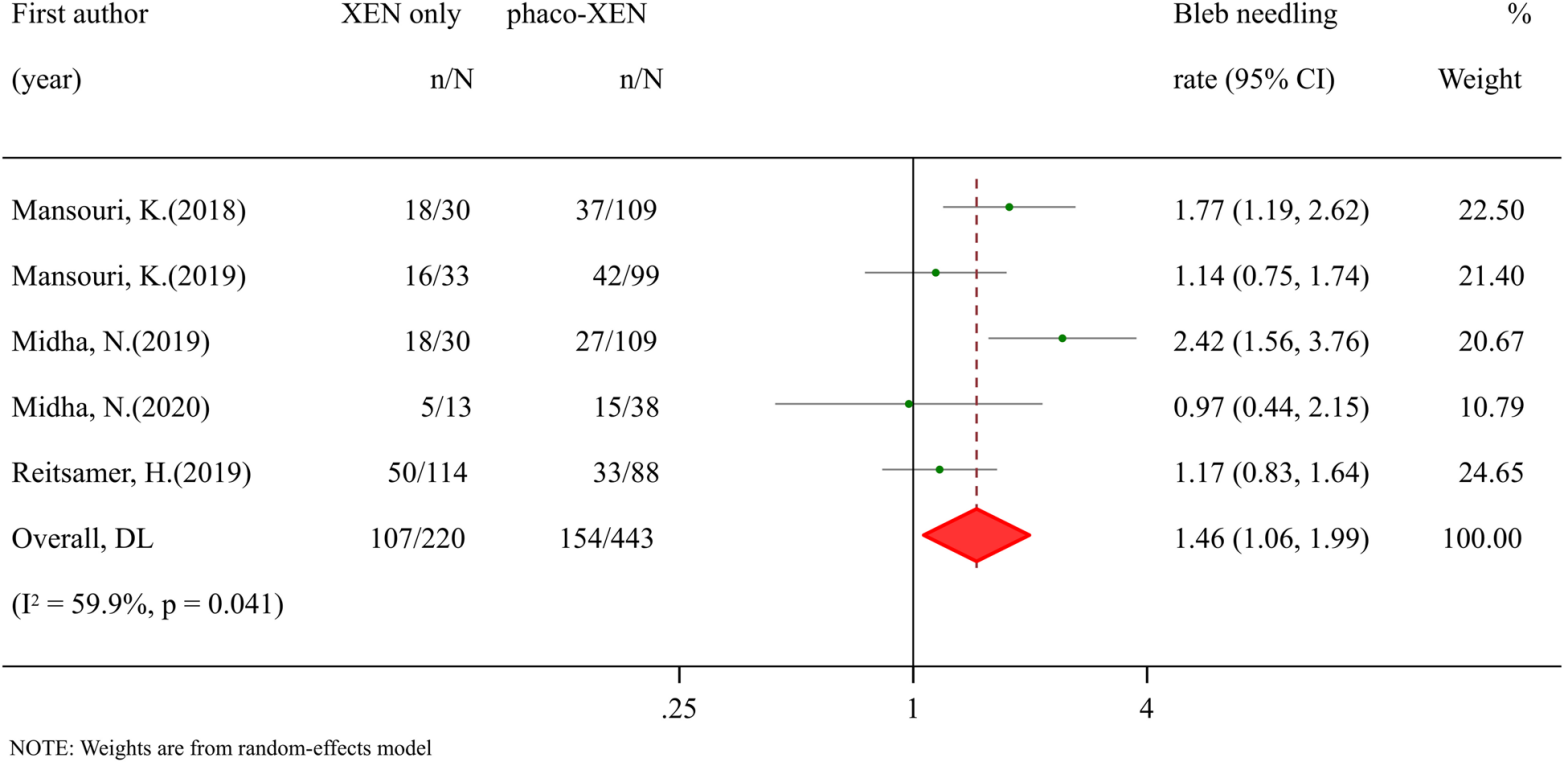
Meta-analysis of XEN-only surgery compared with phaco-XEN for bleb needing rate. The total study sample included 220 eyes undergoing XEN-only surgery and 443 eyes undergoing phaco-XEN surgery. Overall bleb needling rate was significantly high in XEN-only group (RR: 1.45, 95% CI 1.06to 1.99, *p* value 0.019)

In some centers, cataract surgery was done at the same time when XEN stent was being implanted (phaco-XEN). In glaucoma patients IOP dropped significantly after phaco-XEN surgery irrespective of ethnicity (SFig20) or follow-up duration (SFig21). Medication needed for lowering IOP also had a clear reduction (SFig22). Further comparison was done between XEN standalone surgery and phaco-XEN surgery on IOP and medication. After procedure, there was no significant difference in IOP (SMD: -0.01, 95% CI -0.09 to 0.08, *p* value 0.894) (sFig23) and NOAM (SMD: 0.09, 95% CI -0.04 to 0.23, *p* value 0.170) (Fig. 2) between two group. Stratified analysis was also done by ethnicity and follow-up duration for IOP. In Asian population a clear difference of after-surgery IOP was found between two procedures (SMD: 0.57, 95% CI 0.23 to 0.91), which was absent in both European and North American patients (SFig24). On different follow-up time points, patients in different procedures shared a similar IOP (Fig. 3). Nevertheless, lower IOP before surgery was found in phaco-XEN group when baseline was analyzed (SMD: 0.31, 95% CI 0.15to 0.47, *p* value < 0.001), especially in European population (SFig25). Patients whose IOP achieved < 18 mmHg, < 15 mmHg, < 12 mmHg or had a reduction > 20% from baseline were counted and RR of success rate was obtained, which showed no difference in efficacy of treatment between XEN alone and phaco-XEN (data not shown). For considering complications, bleb needling rate was compared. Although similar IOP reduction was found in XEN alone and phaco-XEN group, bleb needling rate was significantly high in XEN standalone group (RR: 1.45, 95% CI 1.06to 1.99, *p* value 0.019) (Fig. 4).

### Efficacy and safety comparisons between XEN standalone and trabeculectomy procedure

**Fig. 5:**
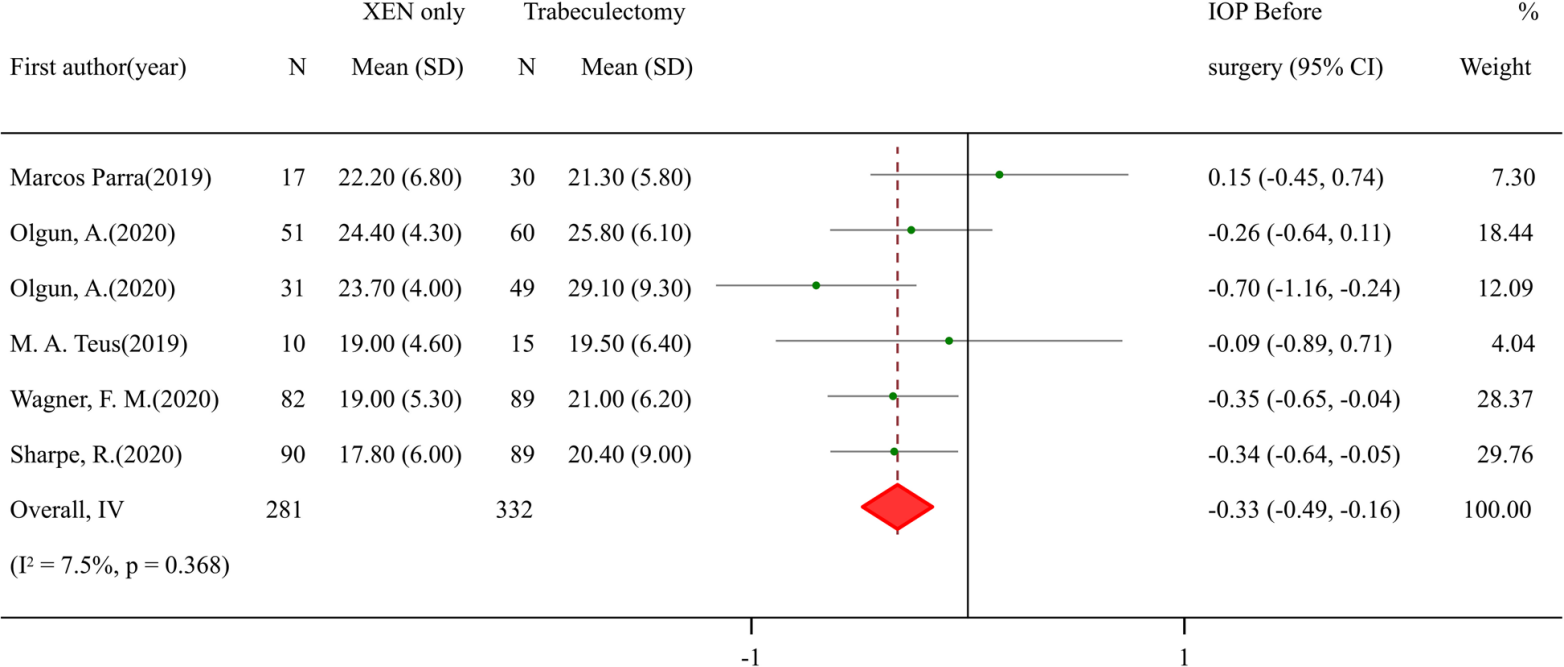
Meta-analysis of XEN surgery compared with trabeculectomy procedure for IOP before surgery. The total study sample included 281 eyes undergoing XEN-only surgery and 332 eyes undergoing trabeculectomy procedure. Overall analysis of IOP before surgery (SMD: -0.33, 95% CI -0.49 to -0.16, *p* value < 0.001) was lower in trabeculectomy procedure group

**Fig. 6:**
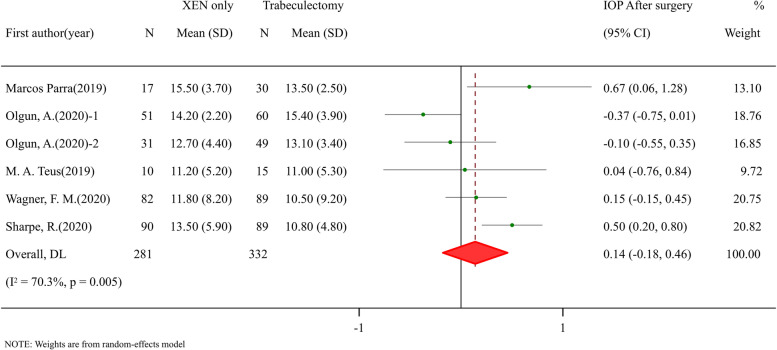
Meta-analysis of XEN surgery compared with trabeculectomy procedure for IOP after surgery. The total study sample included 281 eyes undergoing XEN-only surgery and 332 eyes undergoing trabeculectomy procedure. Overall analysis of IOP after surgery (SMD: 0.14, 95% CI -0.18 to 0.46, *p* value < 0.388) had no difference between two groups

**Fig. 7:**
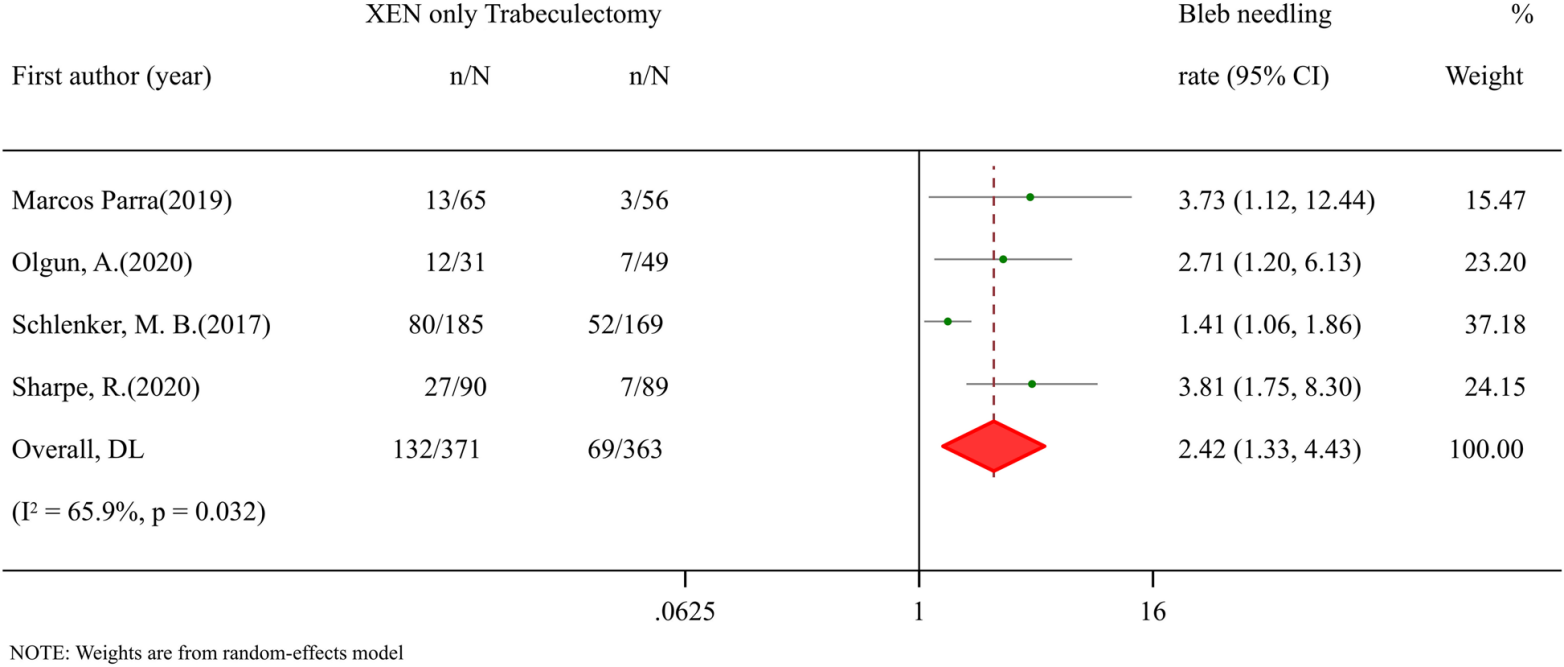
Meta-analysis of XEN surgery compared with trabeculectomy procedure for bleb needing rate. The total study sample included 371 eyes undergoing XEN-only surgery and 363 eyes undergoing trabeculectomy procedure. Overall bleb needling rate was significantly high in XEN-only group (RR: 2.42, 95% CI 1.33 to 4.43, *p* value 0.004)

Besides comparing to phaco-XEN, XEN standalone procedure was also compared with trabeculectomy surgery. A preference of assigning patients of higher IOP to trabeculectomy group was found when checking baseline (SMD: -0.33, 95% CI -0.49 to -0.16, *p* value < 0.001) (Fig. 5). After surgery, IOP showed no difference between two groups (Fig. 6), while patients underwent trabeculectomy had lower bleb needling rate (RR: 2.42, 95% CI 1.33 to 4.43, *p* value 0.004) (Fig. 7).

### Sensitivity analyses and publication bias

When leave-one-out sensitivity analyses were conducted, all the results remained statistically robust (Table 2, SFig26-33). *Egger* and *Begg* test was applied to test publication bias. Publication bias was found in IOP and medication comparison before and after phaco-XEN surgery. Publication bias was also found in after-surgery medication comparison of XEN standalone vs. phaco-XEN groups (Table 2). By trim and fill method, both the results of fixed and random effects model are the same with original result (Additional file 3: Appendix 3, SFig34-36), except for after-surgery medication comparison of XEN standalone vs. phaco-XEN groups.

**Table 2:** Sensitivity Analysis and Publication bias

	SMD Fluctuation	95% CI Fluctuation	Publication bias (*P* value)
**Before and after XEN surgery**			
IOP	1.65 ~ 1.71	1.49 ~ 1.88	0.298
Medication	2.06 ~ 2.14	1.80 ~ 2.41	0.597
**Before and after phaco-XEN surgery**			
IOP	1.60 ~ 1.72	1.37 ~ 1.99	0.007
Medication	2.13 ~ 2.28	1.78 ~ 2.65	0.048
**XEN vs. phaco-XEN surgery**			
IOP before surgery	0.25 ~ 0.33	0.11 ~ 0.50	0.162
IOP after surgery	-0.21 ~ 0.02	-0.13 ~ 0.13	0.405
Medication before surgery	0.28 ~ 0.35	0.11 ~ 0.54	0.970
Medication after surgery	0.82 ~ 0.14	-0.07 ~ 0.28	0.014

## Discussion

By screening through 725 research records and finally going into details of 78 clinical trials concerning XEN gel stent implantation in glaucoma, we conducted the most comprehensive meta-analysis ever since, in our knowledge. In this study, quantitative analyses were done to generate consolidated results, however, with no randomized clinical trial (RCT) available, elaborately designed RCTs should be carried out in the future for more convincing conclusions.

In this study, we were able to statistically evaluate the efficacy of XEN implantation in glaucoma in terms of IOP and NOAM. Both the measurements were effectively controlled within six months of XEN surgery and according to Lenzhofer. et al. [54] and Teus. et al. [87], IOP was maintained at a level of 13.40 ± 3.10 mmHg and 10.20 ± 5.20 mmHg 48 months after surgery. NOAM seems to increase with longer time points, follow-up period over 48 months is required to find out whether this is significant. Although there is genetic heterogeneity among different ethnicities concerning glaucoma morbidity, we did not find any difference when evaluating IOP or NOAM reduction efficacy of XEN surgery among African, European, North American, Oceanian, or Asian. Currently, most trials are on European and more clinical studies are in urgent need in other areas, especially for China mainland.

Besides POAG, XEN implantation is indicated to treat refractory POAG when previous treatments failed, and also for special types of OAG including PEXG, pigmentary glaucoma, juvenile glaucoma, and uveitic glaucoma. Studies stated that XEN is effective in treating both refractory glaucoma [93] and uveitic glaucoma [94], with bleb fibrosis, being the most common complications, which requires bleb needling. We did analysis on the four trials comparing XEN efficacy in POAG and PEXG and the results further confirmed that XEN implantation can reduce IOP and NOAM in PEXG as powerful as POAG.

Phacoemulsification is often combined with traditional filtering surgery such as TB, and phaco-XEN is possibly considered by clinicians while deciding the surgery. Whether phaco-XEN is superior to XEN-only or not has drawn attentions from a lot of studies. Thirty studies with totally over 1,000 eyes in each group were included in our analysis and we found no significant difference in IOP-lowering effect between XEN-only and phaco-XEN groups at the last follow-up. When we go into sub-analysis of various time points, XEN-only reveals lower after-surgery IOP than phaco-XEN in the short time points of 6 months and 12 months, the gap narrows with time, although this trend is of no significance. Lim. et al. [8] and Bo. et al. [9] did meta-analysis for closer time points of 1 day, 1 week, 1 month, 3 months, and 6 months, they showed that XEN-only has significant lower IOP than the combined group. Considering the time point of 6 months, our results are consistent with the above two meta-analysis that XEN-only has significant lower IOP than the combined group. We also found that NOAM and bleb needling rate was significantly lower in phaco-XEN than XEN-only, which indicates fewer fibrosis in combined group and phaco-XEN can be adopted with patients in high-risk of fibrosis. Traditional TB also showed lower bleb needling rate than XEN implantation, thus in our opinion, this new type of MIGS leads to worse bleb fibrosis although the gel is compatible in human tissue. It is also noteworthy that the endothelial cell density reduction in the phaco-XEN group was larger than in the XEN-onlygroup [63], and TB lose more endothelial cells than XEN [66].

Although our study shown that XEN is effective in lowering IOP till 48 months, at least three outstanding issues remain: Firstly, although we have tried but no unpublished data was found, so all included studies were published data. But Egger test showed no publication bias for most outcomes. Secondly, heterogeneity was high in some outcomes. Subgroup analysis was carried out, however, the source of heterogeneity is still not fully understood. Thirdly, the quality of included studies is relatively low. So long-term randomized control trials with large sample size are still in great need. The definition of outcomes are inconsistent in the 78 trials, which makes up publication bias and possible misinterpretation. Some trials reach complete success (expected IOL reduction without medications) when evaluating the XEN/phaco-XEN effects, while some reach qualified success (expected IOL reduction with medications). Besides, IOP targets of those trials are not same, for example, someIOP reduction > 30% while some targets of IOPreduction > 20%.These publication inconsistencies may lead to confoundings when comparing XEN/phaco-XEN effects.

## Conclusion

In this meta-analysis including 78 trials with thousands of eyes, we did the most comprehensive exploration ever on the efficacy of XEN implantation in treating glaucoma. To conclude, XEN is effective in both lowering IOP and NOAM till 48 months after surgery. It is also as effective in patients of PEXG as those of POAG, in terms of IOP, NOAM, and needling rate. Phaco-XEN may require fewer medications for patients after surgery, however the final IOP is similar to XEN-only surgery. It is noteworthy that XEN implantation leads to higher bleb fibrosis and needling rate, and phaco-XEN or TB may be a better choice to prevent filtering failure. Further studies on vision-threatening complications such as hypotony, choroidal leakage, and bleb infection comparing to other surgeries are in urgent need for evaluating safety of XEN implantation. Also, clinical trials on Asians are quite limited which restricts the application of XEN to a wider part of the world.

## Supplementary Information

**Additional file 1: Appendix 1.** PRISMA 2020 Checklist.

**Additional file 2: Appendix 2.**

**Additional file 3: Appendix 3.** 

**Additional file 4:SFig1.** Meta analysis of IOP by study design before and after XEN surgery. **SFig2.** Meta analysis of NOAM by study design before and after XEN surgery. **sFig3.** Meta analysis of IOP before and after XEN surgery. **sFig4.** Meta analysis of NOAM before and after XEN surgery. **sFig5.** Meta analysis of IOP by follow-up duration before and after XEN surgery. **sFig6.** Meta analysis of NOAM by follow-up duration before and after XEN surgery. **sFig7.** Meta analysis of IOP by ethnicity before and after XEN surgery. **sFig8.** Meta analysis of IOP by developed and developing country and after XEN surgery. **sFig9.** Meta analysis of NOAM by developed and developing country before and after XEN surgery. **sFig10.** Meta analysis of NOAM by ethnicity before after XEN surgery. **sFig11.** Meta analysis of IOP before XEN surgery betweem POAG and PEXG. **sFig12.** Meta analysis of IOP after XEN surgery betweem POAG and PEXG. **sFig13.** Meta analysis of NOAM before XEN surgery betweem POAG and PEXG. **sFig14.** Meta analysis of NOAM after XEN surgery betweem POAG and PEXG. **sFig15.** Meta analysis of IOP after XEN surgery between those with and without prior interventions. **sFig16.** Meta analysis of NOAM after XEN surgery between those with and without prior interventions. **sFig17.** Meta analysis of bleb needling rate  between those with and without prior interventions. **sFig18.** Meta analysis of IOP after XEN surgery between phakic and pseudophakic eyes. **sFig19.** Meta analysis of NOAM after XEN surgery between phakic and pseudophakic eyes. **sFig20.** Meta analysis of IOP by ethnicity before and after phaco-XEN surgery. **sFig21.** Meta analysis of IOP by follow-up duration before and after phaco-XEN surgery. **sFig22.** Meta analysis of NOAM before and after phaco-XEN surgery. **sFig23.** Meta analysis of IOP after surgery between XEN-only and phaco-XEN surgery. **sFig24.** Meta analysis of IOP after surgery by ethnicity between XEN-only and phaco-XEN surgery. **sFig25.** Meta analysis of IOP before surgery by ethnicity between XEN-only and phaco-XEN surgery. **sFig26.** sensitive analysis of IOP before and after XEN surgery. **sFig27.** sensitive analysis of NOAM before and after XEN surgery. **sFig28.** sensitive analysis of IOP before and after phaco-XEN surgery. **sFig29.** sensitive analysis of NOAM before and after phaco-XEN surgery. **sFig30.** sensitive analysis of IOP before XEN surgery and phaco-XEN surgery. **sFig31.** sensitive analysis of IOP after XEN surgery and phaco-XEN surgery. **sFig32.** sensitive analysis of NOAM before XEN surgery and phaco-XEN surgery. **sFig33.** sensitive analysis of NOAM after XEN surgery and phaco-XEN surgery. **sFig34.** filled funnel plot of IOP before and after phaco-XEN surgery. **sFig35.** filled funnel plot of NOAM before and after phaco-XEN surgery. **sFig36.** filled funnel plot of NOAM after XEN and phaco-XEN surgery. 

## Data Availability

Not published or shown elsewhere yet, not deposited in online database yet.
