# Supplementary material for: The efficacy of XEN gel stent implantation in glaucoma: a systematic review and meta-analysis
Source: BMC Ophthalmol. 2022 Jul 15;22:305. doi: 10.1186/s12886-022-02502-y (PMC9284889; doi:10.1186/s12886-022-02502-y)
Supplement: Supplementary file 2 — Additional file 2: Appendix 2. [file 12886_2022_2502_MOESM2_ESM.docx]

**Database: PubMed**

**Data: 5-15-2021**

**Records: 224**

((Glaucoma[Title/Abstract]) OR (Glaucoma[MeSH Terms])) AND (XEN implant [Title/Abstract]) OR Xen Gel Stent[Title/Abstract] OR XEN45 [Title/Abstract] OR XEN-45 [Title/Abstract] OR XEN gel implant [Title/Abstract] OR gel-stent [Title/Abstract] OR gel stent [Title/Abstract] OR gelatin stent [Title/Abstract] OR gelatin-stent [Title/Abstract] OR gelatin implant[Title/Abstract])

**Database: Cochrane library**

**Data: 5-15-2021**

**Records: 13**

([mh “Glaucoma”] OR 'Glaucoma':ab,ti) AND ('Xen Gel Stent':ab,ti OR 'XEN implant':ab,ti OR 'XEN45':ab,ti OR 'XEN-45':ab,ti OR 'XEN gel implant':ab,ti OR 'gel-stent':ab,ti OR 'gel stent ':ab,ti OR 'gelatin stent':ab,ti OR 'gelatin-stent':ab,ti OR 'gelatin implant':ab,ti)

**Database: Web of Science**

**Data: 5-15-2021**

**Records: 253**

TS=“Glaucoma” AND (TS=“Xen Gel Stent” OR TS=“Xen implant” OR TS=“XEN45” OR TS=“XEN-45” OR TS=“XEN gel implant” OR TS=“gel-stent” OR TS=“gel stent” OR TS=“gelatin stent” OR TS=“gelatin-stent” OR TS=“gelatin implant”)

**Database: Embase**

**Data: 5-15-2021**

**Records: 235**

('Glaucoma’/exp OR 'Glaucoma':ab,ti) AND ('Xen Gel Stent':ab,ti OR 'Xen implant':ab,ti OR 'XEN45':ab,ti OR 'XEN-45':ab,ti OR 'XEN gel implant':ab,ti OR 'gel-stent':ab,ti OR 'gel stent ':ab,ti OR 'gelatin stent':ab,ti OR 'gelatin-stent':ab,ti OR 'gelatin implant':ab,ti)

**Database: China National Knowledge Infrastructure (CNKI)**

**Data: 5-15-2021**

**Records:2**

SU='glaucoma' AND SU='XENgel stent’

**Database: Wanfang**

**Data: 5-15-2021**

**Records:5**

theme:(glaucoma) * theme:( XEN gel stent)

**Database: SinoMed**

**Data: 5-15-2021**

**Records:7**

"glaucoma" AND "XEN gel stent "
