# Supplementary material for: The efficacy of XEN gel stent implantation in glaucoma: a systematic review and meta-analysis
Source: BMC Ophthalmol. 2022 Jul 15;22:305. doi: 10.1186/s12886-022-02502-y (PMC9284889; doi:10.1186/s12886-022-02502-y)
Supplement: Supplementary file 3 — Additional file 3: Appendix 3. [file 12886_2022_2502_MOESM3_ESM.docx]

**Tirm and fill method**

**Before and after phaco-XEN surgery: IOP**

**Meta-analysis**

|  | Pooled | 95% CI | | Asymptotic | | No. of studies |
| --- | --- | --- | --- | --- | --- | --- |
| Method | Est | Lower | Upper | z_value | p_value |  |
| Fixed | 1.468 | 1.380 | 1.556 | 32.795 | 0.000 | 35 |
| Random | 1.684 | 1.418 | 1.950 | 12.418 | 0.000 |  |

**Filled Meta-analysis**

|  | Pooled | 95% CI | | Asymptotic | | No. of studies |
| --- | --- | --- | --- | --- | --- | --- |
| Method | Est | Lower | Upper | z_value | p_value |  |
| Fixed | 4.342 | 3.977 | 4.740 | 32.795 | 0.000 | 35 |
| Random | 5.389 | 4.131 | 7.030 | 12.418 | 0.000 |  |

*Explanation: By trim and fill method, both the results of fixed and random effects model are just the same with original result.*

**Before and after phaco-XEN surgery: Medication**

**Meta-analysis**

|  | Pooled | 95% CI | | Asymptotic | | No. of studies |
| --- | --- | --- | --- | --- | --- | --- |
| Method | Est | Lower | Upper | z_value | p_value |  |
| Fixed | 2.042 | 1.894 | 2.191 | 26.979 | 0.000 | 15 |
| Random | 2.208 | 1.850 | 2.565 | 12.098 | 0.000 |  |

**Filled Meta-analysis**

|  | Pooled | 95% CI | | Asymptotic | | No. of studies |
| --- | --- | --- | --- | --- | --- | --- |
| Method | Est | Lower | Upper | z_value | p_value |  |
| Fixed | 7.710 | 6.647 | 8.943 | 26.979 | 0.000 | 7 |
| Random | 9.096 | 6.361 | 13.007 | 12.787 | 0.000 |  |

*Explanation: By trim and fill method, both the results of fixed and random effects model are just the same with original result.*

**XEN vs. phaco-XEN: Medication after surgery**

**Meta-analysis**

|  | Pooled | 95% CI | | Asymptotic | | No. of studies |
| --- | --- | --- | --- | --- | --- | --- |
| Method | Est | Lower | Upper | z_value | p_value |  |
| Fixed | 0.093 | -0.040 | 0.226 | 1.372 | 0.170 | 10 |
| Random | 0.093 | -0.040 | 0.226 | 1.372 | 0.17 |  |

**Filled Meta-analysis**

|  | Pooled | 95% CI | | Asymptotic | | No. of studies |
| --- | --- | --- | --- | --- | --- | --- |
| Method | Est | Lower | Upper | z_value | p_value |  |
| Fixed | 1.057 | 0.931 | 1.199 | 0.859 | 0.390 | 13 |
| Random | 1.057 | 0.931 | 1.199 | 0.859 | 0.390 |  |

*Explanation: By trim and fill method, both the results of fixed and random effects model are just the same with original result.*
