# Supplementary material for: The efficacy of XEN gel stent implantation in glaucoma: a systematic review and meta-analysis
Source: BMC Ophthalmol. 2022 Jul 15;22:305. doi: 10.1186/s12886-022-02502-y (PMC9284889; doi:10.1186/s12886-022-02502-y)

SFig1 Meta analysis of IOP by study design before and after XEN surgery

Design and First author (year)	N	Before XEN Mean (SD)	N	After XEN Mean (SD)	IOP (95% CI)	% Weight
Prospective Study						
Cutolo CA.(2020)	93	26.40 (8.20)	93	13.40 (6.20)	1.79 (1.45, 2.13)	5.24
Fea, A. M.(2017)	21	21.79 (2.80)	21	14.90 (2.10)	2.78 (1.92, 3.64)	3.02
Fea, A. M.(2020)	115	25.03 (7.60)	89	15.36 (3.90)	1.54 (1.23, 1.86)	5.35
Gabbay, I. E.(2020)	94	22.90 (6.50)	94	15.00 (3.30)	1.53 (1.21, 1.86)	5.31
Gillmann, K.(2019)	16	20.50 (4.00)	16	14.90 (2.50)	1.68 (0.87, 2.49)	3.19
Gillmann, K.(2020)	26	21.00 (7.40)	26	12.90 (2.90)	1.44 (0.83, 2.05)	4.01
Gillmann, K.(2020)	37	19.00 (6.60)	37	13.70 (4.00)	0.97 (0.49, 1.45)	4.61
Grover, Ds(2017)	65	25.10 (3.70)	65	15.90 (5.20)	2.04 (1.61, 2.46)	4.87
Hong, K.(2020)	28	21.60 (6.60)	28	12.50 (3.60)	1.71 (1.10, 2.33)	4.00
Ilveskoski, L.(2017)	10	21.10 (7.30)	10	10.20 (3.60)	1.89 (0.82, 2.97)	2.36
Kalina AG.(2019)	20	24.18 (8.18)	14	13.04 (4.50)	1.61 (0.82, 2.40)	3.28
Lenzhofer, M.(2019)	64	22.50 (4.20)	64	13.40 (3.10)	2.47 (2.00, 2.93)	4.71
Lenzhofer, M.(2019)	69	22.50 (6.50)	69	13.00 (5.15)	1.62 (1.23, 2.01)	5.05
Lenzhofer, M.(2019)	32	22.20 (4.60)	32	15.00 (6.20)	1.32 (0.78, 1.86)	4.33
Mansouri, K.(2018)	57	19.80 (5.83)	57	13.90 (4.60)	1.12 (0.73, 1.52)	5.00
Mansouri, K.(2019)	40	21.20 (6.80)	40	13.80 (4.10)	1.32 (0.83, 1.80)	4.60
Midha, N.(2019)	40	20.05 (9.35)	40	10.33 (4.41)	1.33 (0.84, 1.82)	4.59
Midha, N.(2020)	13	23.00 (7.40)	13	13.40 (4.60)	1.56 (0.67, 2.44)	2.93
Oddone, F.(2020)	68	22.70 (4.30)	68	12.40 (2.80)	2.84 (2.36, 3.32)	4.63
Post, M.(2020)	20	21.56 (2.29)	20	17.69 (2.11)	1.76 (1.02, 2.49)	3.49
Reitsamer, H.(2019)	86	22.10 (4.00)	86	14.40 (3.40)	2.07 (1.70, 2.45)	5.11
Sheybani, A.(2016)	27	22.60 (4.11)	27	15.50 (3.65)	1.83 (1.19, 2.47)	3.90
Sng, C. C.(2018)	24	30.50 (9.80)	24	12.20 (3.10)	2.52 (1.75, 3.28)	3.37
Teixeira, F.J.(2021)	12	33.00 (9.00)	12	14.00 (3.00)	2.83 (1.67, 3.99)	2.14
Wałek, E.(2020)	9	25.00 (3.00)	9	13.00 (1.00)	5.37 (3.29, 7.44)	0.88
Subgroup, DL	1086		1054		1.80 (1.59, 2.02)	100.00
(I^2 = 73.8%, p = 0.000)						
Retrospective Study						
Arad T.(2019)	10	32.00 (7.50)	10	18.90 (2.60)	2.33 (1.17, 3.50)	1.72
Arnljots TS.(2018)	12	24.90 (7.40)	12	17.00 (5.50)	1.21 (0.34, 2.09)	2.11
Barão, R.C.(2020)	14	25.00 (10.60)	14	20.90 (11.60)	0.37 (-0.38, 1.12)	2.30
Başer, E. F.(2021)	29	24.55 (8.70)	29	15.03 (3.70)	1.42 (0.85, 2.00)	2.53
Bravetti, G.E.(2020)	60	29.90 (13.20)	47	15.20 (6.60)	1.36 (0.94, 1.78)	2.73
Buffault, J.(2020)	77	20.60 (6.60)	77	14.80 (5.90)	0.93 (0.59, 1.26)	2.83
Busch, T.(2020)	113	23.80 (6.20)	113	16.10 (4.70)	1.40 (1.11, 1.69)	2.86
Chao, Y.J.(2021)	37	21.70 (7.70)	37	15.00 (2.00)	1.19 (0.70, 1.69)	2.64
Dar, N.(2020)	23	22.60 (7.00)	23	16.70 (6.00)	0.91 (0.30, 1.51)	2.49
Do, A.(2020)	76	26.40 (8.40)	29	13.60 (6.30)	1.62 (1.14, 2.11)	2.66
Fernández-García, A.(2020)	40	18.02 (5.23)	40	14.62 (1.90)	0.86 (0.41, 1.32)	2.69
Fernández-García, A.(2020)	93	18.23 (5.00)	24	14.63 (1.91)	0.79 (0.33, 1.25)	2.69
Heidinger, A.(2019)	199	22.80 (6.90)	199	17.10 (5.90)	0.89 (0.68, 1.09)	2.93
Hengerer, F. H.(2017)	200	31.53 (8.36)	200	14.33 (4.19)	2.60 (2.33, 2.87)	2.88
Hengerer, F. H.(2018)	67	31.85 (8.50)	67	13.99 (2.60)	2.84 (2.36, 3.32)	2.66
Hengerer, F. H.(2019)	103	31.60 (8.90)	103	14.30 (4.20)	2.49 (2.12, 2.85)	2.79
Hu, J. Y.(2020)	19	24.00 (4.00)	11	18.00 (4.00)	1.50 (0.66, 2.34)	2.16
Ibáñez-Muñoz A.(2019)	13	20.85 (3.30)	13	14.38 (4.52)	1.63 (0.74, 2.53)	2.08
Ibáñez-Muñoz, A.(2020)	40	24.00 (3.00)	40	15.00 (1.50)	3.79 (3.06, 4.53)	2.31
Karimi, A.(2018)	17	21.50 (2.40)	17	13.60 (3.40)	2.68 (1.74, 3.63)	2.02
Karimi, A.(2018)	186	19.60 (2.00)	71	14.30 (2.10)	2.61 (2.26, 2.97)	2.80
Laborda-Guirao T.(2020)	40	21.80 (5.30)	40	14.40 (0.90)	1.95 (1.41, 2.48)	2.59
Laroche, D.(2019)	12	15.30 (6.20)	12	12.90 (4.50)	0.44 (-0.37, 1.25)	2.20
Linton, E.(2020)	151	26.10 (8.30)	149	16.30 (3.70)	1.52 (1.26, 1.78)	2.89
Marcos Parra, M.T.(2019)	17	22.20 (6.80)	17	15.50 (3.70)	1.22 (0.49, 1.96)	2.31
Marques, R. E.(2019)	29	25.52 (8.11)	29	15.00 (7.27)	1.37 (0.79, 1.94)	2.54
Olgun, A.(2020)	51	24.40 (4.30)	51	14.20 (2.20)	2.99 (2.42, 3.55)	2.55
Ozal, S.A.(2017)	9	36.70 (4.10)	9	17.60 (4.20)	4.60 (2.76, 6.44)	1.05
Qureshi, A.(2019)	37	36.10 (9.60)	37	12.60 (4.10)	3.18 (2.49, 3.88)	2.38
Rather, P.A.(2020)	73	16.80 (5.40)	73	13.60 (3.50)	0.70 (0.37, 1.04)	2.82
Rauchegger, T(2020)	79	23.40 (7.90)	79	14.60 (3.60)	1.43 (1.08, 1.78)	2.81
Schargus, M.(2020)	113	22.80 (6.50)	113	15.10 (4.60)	1.37 (1.08, 1.66)	2.86
Scheres, M. J.(2020)	26	19.60 (4.90)	16	14.60 (4.60)	1.04 (0.38, 1.71)	2.42
Smith, M.(2019)	68	21.10 (6.40)	68	14.80 (5.10)	1.09 (0.73, 1.45)	2.80
Tan, N.E.(2021)	50	23.52 (8.53)	36	14.72 (0.79)	1.35 (0.87, 1.82)	2.67
Tan, S. Z.(2018)	43	24.90 (7.80)	43	14.50 (3.40)	1.73 (1.23, 2.23)	2.64
Teus, M.A.(2019)	10	19.00 (4.60)	10	10.20 (5.20)	1.79 (0.74, 2.85)	1.86
Theillac, V.(2020)	47	20.80 (6.80)	47	16.20 (2.80)	0.88 (0.46, 1.31)	2.73
Urcola, A.(2021)	18	25.20 (7.90)	18	15.10 (3.30)	1.67 (0.90, 2.43)	2.27
Widder, R. A.(2018)	45	25.20 (7.30)	45	18.80 (8.40)	0.81 (0.38, 1.24)	2.72
Subgroup, DL	2346		2068		1.60 (1.37, 1.84)	100.00
(I^2 = 90.3%, p = 0.000)						
Heterogeneity between groups: p = 0.212						
Overall, DL	3432		3122		1.69 (1.52, 1.86)	
(I^2 = 87.4%, p = 0.000)						

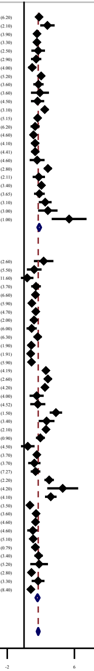

NOTE: Weights and between-subgroup heterogeneity test are from random-effects model

SFig2 Meta analysis of NOAM by study design before and after XEN surgery

Design and First author (year)	Country	Design	N	Before XEN Mean (SD)	N	After XEN Mean (SD)	Medication (95% CI)	% Weight
Prospective Study		Prospective Study						
Cutolo CA.(2020)	Italy	Prospective Study	93	2.90 (1.00)	93	0.38 (0.95)	2.58 (2.19, 2.97)	8.07
Fea, A. M.(2017)	Italy	Prospective Study	21	2.92 (1.16)	21	0.50 (0.53)	2.68 (1.84, 3.53)	5.87
Fea, A. M.(2020)	Italy	Prospective Study	115	3.25 (1.10)	89	0.53 (1.00)	2.57 (2.20, 2.95)	8.14
Gabbay, I. E.(2020)	UK	Prospective Study	94	2.80 (1.10)	94	0.40 (0.90)	2.39 (2.01, 2.76)	8.13
Gillmann, K.(2020)	Switzerland	Prospective Study	37	2.16 (1.14)	37	0.46 (0.77)	1.75 (1.21, 2.29)	7.39
Grover, Ds(2017)	US	Prospective Study	65	3.50 (1.00)	65	1.70 (1.50)	1.41 (1.03, 1.80)	8.09
Ilveskoski, L.(2017)	Finland	Prospective Study	10	2.40 (1.10)	10	0.82 (0.30)	1.96 (0.87, 3.05)	4.79
Kalina AG.(2019)	US	Prospective Study	20	2.96 (1.20)	14	0.75 (1.27)	1.80 (0.99, 2.61)	6.02
Lenzhofer, M.(2019)	Austria	Prospective Study	64	2.40 (1.30)	64	1.20 (1.30)	0.92 (0.56, 1.29)	8.17
Mansouri, K.(2018)	Switzerland	Prospective Study	57	1.98 (1.16)	57	0.40 (0.80)	1.59 (1.16, 2.01)	7.93
Post, M.(2020)	Poland	Prospective Study	20	3.20 (0.77)	20	1.60 (0.99)	1.80 (1.06, 2.54)	6.38
Reitsamer, H.(2019)	Austria	Prospective Study	114	2.70 (0.90)	86	1.20 (1.20)	1.44 (1.13, 1.76)	8.37
Sheybani, A.(2016)	US	Prospective Study	27	2.70 (1.00)	27	1.50 (0.50)	1.52 (0.91, 2.13)	7.04
Sng, C. C.(2018)	UK	Prospective Study	24	3.30 (0.80)	24	0.40 (0.90)	3.41 (2.51, 4.30)	5.62
Subgroup, DL		Prospective Study	761		701		1.95 (1.60, 2.30)	100.00
(I² = 85.6%, p = 0.000)		Prospective Study						
		Prospective Study						
Retrospective Study		Retrospective Study						
Barão, R.C.(2020)	Portugal	Retrospective Study	14	2.93 (0.92)	14	1.07 (0.92)	2.02 (1.10, 2.94)	3.28
Başer, E. F.(2021)	Turkey	Retrospective Study	29	2.83 (0.80)	29	0.86 (1.30)	1.83 (1.21, 2.44)	3.68
Bravetti, G.E.(2020)	Switzerland	Retrospective Study	60	3.00 (1.30)	47	1.30 (0.90)	1.49 (1.06, 1.92)	3.88
Busch, T.(2020)	Sweden	Retrospective Study	113	3.40 (1.00)	113	1.34 (0.80)	2.27 (1.94, 2.61)	3.96
Chao, Y.J.(2021)	China	Retrospective Study	37	3.40 (0.90)	37	1.30 (1.50)	1.70 (1.16, 2.23)	3.78
Dar, N.(2020)	Israel	Retrospective Study	23	3.58 (0.53)	23	0.36 (0.80)	4.75 (3.60, 5.89)	2.95
Do, A.(2020)	US	Retrospective Study	76	3.60 (0.94)	29	0.90 (1.40)	2.49 (1.94, 3.04)	3.76
Fernández-García, A.(2020)	Spain	Retrospective Study	40	1.60 (0.94)	40	1.00 (0.87)	0.66 (0.21, 1.11)	3.87
Fernández-García, A.(2020)	Spain	Retrospective Study	93	1.87 (0.94)	14	1.00 (0.88)	0.93 (0.36, 1.51)	3.73
Heidinger, A.(2019)	Austria	Retrospective Study	119	2.90 (1.00)	119	1.80 (1.40)	0.90 (0.64, 1.17)	4.01
Hengerer, F. H.(2017)	Germany	Retrospective Study	200	3.09 (1.00)	200	0.30 (0.68)	3.26 (2.96, 3.56)	3.99
Hengerer, F. H.(2018)	Germany	Retrospective Study	67	3.25 (0.80)	67	0.30 (0.70)	3.92 (3.34, 4.51)	3.72
Hengerer, F. H.(2019)	Germany	Retrospective Study	103	3.00 (1.00)	103	0.30 (0.70)	3.13 (2.72, 3.54)	3.90
Hu, J. Y.(2020)	Singapore	Retrospective Study	19	4.00 (1.00)	11	1.08 (1.15)	2.77 (1.73, 3.80)	3.11
Ibáñez-Muñoz A.(2019)	Spain	Retrospective Study	13	3.00 (1.10)	13	1.30 (1.10)	1.55 (0.66, 2.43)	3.33
Karimi, A.(2018)	UK	Retrospective Study	17	2.80 (0.60)	17	1.00 (1.30)	1.78 (0.98, 2.58)	3.44
Laborda-Guirao T.(2020)	Spain	Retrospective Study	40	2.90 (0.62)	40	1.80 (0.40)	2.11 (1.56, 2.66)	3.76
Laroche, D.(2019)	US	Retrospective Study	12	3.58 (0.70)	12	1.75 (1.50)	1.56 (0.64, 2.49)	3.27
Marcos Parra, M.T.(2019)	Spain	Retrospective Study	17	2.50 (0.80)	17	0.20 (0.60)	3.25 (2.21, 4.30)	3.10
Olgun, A.(2020)	Turkey	Retrospective Study	51	3.40 (0.50)	51	2.00 (2.20)	0.88 (0.47, 1.28)	3.91
Ozal, S.A.(2017)	Turkey	Retrospective Study	9	3.70 (0.50)	9	0.60 (1.10)	3.63 (2.07, 5.19)	2.39
Qureshi, A.(2019)	UK	Retrospective Study	37	3.69 (0.47)	37	0.62 (1.10)	3.63 (2.88, 4.38)	3.52
Rauchegger, T(2020)	Austria	Retrospective Study	79	2.70 (1.10)	79	1.00 (1.20)	1.48 (1.12, 1.83)	3.95
Schargus, M.(2020)	Germany	Retrospective Study	113	2.50 (1.20)	113	0.80 (1.30)	1.36 (1.07, 1.65)	4.00
Scheres, M. J.（2020）	Netherlands	Retrospective Study	26	2.70 (1.30)	16	1.10 (1.50)	1.16 (0.49, 1.83)	3.61
Smith, M.(2019)	UK	Retrospective Study	68	2.90 (0.80)	68	1.10 (1.10)	1.87 (1.47, 2.28)	3.91
Theillac, V.(2020)	France	Retrospective Study	47	2.66 (1.10)	47	0.49 (1.00)	2.06 (1.56, 2.57)	3.81
Urcola, A.(2021)	Spain	Retrospective Study	18	2.90 (0.30)	18	0.40 (0.50)	6.06 (4.48, 7.65)	2.36
Subgroup, DL		Retrospective Study	1540		1383		2.20 (1.83, 2.58)	100.00
(I² = 93.3%, p = 0.000)		Retrospective Study						
		Retrospective Study						
Heterogeneity between groups: p = 0.332								
Overall, DL			2301		2084		2.11 (1.84, 2.38)	
(I² = 91.7%, p = 0.000)								

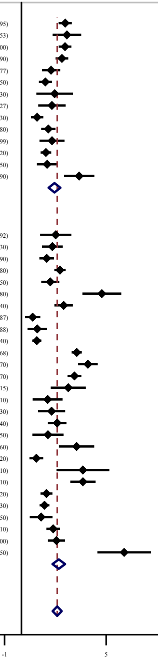

NOTE: Weights and between-subgroup heterogeneity test are from random-effects model

sFig3 Meta analysis of IOP before and after XEN surgery

First author (year)	Country	Design	Before XEN N	Before XEN Mean (SD)	After XEN N	After XEN Mean (SD)	IOP change (95% CI)	% Weight
Lenzhofer, M.(2019)	Austria	Prospective Study	64	22.50 (4.20)	64	13.40 (3.10)	2.47 (2.00, 2.93)	1.69
Lenzhofer, M.(2019)	Austria	Prospective Study	69	22.50 (6.50)	69	13.00 (5.15)	1.62 (1.23, 2.01)	1.76
Lenzhofer, M.(2019)	Austria	Prospective Study	32	22.20 (4.60)	32	15.00 (6.20)	1.32 (0.78, 1.86)	1.61
Reitsamer, H.(2019)	Austria	Prospective Study	86	22.10 (4.00)	86	14.40 (3.40)	2.07 (1.70, 2.45)	1.77
Ilveskoski, L.(2017)	Finland	Prospective Study	10	21.10 (7.30)	10	10.20 (3.60)	1.89 (0.82, 2.97)	1.09
Cutolo CA.(2020)	Italy	Prospective Study	93	26.40 (8.20)	93	13.40 (6.20)	1.79 (1.45, 2.13)	1.79
Fea, A. M.(2017)	Italy	Prospective Study	21	21.79 (2.80)	21	14.90 (2.10)	2.78 (1.92, 3.64)	1.29
Fea, A. M.(2020)	Italy	Prospective Study	115	25.03 (7.60)	89	15.36 (3.90)	1.54 (1.23, 1.86)	1.81
Oddone, F.(2020)	Italy	Prospective Study	68	22.70 (4.30)	68	12.40 (2.80)	2.84 (2.36, 3.32)	1.67
Post, M.(2020)	Poland	Prospective Study	20	21.56 (2.29)	20	17.69 (2.11)	1.76 (1.02, 2.49)	1.42
Wałek, E.(2020)	Poland	Prospective Study	9	25.00 (3.00)	9	13.00 (1.00)	5.37 (3.29, 7.44)	0.50
Teixeira, F.J.(2021)	Portugal	Prospective Study	12	33.00 (9.00)	12	14.00 (3.00)	2.83 (1.67, 3.99)	1.02
Gillmann, K.(2019)	Switzerland	Prospective Study	16	20.50 (4.00)	16	14.90 (2.50)	1.68 (0.87, 2.49)	1.34
Gillmann, K.(2020)	Switzerland	Prospective Study	26	21.00 (7.40)	26	12.90 (2.90)	1.44 (0.83, 2.05)	1.54
Gillmann, K.(2020)	Switzerland	Prospective Study	37	19.00 (6.60)	37	13.70 (4.00)	0.97 (0.49, 1.45)	1.67
Mansouri, K.(2018)	Switzerland	Prospective Study	57	19.80 (5.83)	57	13.90 (4.60)	1.12 (0.73, 1.52)	1.75
Mansouri, K.(2019)	Switzerland	Prospective Study	40	21.20 (6.80)	40	13.80 (4.10)	1.32 (0.83, 1.80)	1.67
Midha, N.(2019)	Switzerland	Prospective Study	40	20.05 (9.35)	40	10.33 (4.41)	1.33 (0.84, 1.82)	1.67
Midha, N.(2020)	Switzerland	Prospective Study	13	23.00 (7.40)	13	13.40 (4.60)	1.56 (0.67, 2.44)	1.26
Gabbay, I. E.(2020)	UK	Prospective Study	94	22.90 (6.50)	94	15.00 (3.30)	1.53 (1.21, 1.86)	1.80
Sng, C. C.(2018)	UK	Prospective Study	24	30.50 (9.80)	24	12.20 (3.10)	2.52 (1.75, 3.28)	1.39
Grover, Ds(2017)	US	Prospective Study	65	25.10 (3.70)	65	15.90 (5.20)	2.04 (1.61, 2.46)	1.72
Hong, K.(2020)	US	Prospective Study	28	21.60 (6.60)	28	12.50 (3.60)	1.71 (1.10, 2.33)	1.54
Kalina AG.(2019)	US	Prospective Study	20	24.18 (8.18)	14	13.04 (4.50)	1.61 (0.82, 2.40)	1.36
Sheybani, A.(2016)	US	Prospective Study	27	22.60 (4.11)	27	15.50 (3.65)	1.83 (1.19, 2.47)	1.52
Heidinger, A.(2019)	Austria	Retrospective Study	199	22.80 (6.90)	199	17.10 (5.90)	0.89 (0.68, 1.09)	1.88
Rauchegger, T(2020)	Austria	Retrospective Study	79	23.40 (7.90)	79	14.60 (3.60)	1.43 (1.08, 1.78)	1.78
Chao, Y.J.(2021)	China	Retrospective Study	37	21.70 (7.70)	37	15.00 (2.00)	1.19 (0.70, 1.69)	1.66
Buffault, J.(2020)	France	Retrospective Study	77	20.60 (6.60)	77	14.80 (5.90)	0.93 (0.59, 1.26)	1.80
Theillac, V.(2020)	France	Retrospective Study	47	20.80 (6.80)	47	16.20 (2.80)	0.88 (0.46, 1.31)	1.72
Arad T.(2019)	Germany	Retrospective Study	10	32.00 (7.50)	10	18.90 (2.60)	2.33 (1.17, 3.50)	1.01
Hengerer, F. H.(2017)	Germany	Retrospective Study	200	31.53 (8.36)	200	14.33 (4.19)	2.60 (2.33, 2.87)	1.84
Hengerer, F. H.(2018)	Germany	Retrospective Study	67	31.85 (8.50)	67	13.99 (2.60)	2.84 (2.36, 3.32)	1.67
Hengerer, F. H.(2019)	Germany	Retrospective Study	103	31.60 (8.90)	103	14.30 (4.20)	2.49 (2.12, 2.85)	1.77
Schargus, M.(2020)	Germany	Retrospective Study	113	22.80 (6.50)	113	15.10 (4.60)	1.37 (1.08, 1.66)	1.83
Widder, R. A.(2018)	Germany	Retrospective Study	45	25.20 (7.30)	45	18.80 (8.40)	0.81 (0.38, 1.24)	1.72
Dar, N.(2020)	Israel	Retrospective Study	23	22.60 (7.00)	23	16.70 (6.00)	0.91 (0.30, 1.51)	1.55
Scheres, M. J.(2020)	Netherlands	Retrospective Study	26	19.60 (4.90)	16	14.60 (4.60)	1.04 (0.38, 1.71)	1.49
Barão, R.C.(2020)	Portugal	Retrospective Study	14	25.00 (10.60)	14	20.90 (11.60)	0.37 (-0.38, 1.12)	1.40
Marques, R. E.(2019)	Portugal	Retrospective Study	29	25.52 (8.11)	29	15.00 (7.27)	1.37 (0.79, 1.94)	1.58
Hu, J. Y.(2020)	Singapore	Retrospective Study	19	24.00 (4.00)	11	18.00 (4.00)	1.50 (0.66, 2.34)	1.31
Fernández-García, A.(2020)	Spain	Retrospective Study	40	18.02 (5.23)	40	14.62 (1.90)	0.86 (0.41, 1.32)	1.69
Fernández-García, A.(2020)	Spain	Retrospective Study	93	18.23 (5.00)	24	14.63 (1.91)	0.79 (0.33, 1.25)	1.69
Ibáñez-Muñoz A.(2019)	Spain	Retrospective Study	13	20.85 (3.30)	13	14.38 (4.52)	1.63 (0.74, 2.53)	1.25
Ibáñez-Muñoz, A.(2020)	Spain	Retrospective Study	40	24.00 (3.00)	40	15.00 (1.50)	3.79 (3.06, 4.53)	1.41
Laborda-Guirao T.(2020)	Spain	Retrospective Study	40	21.80 (5.30)	40	14.40 (0.90)	1.95 (1.41, 2.48)	1.62
Marcos Parra, M.T.(2019)	Spain	Retrospective Study	17	22.20 (6.80)	17	15.50 (3.70)	1.22 (0.49, 1.96)	1.42
Teus, M.A.(2019)	Spain	Retrospective Study	10	19.00 (4.60)	10	10.20 (5.20)	1.79 (0.74, 2.85)	1.11
Urcola, A.(2021)	Spain	Retrospective Study	18	25.20 (7.90)	18	15.10 (3.30)	1.67 (0.90, 2.43)	1.39
Arnljots TS.(2018)	Sweden	Retrospective Study	12	24.90 (7.40)	12	17.00 (5.50)	1.21 (0.34, 2.09)	1.27
Busch, T.(2020)	Sweden	Retrospective Study	113	23.80 (6.20)	113	16.10 (4.70)	1.40 (1.11, 1.69)	1.83
Bravetti, G.E.(2020)	Switzerland	Retrospective Study	60	29.90 (13.20)	47	15.20 (6.60)	1.36 (0.94, 1.78)	1.72
Başer, E. F.(2021)	Turkey	Retrospective Study	29	24.55 (8.70)	29	15.03 (3.70)	1.42 (0.85, 2.00)	1.58
Olgun, A.(2020)	Turkey	Retrospective Study	51	24.40 (4.30)	51	14.20 (2.20)	2.99 (2.42, 3.55)	1.59
Ozal, S.A.(2017)	Turkey	Retrospective Study	9	36.70 (4.10)	9	17.60 (4.20)	4.60 (2.76, 6.44)	0.59
Karimi, A.(2018)	UK	Retrospective Study	17	21.50 (2.40)	17	13.60 (3.40)	2.68 (1.74, 3.63)	1.21
Karimi, A.(2018)	UK	Retrospective Study	186	19.60 (2.00)	71	14.30 (2.10)	2.61 (2.26, 2.97)	1.78
Linton, E.(2020)	UK	Retrospective Study	151	26.10 (8.30)	149	16.30 (3.70)	1.52 (1.26, 1.78)	1.85
Qureshi, A.(2019)	UK	Retrospective Study	37	36.10 (9.60)	37	12.60 (4.10)	3.18 (2.49, 3.88)	1.46
Smith, M.(2019)	UK	Retrospective Study	68	21.10 (6.40)	68	14.80 (5.10)	1.09 (0.73, 1.45)	1.78
Tan, S. Z.(2018)	UK	Retrospective Study	43	24.90 (7.80)	43	14.50 (3.40)	1.73 (1.23, 2.23)	1.66
Do, A.(2020)	US	Retrospective Study	76	26.40 (8.40)	29	13.60 (6.30)	1.62 (1.14, 2.11)	1.67
Laroche, D.(2019)	US	Retrospective Study	12	15.30 (6.20)	12	12.90 (4.50)	0.44 (-0.37, 1.25)	1.34
Rather, P.A.(2020)	US	Retrospective Study	73	16.80 (5.40)	73	13.60 (3.50)	0.70 (0.37, 1.04)	1.80
Tan, N.E.(2021)	US	Retrospective Study	50	23.52 (8.53)	36	14.72 (0.79)	1.35 (0.87, 1.82)	1.68
Overall, DL ($I^2 = 87.4\%$, $p = 0.000$)			3432		3122		1.69 (1.52, 1.86)	100.00

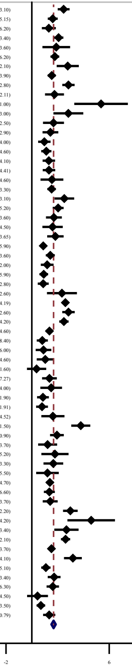

NOTE: Weights are from random-effects model

sFig4 Meta analysis of NOAM before and after XEN surgery

First author (year)	Country	Design	N	Before XEN Mean (SD)	N	After XEN Mean (SD)	Medication (95% CI)	% Weight
Lenzhofer, M.(2019)	Austria	Prospective Study	64	2.40 (1.30)	64	1.20 (1.30)	0.92 (0.56, 1.29)	2.65
Reitsamer, H.(2019)	Austria	Prospective Study	114	2.70 (0.90)	86	1.20 (1.20)	1.44 (1.13, 1.76)	2.69
Ilveskoski, L.(2017)	Finland	Prospective Study	10	2.40 (1.10)	10	0.82 (0.30)	1.96 (0.87, 3.05)	1.92
Cutolo CA.(2020)	Italy	Prospective Study	93	2.90 (1.00)	93	0.38 (0.95)	2.58 (2.19, 2.97)	2.63
Fea, A. M.(2017)	Italy	Prospective Study	21	2.92 (1.16)	21	0.50 (0.53)	2.68 (1.84, 3.53)	2.19
Fea, A. M.(2020)	Italy	Prospective Study	115	3.25 (1.10)	89	0.53 (1.00)	2.57 (2.20, 2.95)	2.65
Post, M.(2020)	Poland	Prospective Study	20	3.20 (0.77)	20	1.60 (0.99)	1.80 (1.06, 2.54)	2.30
Gillmann, K.(2020)	Switzerland	Prospective Study	37	2.16 (1.14)	37	0.46 (0.77)	1.75 (1.21, 2.29)	2.51
Mansouri, K.(2018)	Switzerland	Prospective Study	57	1.98 (1.16)	57	0.40 (0.80)	1.59 (1.16, 2.01)	2.61
Gabbay, I. E.(2020)	UK	Prospective Study	94	2.80 (1.10)	94	0.40 (0.90)	2.39 (2.01, 2.76)	2.65
Sng, C. C.(2018)	UK	Prospective Study	24	3.30 (0.80)	24	0.40 (0.90)	3.41 (2.51, 4.30)	2.13
Grover, Ds(2017)	US	Prospective Study	65	3.50 (1.00)	65	1.70 (1.50)	1.41 (1.03, 1.80)	2.64
Kalina AG.(2019)	US	Prospective Study	20	2.96 (1.20)	14	0.75 (1.27)	1.80 (0.99, 2.61)	2.22
Sheybani, A.(2016)	US	Prospective Study	27	2.70 (1.00)	27	1.50 (0.50)	1.52 (0.91, 2.13)	2.44
Heidinger, A.(2019)	Austria	Retrospective Study	119	2.90 (1.00)	119	1.80 (1.40)	0.90 (0.64, 1.17)	2.71
Rauchegger, T(2020)	Austria	Retrospective Study	79	2.70 (1.10)	79	1.00 (1.20)	1.48 (1.12, 1.83)	2.66
Chao, Y.J.(2021)	China	Retrospective Study	37	3.40 (0.90)	37	1.30 (1.50)	1.70 (1.16, 2.23)	2.51
Theillac, V.(2020)	France	Retrospective Study	47	2.66 (1.10)	47	0.49 (1.00)	2.06 (1.56, 2.57)	2.54
Hengerer, F. H.(2017)	Germany	Retrospective Study	200	3.09 (1.00)	200	0.30 (0.68)	3.26 (2.96, 3.56)	2.70
Hengerer, F. H.(2018)	Germany	Retrospective Study	67	3.25 (0.80)	67	0.30 (0.70)	3.92 (3.34, 4.51)	2.47
Hengerer, F. H.(2019)	Germany	Retrospective Study	103	3.00 (1.00)	103	0.30 (0.70)	3.13 (2.72, 3.54)	2.62
Schargus, M.(2020)	Germany	Retrospective Study	113	2.50 (1.20)	113	0.80 (1.30)	1.36 (1.07, 1.65)	2.70
Dar, N.(2020)	Israel	Retrospective Study	23	3.58 (0.53)	23	0.36 (0.80)	4.75 (3.60, 5.89)	1.85
Scheres, M. J.（2020）	Netherlands	Retrospective Study	26	2.70 (1.30)	16	1.10 (1.50)	1.16 (0.49, 1.83)	2.37
Barão, R.C.(2020)	Portugal	Retrospective Study	14	2.93 (0.92)	14	1.07 (0.92)	2.02 (1.10, 2.94)	2.10
Hu, J. Y.(2020)	Singapore	Retrospective Study	19	4.00 (1.00)	11	1.08 (1.15)	2.77 (1.73, 3.80)	1.97
Fernández-García, A.(2020)	Spain	Retrospective Study	40	1.60 (0.94)	40	1.00 (0.87)	0.66 (0.21, 1.11)	2.59
Fernández-García, A.(2020)	Spain	Retrospective Study	93	1.87 (0.94)	14	1.00 (0.88)	0.93 (0.36, 1.51)	2.47
Ibáñez-Muñoz A.(2019)	Spain	Retrospective Study	13	3.00 (1.10)	13	1.30 (1.10)	1.55 (0.66, 2.43)	2.14
Laborda-Guirao T.(2020)	Spain	Retrospective Study	40	2.90 (0.62)	40	1.80 (0.40)	2.11 (1.56, 2.66)	2.50
Marcos Parra, M.T.(2019)	Spain	Retrospective Study	17	2.50 (0.80)	17	0.20 (0.60)	3.25 (2.21, 4.30)	1.96
Urcola, A.(2021)	Spain	Retrospective Study	18	2.90 (0.30)	18	0.40 (0.50)	6.06 (4.48, 7.65)	1.42
Busch, T.(2020)	Sweden	Retrospective Study	113	3.40 (1.00)	113	1.34 (0.80)	2.27 (1.94, 2.61)	2.67
Bravetti, G.E.(2020)	Switzerland	Retrospective Study	60	3.00 (1.30)	47	1.30 (0.90)	1.49 (1.06, 1.92)	2.60
Başer, E. F.(2021)	Turkey	Retrospective Study	29	2.83 (0.80)	29	0.86 (1.30)	1.83 (1.21, 2.44)	2.43
Olgun, A.(2020)	Turkey	Retrospective Study	51	3.40 (0.50)	51	2.00 (2.20)	0.88 (0.47, 1.28)	2.62
Ozal, S.A.(2017)	Turkey	Retrospective Study	9	3.70 (0.50)	9	0.60 (1.10)	3.63 (2.07, 5.19)	1.44
Karimi, A.(2018)	UK	Retrospective Study	17	2.80 (0.60)	17	1.00 (1.30)	1.78 (0.98, 2.58)	2.23
Qureshi, A.(2019)	UK	Retrospective Study	37	3.69 (0.47)	37	0.62 (1.10)	3.63 (2.88, 4.38)	2.29
Smith, M.(2019)	UK	Retrospective Study	68	2.90 (0.80)	68	1.10 (1.10)	1.87 (1.47, 2.28)	2.62
Do, A.(2020)	US	Retrospective Study	76	3.60 (0.94)	29	0.90 (1.40)	2.49 (1.94, 3.04)	2.50
Laroche, D.(2019)	US	Retrospective Study	12	3.58 (0.70)	12	1.75 (1.50)	1.56 (0.64, 2.49)	2.10
Overall, DL			2301		2084		2.11 (1.84, 2.38)	100.00
(I^2 = 91.7%, p = 0.000)								

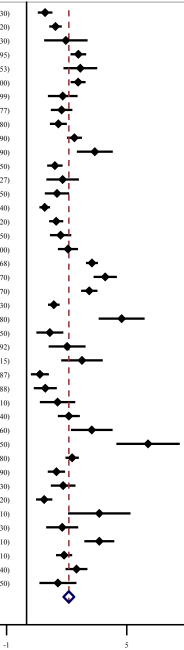

NOTE: Weights are from random-effects model

sFig5 Meta analysis of IOP by follow-up duration before and after XEN surgery

Follow-up and First author (year)	Country	Design	N	Before XEN Mean (SD)	N	After XEN Mean (SD)	IOP (95% CI)	% Weight
Lenzhofer, M.(2019)	Austria	Prospective Study	32	22.20 (4.60)	32	15.00 (6.20)	1.32 (0.78, 1.86)	2.97
Chao, Y.J.(2021)	China	Retrospective Study	37	21.70 (7.70)	37	15.00 (2.00)	1.19 (0.70, 1.69)	3.06
Hengerer, F. H.(2017)	Germany	Retrospective Study	200	31.53 (8.36)	200	14.33 (4.19)	2.60 (2.33, 2.87)	3.44
Hengerer, F. H.(2018)	Germany	Retrospective Study	67	31.85 (8.50)	67	13.99 (2.60)	2.84 (2.36, 3.32)	3.09
Hengerer, F. H.(2019)	Germany	Retrospective Study	103	31.60 (8.90)	103	14.30 (4.20)	2.49 (2.12, 2.85)	3.29
Schargus, M.(2020)	Germany	Retrospective Study	113	22.80 (6.50)	113	15.10 (4.60)	1.37 (1.08, 1.66)	3.40
Cutolo CA.(2020)	Italy	Prospective Study	93	26.40 (8.20)	93	13.40 (6.20)	1.79 (1.45, 2.13)	3.33
Fea, A. M.(2017)	Italy	Prospective Study	21	21.79 (2.80)	21	14.90 (2.10)	2.78 (1.92, 3.64)	2.34
Fea, A. M.(2020)	Italy	Prospective Study	115	25.03 (7.60)	89	15.36 (3.90)	1.54 (1.23, 1.86)	3.37
Post, M.(2020)	Poland	Prospective Study	20	21.56 (2.29)	20	17.69 (2.11)	1.76 (1.02, 2.49)	2.58
Teixeira, F.J.(2021)	Portugal	Prospective Study	12	33.00 (9.00)	12	14.00 (3.00)	2.83 (1.67, 3.99)	1.82
Ibáñez-Muñoz A.(2019)	Spain	Retrospective Study	13	20.85 (3.30)	13	14.38 (4.52)	1.63 (0.74, 2.53)	2.27
Ibáñez-Muñoz, A.(2020)	Spain	Retrospective Study	40	24.00 (3.00)	40	15.00 (1.50)	3.79 (3.06, 4.53)	2.58
Laborda-Guirao T.(2020)	Spain	Retrospective Study	40	21.80 (5.30)	40	14.40 (0.90)	1.95 (1.41, 2.48)	2.99
Marcos Parra, M.T.(2019)	Spain	Retrospective Study	17	22.20 (6.80)	17	15.50 (3.70)	1.22 (0.49, 1.96)	2.58
Urcola, A.(2021)	Spain	Retrospective Study	18	25.20 (7.90)	18	15.10 (3.30)	1.67 (0.90, 2.43)	2.53
Busch, T.(2020)	Sweden	Retrospective Study	113	23.80 (6.20)	113	16.10 (4.70)	1.40 (1.11, 1.69)	3.40
Mansouri, K.(2018)	Switzerland	Prospective Study	57	19.80 (5.83)	57	13.90 (4.60)	1.12 (0.73, 1.52)	3.24
Mansouri, K.(2019)	Switzerland	Prospective Study	40	21.20 (6.80)	40	13.80 (4.10)	1.32 (0.83, 1.80)	3.08
Bravetti, G.E.(2020)	Switzerland	Retrospective Study	60	29.90 (13.20)	47	15.20 (6.60)	1.36 (0.94, 1.78)	3.19
Ozal, S.A.(2017)	Turkey	Retrospective Study	9	36.70 (4.10)	9	17.60 (4.20)	4.60 (2.76, 6.44)	1.03
Sng, C. C.(2018)	UK	Prospective Study	24	30.50 (9.80)	24	12.20 (3.10)	2.52 (1.75, 3.28)	2.52
Karimi, A.(2018)	UK	Retrospective Study	17	21.50 (2.40)	17	13.60 (3.40)	2.68 (1.74, 3.63)	2.19
Linton, E.(2020)	UK	Retrospective Study	151	26.10 (8.30)	149	16.30 (3.70)	1.52 (1.26, 1.78)	3.45
Qureshi, A.(2019)	UK	Retrospective Study	37	36.10 (9.60)	37	12.60 (4.10)	3.18 (2.49, 3.88)	2.67
Smith, M.(2019)	UK	Retrospective Study	68	21.10 (6.40)	68	14.80 (5.10)	1.09 (0.73, 1.45)	3.30
Tan, S. Z.(2018)	UK	Retrospective Study	43	24.90 (7.80)	43	14.50 (3.40)	1.73 (1.23, 2.23)	3.06
Grover, Ds(2017)	US	Prospective Study	65	25.10 (3.70)	65	15.90 (5.20)	2.04 (1.61, 2.46)	3.19
Hong, K.(2020)	US	Prospective Study	28	21.60 (6.60)	28	12.50 (3.60)	1.71 (1.10, 2.33)	2.83
Kalina AG.(2019)	US	Prospective Study	20	24.18 (8.18)	14	13.04 (4.50)	1.61 (0.82, 2.40)	2.48
Sheybani, A.(2016)	US	Prospective Study	27	22.60 (4.11)	27	15.50 (3.65)	1.83 (1.19, 2.47)	2.78
Do, A.(2020)	US	Retrospective Study	76	26.40 (8.40)	29	13.60 (6.30)	1.62 (1.14, 2.11)	3.09
Laroche, D.(2019)	US	Retrospective Study	12	15.30 (6.20)	12	12.90 (4.50)	0.44 (-0.37, 1.25)	2.43
Rather, P.A.(2020)	US	Retrospective Study	73	16.80 (5.40)	73	13.60 (3.50)	0.70 (0.37, 1.04)	3.34
Tan, N.E.(2021)	US	Retrospective Study	50	23.52 (8.53)	36	14.72 (0.79)	1.35 (0.87, 1.82)	3.10
12m								
Subgroup, DL			1911		1803		1.82 (1.59, 2.04)	100.00
(I^2 = 86.5%, p = 0.000)								
Heidinger, A.(2019)	Austria	Retrospective Study	199	22.80 (6.90)	199	17.10 (5.90)	0.89 (0.68, 1.09)	22.09
Arad T.(2019)	Germany	Retrospective Study	10	32.00 (7.50)	10	18.90 (2.60)	2.33 (1.17, 3.50)	15.96
Widder, R. A.(2018)	Germany	Retrospective Study	45	25.20 (7.30)	45	18.80 (8.40)	0.81 (0.38, 1.24)	21.20
Barão, R.C.(2020)	Portugal	Retrospective Study	14	25.00 (10.60)	14	20.90 (11.60)	0.37 (-0.38, 1.12)	19.18
Karimi, A.(2018)	UK	Retrospective Study	186	19.60 (2.00)	71	14.30 (2.10)	2.61 (2.26, 2.97)	21.56
18m								
Subgroup, DL			454		339		1.38 (0.51, 2.24)	100.00
(I^2 = 95.1%, p = 0.000)								
Lenzhofer, M.(2019)	Austria	Prospective Study	69	22.50 (6.50)	69	13.00 (5.15)	1.62 (1.23, 2.01)	9.48
Reitsamer, H.(2019)	Austria	Prospective Study	86	22.10 (4.00)	86	14.40 (3.40)	2.07 (1.70, 2.45)	9.58
Rauchegger, T(2020)	Austria	Retrospective Study	79	23.40 (7.90)	79	14.60 (3.60)	1.43 (1.08, 1.78)	9.72
Scheres, M. J.(2020)	Netherlands	Retrospective Study	26	19.60 (4.90)	16	14.60 (4.60)	1.04 (0.38, 1.71)	7.43
Wałek, E.(2020)	Poland	Prospective Study	9	25.00 (3.00)	9	13.00 (1.00)	5.37 (3.29, 7.44)	1.92
Amljots TS.(2018)	Sweden	Retrospective Study	12	24.90 (7.40)	12	17.00 (5.50)	1.21 (0.34, 2.09)	5.97
Gillmann, K.(2019)	Switzerland	Prospective Study	16	20.50 (4.00)	16	14.90 (2.50)	1.68 (0.87, 2.49)	6.38
Gillmann, K.(2020)	Switzerland	Prospective Study	37	19.00 (6.60)	37	13.70 (4.00)	0.97 (0.49, 1.45)	8.78
Midha, N.(2019)	Switzerland	Prospective Study	40	20.05 (9.35)	40	10.33 (4.41)	1.33 (0.84, 1.82)	8.76
Midha, N.(2020)	Switzerland	Prospective Study	13	23.00 (7.40)	13	13.40 (4.60)	1.56 (0.67, 2.44)	5.91
Başer, E. F.(2021)	Turkey	Retrospective Study	29	24.55 (8.70)	29	15.03 (3.70)	1.42 (0.85, 2.00)	8.06
Olgun, A.(2020)	Turkey	Retrospective Study	51	24.40 (4.30)	51	14.20 (2.20)	2.99 (2.42, 3.55)	8.14
Gabbay, I. E.(2020)	UK	Prospective Study	94	22.90 (6.50)	94	15.00 (3.30)	1.53 (1.21, 1.86)	9.88
24m								
Subgroup, DL			561		551		1.65 (1.34, 1.97)	100.00
(I^2 = 77.9%, p = 0.000)								
Fernández-García, A.(2020)	Spain	Retrospective Study	40	18.02 (5.23)	40	14.62 (1.90)	0.86 (0.41, 1.32)	37.41
Fernández-García, A.(2020)	Spain	Retrospective Study	93	18.23 (5.00)	24	14.63 (1.91)	0.79 (0.33, 1.25)	37.27
Gillmann, K.(2020)	Switzerland	Prospective Study	26	21.00 (7.40)	26	12.90 (2.90)	1.44 (0.83, 2.05)	25.32
36m								
Subgroup, DL			159		90		0.98 (0.62, 1.34)	100.00
(I^2 = 34.6%, p = 0.217)								
Lenzhofer, M.(2019)	Austria	Prospective Study	64	22.50 (4.20)	64	13.40 (3.10)	2.47 (2.00, 2.93)	75.84
Teus, M.A.(2019)	Spain	Retrospective Study	10	19.00 (4.60)	10	10.20 (5.20)	1.79 (0.74, 2.85)	24.16
48m								
Subgroup, DL			74		74		2.30 (1.74, 2.87)	100.00
(I^2 = 23.9%, p = 0.252)								
Ilveskoski, L.(2017)	Finland	Prospective Study	10	21.10 (7.30)	10	10.20 (3.60)	1.89 (0.82, 2.97)	11.00
Buffault, J.(2020)	France	Retrospective Study	77	20.60 (6.60)	77	14.80 (5.90)	0.93 (0.59, 1.26)	16.14
Theillac, V.(2020)	France	Retrospective Study	47	20.80 (6.80)	47	16.20 (2.80)	0.88 (0.46, 1.31)	15.65
Dar, N.(2020)	Israel	Retrospective Study	23	22.60 (7.00)	23	16.70 (6.00)	0.91 (0.30, 1.51)	14.46
Oddone, F.(2020)	Italy	Prospective Study	68	22.70 (4.30)	68	12.40 (2.80)	2.84 (2.36, 3.32)	15.33
Marques, R. E.(2019)	Portugal	Retrospective Study	29	25.52 (8.11)	29	15.00 (7.27)	1.37 (0.79, 1.94)	14.70
Hu, J. Y.(2020)	Singapore	Retrospective Study	19	24.00 (4.00)	11	18.00 (4.00)	1.50 (0.66, 2.34)	12.73
6m								
Subgroup, DL			273		265		1.45 (0.85, 2.05)	100.00
(I^2 = 88.3%, p = 0.000)								
Heterogeneity between groups: p = 0.001								
Overall, DL			3432		3122		1.69 (1.52, 1.86)	
(I^2 = 87.4%, p = 0.000)								

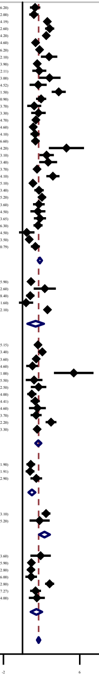

NOTE: Weights and between-subgroup heterogeneity test are from random-effects model

sFig6 Meta analysis of NOAM by follow-up duration before and after XEN surgery

Follow-up and First author (year)	Country	Design	N (Before XEN)	Before XEN Mean (SD)	N (After XEN)	After XEN Mean (SD)	Medication (95% CI)	% Weight
Chao, Y.J.(2021)	China	Retrospective Study	37	3.40 (0.90)	37	1.30 (1.50)	1.70 (1.16, 2.23)	4.12
Hengerer, F. H.(2017)	Germany	Retrospective Study	200	3.09 (1.00)	200	0.30 (0.68)	3.26 (2.96, 3.56)	4.46
Hengerer, F. H.(2018)	Germany	Retrospective Study	67	3.25 (0.80)	67	0.30 (0.70)	3.92 (3.34, 4.51)	4.04
Hengerer, F. H.(2019)	Germany	Retrospective Study	103	3.00 (1.00)	103	0.30 (0.70)	3.13 (2.72, 3.54)	4.32
Schargus, M.(2020)	Germany	Retrospective Study	113	2.50 (1.20)	113	0.80 (1.30)	1.36 (1.07, 1.65)	4.47
Cutolo CA.(2020)	Italy	Prospective Study	93	2.90 (1.00)	93	0.38 (0.95)	2.58 (2.19, 2.97)	4.35
Fea, A. M.(2017)	Italy	Prospective Study	21	2.92 (1.16)	21	0.50 (0.53)	2.68 (1.84, 3.53)	3.53
Fea, A. M.(2020)	Italy	Prospective Study	115	3.25 (1.10)	89	0.53 (1.00)	2.57 (2.20, 2.95)	4.37
Post, M.(2020)	Poland	Prospective Study	20	3.20 (0.77)	20	1.60 (0.99)	1.80 (1.06, 2.54)	3.73
Ibáñez-Muñoz A.(2019)	Spain	Retrospective Study	13	3.00 (1.10)	13	1.30 (1.10)	1.55 (0.66, 2.43)	3.45
Laborda-Guirao T.(2020)	Spain	Retrospective Study	40	2.90 (0.62)	40	1.80 (0.40)	2.11 (1.56, 2.66)	4.09
Marcos Parra, M.T.(2019)	Spain	Retrospective Study	17	2.50 (0.80)	17	0.20 (0.60)	3.25 (2.21, 4.30)	3.13
Urcola, A.(2021)	Spain	Retrospective Study	18	2.90 (0.30)	18	0.40 (0.50)	6.06 (4.48, 7.65)	2.20
Busch, T.(2020)	Sweden	Retrospective Study	113	3.40 (1.00)	113	1.34 (0.80)	2.27 (1.94, 2.61)	4.42
Mansouri, K.(2018)	Switzerland	Prospective Study	57	1.98 (1.16)	57	0.40 (0.80)	1.59 (1.16, 2.01)	4.30
Bravetti, G.E.(2020)	Switzerland	Retrospective Study	60	3.00 (1.30)	47	1.30 (0.90)	1.49 (1.06, 1.92)	4.29
Ozal, S.A.(2017)	Turkey	Retrospective Study	9	3.70 (0.50)	9	0.60 (1.10)	3.63 (2.07, 5.19)	2.23
Sng, C. C.(2018)	UK	Prospective Study	24	3.30 (0.80)	24	0.40 (0.90)	3.41 (2.51, 4.30)	3.42
Karimi, A.(2018)	UK	Retrospective Study	17	2.80 (0.60)	17	1.00 (1.30)	1.78 (0.98, 2.58)	3.61
Qureshi, A.(2019)	UK	Retrospective Study	37	3.69 (0.47)	37	0.62 (1.10)	3.63 (2.88, 4.38)	3.72
Smith, M.(2019)	UK	Retrospective Study	68	2.90 (0.80)	68	1.10 (1.10)	1.87 (1.47, 2.28)	4.33
Grover, Ds(2017)	US	Prospective Study	65	3.50 (1.00)	65	1.70 (1.50)	1.41 (1.03, 1.80)	4.36
Kalina AG.(2019)	US	Prospective Study	20	2.96 (1.20)	14	0.75 (1.27)	1.80 (0.99, 2.61)	3.59
Sheybani, A.(2016)	US	Prospective Study	27	2.70 (1.00)	27	1.50 (0.50)	1.52 (0.91, 2.13)	3.99
Do, A.(2020)	US	Retrospective Study	76	3.60 (0.94)	29	0.90 (1.40)	2.49 (1.94, 3.04)	4.10
Laroche, D.(2019)	US	Retrospective Study	12	3.58 (0.70)	12	1.75 (1.50)	1.56 (0.64, 2.49)	3.37
12m								
Subgroup, DL			1442		1350		2.38 (2.06, 2.71)	100.00
(I^2 = 89.9%, p = 0.000)								
Heidinger, A.(2019)	Austria	Retrospective Study	119	2.90 (1.00)	119	1.80 (1.40)	0.90 (0.64, 1.17)	58.12
Barão, R.C.(2020)	Portugal	Retrospective Study	14	2.93 (0.92)	14	1.07 (0.92)	2.02 (1.10, 2.94)	41.88
18m								
Subgroup, DL			133		133		1.37 (0.29, 2.45)	100.00
(I^2 = 80.8%, p = 0.023)								
Reitsamer, H.(2019)	Austria	Prospective Study	114	2.70 (0.90)	86	1.20 (1.20)	1.44 (1.13, 1.76)	16.19
Rauchegger, T(2020)	Austria	Retrospective Study	79	2.70 (1.10)	79	1.00 (1.20)	1.48 (1.12, 1.83)	15.75
Scheres, M. J.（2020）	Netherlands	Retrospective Study	26	2.70 (1.30)	16	1.10 (1.50)	1.16 (0.49, 1.83)	11.69
Gillmann, K.(2020)	Switzerland	Prospective Study	37	2.16 (1.14)	37	0.46 (0.77)	1.75 (1.21, 2.29)	13.41
Başer, E. F.(2021)	Turkey	Retrospective Study	29	2.83 (0.80)	29	0.86 (1.30)	1.83 (1.21, 2.44)	12.40
Olgun, A.(2020)	Turkey	Retrospective Study	51	3.40 (0.50)	51	2.00 (2.20)	0.88 (0.47, 1.28)	15.09
Gabbay, I. E.(2020)	UK	Prospective Study	94	2.80 (1.10)	94	0.40 (0.90)	2.39 (2.01, 2.76)	15.48
24m								
Subgroup, DL			430		392		1.56 (1.18, 1.95)	100.00
(I^2 = 81.7%, p = 0.000)								
Fernández-García, A.(2020)	Spain	Retrospective Study	40	1.60 (0.94)	40	1.00 (0.87)	0.66 (0.21, 1.11)	62.04
Fernández-García, A.(2020)	Spain	Retrospective Study	93	1.87 (0.94)	14	1.00 (0.88)	0.93 (0.36, 1.51)	37.96
36m								
Subgroup, DL			133		54		0.77 (0.41, 1.12)	100.00
(I^2 = 0.0%, p = 0.469)								
Lenzhofer, M.(2019)	Austria	Prospective Study	64	2.40 (1.30)	64	1.20 (1.30)	0.92 (0.56, 1.29)	100.00
48m								
Subgroup, DL			64		64		0.92 (0.56, 1.29)	100.00
(I^2 = 0.0%, p = .)								
Ilveskoski, L.(2017)	Finland	Prospective Study	10	2.40 (1.10)	10	0.82 (0.30)	1.96 (0.87, 3.05)	23.80
Theillac, V.(2020)	France	Retrospective Study	47	2.66 (1.10)	47	0.49 (1.00)	2.06 (1.56, 2.57)	28.74
Dar, N.(2020)	Israel	Retrospective Study	23	3.58 (0.53)	23	0.36 (0.80)	4.75 (3.60, 5.89)	23.20
Hu, J. Y.(2020)	Singapore	Retrospective Study	19	4.00 (1.00)	11	1.08 (1.15)	2.77 (1.73, 3.80)	24.26
6m								
Subgroup, DL			99		91		2.83 (1.70, 3.96)	100.00
(I^2 = 84.0%, p = 0.000)								
Heterogeneity between groups: p = 0.000								
Overall, DL			2301		2084		2.11 (1.84, 2.38)	
(I^2 = 91.7%, p = 0.000)								

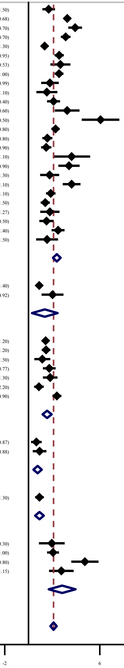

NOTE: Weights and between-subgroup heterogeneity test are from random-effects model

sFig7 Meta analysis of IOP by ethnicity before and after XEN surgery

Continents and First author (year)	Country	Design	N (Before XEN)	Before XEN Mean (SD)	N (After XEN)	After XEN Mean (SD)	IOP (95% CI)	% Weight
Chao, Y.J.(2021)	China	Retrospective Study	37	21.70 (7.70)	37	15.00 (2.00)	1.19 (0.70, 1.69)	18.82
Dar, N.(2020)	Israel	Retrospective Study	23	22.60 (7.00)	23	16.70 (6.00)	0.91 (0.30, 1.51)	18.15
Hu, J. Y.(2020)	Singapore	Retrospective Study	19	24.00 (4.00)	11	18.00 (4.00)	1.50 (0.66, 2.34)	16.56
Başer, E. F.(2021)	Turkey	Retrospective Study	29	24.55 (8.70)	29	15.03 (3.70)	1.42 (0.85, 2.00)	18.34
Olgun, A.(2020)	Turkey	Retrospective Study	51	24.40 (4.30)	51	14.20 (2.20)	2.99 (2.42, 3.55)	18.41
Ozal, S.A.(2017)	Turkey	Retrospective Study	9	36.70 (4.10)	9	17.60 (4.20)	4.60 (2.76, 6.44)	9.72
Asia								
Subgroup, DL			168		160		1.89 (1.10, 2.69)	100.00
(I^2 = 87.8%, p = 0.000)								
Lenzhofer, M.(2019)	Austria	Prospective Study	64	22.50 (4.20)	64	13.40 (3.10)	2.47 (2.00, 2.93)	2.13
Lenzhofer, M.(2019)	Austria	Prospective Study	69	22.50 (6.50)	69	13.00 (5.15)	1.62 (1.23, 2.01)	2.22
Lenzhofer, M.(2019)	Austria	Prospective Study	32	22.20 (4.60)	32	15.00 (6.20)	1.32 (0.78, 1.86)	2.04
Reitsamer, H.(2019)	Austria	Prospective Study	86	22.10 (4.00)	86	14.40 (3.40)	2.07 (1.70, 2.45)	2.23
Heidinger, A.(2019)	Austria	Retrospective Study	199	22.80 (6.90)	199	17.10 (5.90)	0.89 (0.68, 1.09)	2.37
Rauchegger, T(2020)	Austria	Retrospective Study	79	23.40 (7.90)	79	14.60 (3.60)	1.43 (1.08, 1.78)	2.25
Ilveskoski, L.(2017)	Finland	Prospective Study	10	21.10 (7.30)	10	10.20 (3.60)	1.89 (0.82, 2.97)	1.38
Buffault, J.(2020)	France	Retrospective Study	77	20.60 (6.60)	77	14.80 (5.90)	0.93 (0.59, 1.26)	2.27
Theillac, V.(2020)	France	Retrospective Study	47	20.80 (6.80)	47	16.20 (2.80)	0.88 (0.46, 1.31)	2.18
Arad T.(2019)	Germany	Retrospective Study	10	32.00 (7.50)	10	18.90 (2.60)	2.33 (1.17, 3.50)	1.28
Hengerer, F. H.(2017)	Germany	Retrospective Study	200	31.53 (8.36)	200	14.33 (4.19)	2.60 (2.33, 2.87)	2.33
Hengerer, F. H.(2018)	Germany	Retrospective Study	67	31.85 (8.50)	67	13.99 (2.60)	2.84 (2.36, 3.32)	2.11
Hengerer, F. H.(2019)	Germany	Retrospective Study	103	31.60 (8.90)	103	14.30 (4.20)	2.49 (2.12, 2.85)	2.24
Schargus, M.(2020)	Germany	Retrospective Study	113	22.80 (6.50)	113	15.10 (4.60)	1.37 (1.08, 1.66)	2.31
Widder, R. A.(2018)	Germany	Retrospective Study	45	25.20 (7.30)	45	18.80 (8.40)	0.81 (0.38, 1.24)	2.17
Cutolo CA.(2020)	Italy	Prospective Study	93	26.40 (8.20)	93	13.40 (6.20)	1.79 (1.45, 2.13)	2.26
Fea, A. M.(2017)	Italy	Prospective Study	21	21.79 (2.80)	21	14.90 (2.10)	2.78 (1.92, 3.64)	1.63
Fea, A. M.(2020)	Italy	Prospective Study	115	25.03 (7.60)	89	15.36 (3.90)	1.54 (1.23, 1.86)	2.29
Oddone, F.(2020)	Italy	Prospective Study	68	22.70 (4.30)	68	12.40 (2.80)	2.84 (2.36, 3.32)	2.12
Scheres, M. J.(2020)	Netherlands	Retrospective Study	26	19.60 (4.90)	16	14.60 (4.60)	1.04 (0.38, 1.71)	1.88
Post, M.(2020)	Poland	Prospective Study	20	21.56 (2.29)	20	17.69 (2.11)	1.76 (1.02, 2.49)	1.79
Wałek, E.(2020)	Poland	Prospective Study	9	25.00 (3.00)	9	13.00 (1.00)	5.37 (3.29, 7.44)	0.63
Teixeira, F.J.(2021)	Portugal	Prospective Study	12	33.00 (9.00)	12	14.00 (3.00)	2.83 (1.67, 3.99)	1.29
Barão, R.C.(2020)	Portugal	Retrospective Study	14	25.00 (10.60)	14	20.90 (11.60)	0.37 (-0.38, 1.12)	1.78
Marques, R. E.(2019)	Portugal	Retrospective Study	29	25.52 (8.11)	29	15.00 (7.27)	1.37 (0.79, 1.94)	2.00
Fernández-García, A.(2020)	Spain	Retrospective Study	40	18.02 (5.23)	40	14.62 (1.90)	0.86 (0.41, 1.32)	2.14
Fernández-García, A.(2020)	Spain	Retrospective Study	93	18.23 (5.00)	24	14.63 (1.91)	0.79 (0.33, 1.25)	2.14
Ibáñez-Muñoz A.(2019)	Spain	Retrospective Study	13	20.85 (3.30)	13	14.38 (4.52)	1.63 (0.74, 2.53)	1.59
Ibáñez-Muñoz, A.(2020)	Spain	Retrospective Study	40	24.00 (3.00)	40	15.00 (1.50)	3.79 (3.06, 4.53)	1.79
Laborda-Guirao T.(2020)	Spain	Retrospective Study	40	21.80 (5.30)	40	14.40 (0.90)	1.95 (1.41, 2.48)	2.05
Marcos Parra, M.T.(2019)	Spain	Retrospective Study	17	22.20 (6.80)	17	15.50 (3.70)	1.22 (0.49, 1.96)	1.79
Teus, M.A.(2019)	Spain	Retrospective Study	10	19.00 (4.60)	10	10.20 (5.20)	1.79 (0.74, 2.85)	1.40
Urcola, A.(2021)	Spain	Retrospective Study	18	25.20 (7.90)	18	15.10 (3.30)	1.67 (0.90, 2.43)	1.75
Arnljots TS.(2018)	Sweden	Retrospective Study	12	24.90 (7.40)	12	17.00 (5.50)	1.21 (0.34, 2.09)	1.61
Busch, T.(2020)	Sweden	Retrospective Study	113	23.80 (6.20)	113	16.10 (4.70)	1.40 (1.11, 1.69)	2.31
Gillmann, K.(2019)	Switzerland	Prospective Study	16	20.50 (4.00)	16	14.90 (2.50)	1.68 (0.87, 2.49)	1.69
Gillmann, K.(2020)	Switzerland	Prospective Study	26	21.00 (7.40)	26	12.90 (2.90)	1.44 (0.83, 2.05)	1.95
Gillmann, K.(2020)	Switzerland	Prospective Study	37	19.00 (6.60)	37	13.70 (4.00)	0.97 (0.49, 1.45)	2.11
Mansouri, K.(2018)	Switzerland	Prospective Study	57	19.80 (5.83)	57	13.90 (4.60)	1.12 (0.73, 1.52)	2.21
Mansouri, K.(2019)	Switzerland	Prospective Study	40	21.20 (6.80)	40	13.80 (4.10)	1.32 (0.83, 1.80)	2.11
Midha, N.(2019)	Switzerland	Prospective Study	40	20.05 (9.35)	40	10.33 (4.41)	1.33 (0.84, 1.82)	2.11
Midha, N.(2020)	Switzerland	Prospective Study	13	23.00 (7.40)	13	13.40 (4.60)	1.56 (0.67, 2.44)	1.60
Bravetti, G.E.(2020)	Switzerland	Retrospective Study	60	29.90 (13.20)	47	15.20 (6.60)	1.36 (0.94, 1.78)	2.18
Gabbay, I. E.(2020)	UK	Prospective Study	94	22.90 (6.50)	94	15.00 (3.30)	1.53 (1.21, 1.86)	2.28
Sng, C. C.(2018)	UK	Prospective Study	24	30.50 (9.80)	24	12.20 (3.10)	2.52 (1.75, 3.28)	1.75
Karimi, A.(2018)	UK	Retrospective Study	17	21.50 (2.40)	17	13.60 (3.40)	2.68 (1.74, 3.63)	1.53
Karimi, A.(2018)	UK	Retrospective Study	186	19.60 (2.00)	71	14.30 (2.10)	2.61 (2.26, 2.97)	2.25
Linton, E.(2020)	UK	Retrospective Study	151	26.10 (8.30)	149	16.30 (3.70)	1.52 (1.26, 1.78)	2.33
Qureshi, A.(2019)	UK	Retrospective Study	37	36.10 (9.60)	37	12.60 (4.10)	3.18 (2.49, 3.88)	1.85
Smith, M.(2019)	UK	Retrospective Study	68	21.10 (6.40)	68	14.80 (5.10)	1.09 (0.73, 1.45)	2.24
Tan, S. Z.(2018)	UK	Retrospective Study	43	24.90 (7.80)	43	14.50 (3.40)	1.73 (1.23, 2.23)	2.09
Europe								
Subgroup, DL			2913		2678		1.72 (1.53, 1.91)	100.00
(I^2 = 88.3%, p = 0.000)								
Grover, Ds(2017)	US	Prospective Study	65	25.10 (3.70)	65	15.90 (5.20)	2.04 (1.61, 2.46)	14.00
Hong, K.(2020)	US	Prospective Study	28	21.60 (6.60)	28	12.50 (3.60)	1.71 (1.10, 2.33)	12.05
Kalina AG.(2019)	US	Prospective Study	20	24.18 (8.18)	14	13.04 (4.50)	1.61 (0.82, 2.40)	10.29
Sheybani, A.(2016)	US	Prospective Study	27	22.60 (4.11)	27	15.50 (3.65)	1.83 (1.19, 2.47)	11.81
Do, A.(2020)	US	Retrospective Study	76	26.40 (8.40)	29	13.60 (6.30)	1.62 (1.14, 2.11)	13.43
Laroche, D.(2019)	US	Retrospective Study	12	15.30 (6.20)	12	12.90 (4.50)	0.44 (-0.37, 1.25)	10.08
Rather, P.A.(2020)	US	Retrospective Study	73	16.80 (5.40)	73	13.60 (3.50)	0.70 (0.37, 1.04)	14.83
Tan, N.E.(2021)	US	Retrospective Study	50	23.52 (8.53)	36	14.72 (0.79)	1.35 (0.87, 1.82)	13.51
North America								
Subgroup, DL			351		284		1.42 (1.01, 1.84)	100.00
(I^2 = 79.6%, p = 0.000)								
Heterogeneity between groups: p = 0.386								
Overall, DL			3432		3122		1.69 (1.52, 1.86)	
(I^2 = 87.4%, p = 0.000)								

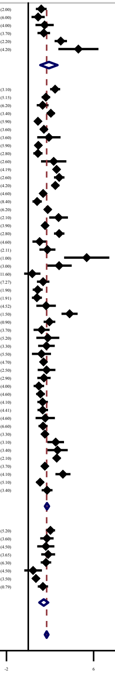

NOTE: Weights and between-subgroup heterogeneity test are from random-effects model

sFig8 Meta analysis of IOP by developed and developing country and after XEN surgery

Country (Developed) and First author (year)	Country	Design	N	Before XEN Mean (SD)	N	After XEN Mean (SD)	IOP (95% CI)	% Weight
Lenzhofer, M.(2019)	Austria	Prospective Study	64	22.50 (4.20)	64	13.40 (3.10)	2.47 (2.00, 2.93)	1.79
Lenzhofer, M.(2019)	Austria	Prospective Study	69	22.50 (6.50)	69	13.00 (5.15)	1.62 (1.23, 2.01)	1.86
Lenzhofer, M.(2019)	Austria	Prospective Study	32	22.20 (4.60)	32	15.00 (6.20)	1.32 (0.78, 1.86)	1.70
Reitsamer, H.(2019)	Austria	Prospective Study	86	22.10 (4.00)	86	14.40 (3.40)	2.07 (1.70, 2.45)	1.87
Heidinger, A.(2019)	Austria	Retrospective Study	199	22.80 (6.90)	199	17.10 (5.90)	0.89 (0.68, 1.09)	2.00
Rauchegger, T(2020)	Austria	Retrospective Study	79	23.40 (7.90)	79	14.60 (3.60)	1.43 (1.08, 1.78)	1.89
Ilveskoski, L.(2017)	Finland	Prospective Study	10	21.10 (7.30)	10	10.20 (3.60)	1.89 (0.82, 2.97)	1.14
Buffault, J.(2020)	France	Retrospective Study	77	20.60 (6.60)	77	14.80 (5.90)	0.93 (0.59, 1.26)	1.91
Theillac, V.(2020)	France	Retrospective Study	47	20.80 (6.80)	47	16.20 (2.80)	0.88 (0.46, 1.31)	1.83
Arad T.(2019)	Germany	Retrospective Study	10	32.00 (7.50)	10	18.90 (2.60)	2.33 (1.17, 3.50)	1.06
Hengerer, F. H.(2017)	Germany	Retrospective Study	200	31.53 (8.36)	200	14.33 (4.19)	2.60 (2.33, 2.87)	1.96
Hengerer, F. H.(2018)	Germany	Retrospective Study	67	31.85 (8.50)	67	13.99 (2.60)	2.84 (2.36, 3.32)	1.77
Hengerer, F. H.(2019)	Germany	Retrospective Study	103	31.60 (8.90)	103	14.30 (4.20)	2.49 (2.12, 2.85)	1.88
Schargus, M.(2020)	Germany	Retrospective Study	113	22.80 (6.50)	113	15.10 (4.60)	1.37 (1.08, 1.66)	1.94
Widder, R. A.(2018)	Germany	Retrospective Study	45	25.20 (7.30)	45	18.80 (8.40)	0.81 (0.38, 1.24)	1.82
Dar, N.(2020)	Israel	Retrospective Study	23	22.60 (7.00)	23	16.70 (6.00)	0.91 (0.30, 1.51)	1.63
Cutolo CA.(2020)	Italy	Prospective Study	93	26.40 (8.20)	93	13.40 (6.20)	1.79 (1.45, 2.13)	1.90
Fea, A. M.(2017)	Italy	Prospective Study	21	21.79 (2.80)	21	14.90 (2.10)	2.78 (1.92, 3.64)	1.36
Fea, A. M.(2020)	Italy	Prospective Study	115	25.03 (7.60)	89	15.36 (3.90)	1.54 (1.23, 1.86)	1.92
Oddone, F.(2020)	Italy	Prospective Study	68	22.70 (4.30)	68	12.40 (2.80)	2.84 (2.36, 3.32)	1.77
Scheres, M. J.(2020)	Netherlands	Retrospective Study	26	19.60 (4.90)	16	14.60 (4.60)	1.04 (0.38, 1.71)	1.57
Post, M.(2020)	Poland	Prospective Study	20	21.56 (2.29)	20	17.69 (2.11)	1.76 (1.02, 2.49)	1.49
Walek, E.(2020)	Poland	Prospective Study	9	25.00 (3.00)	9	13.00 (1.00)	5.37 (3.29, 7.44)	0.51
Teixeira, F.J.(2021)	Portugal	Prospective Study	12	33.00 (9.00)	12	14.00 (3.00)	2.83 (1.67, 3.99)	1.06
Barão, R.C.(2020)	Portugal	Retrospective Study	14	25.00 (10.60)	14	20.90 (11.60)	0.37 (-0.38, 1.12)	1.48
Marques, R. E.(2019)	Portugal	Retrospective Study	29	25.52 (8.11)	29	15.00 (7.27)	1.37 (0.79, 1.94)	1.67
Hu, J. Y.(2020)	Singapore	Retrospective Study	19	24.00 (4.00)	11	18.00 (4.00)	1.50 (0.66, 2.34)	1.38
Fernández-García, A.(2020)	Spain	Retrospective Study	40	18.02 (5.23)	40	14.62 (1.90)	0.86 (0.41, 1.32)	1.79
Fernández-García, A.(2020)	Spain	Retrospective Study	93	18.23 (5.00)	24	14.63 (1.91)	0.79 (0.33, 1.25)	1.79
Ibáñez-Muñoz A.(2019)	Spain	Retrospective Study	13	20.85 (3.30)	13	14.38 (4.52)	1.63 (0.74, 2.53)	1.32
Ibáñez-Muñoz, A.(2020)	Spain	Retrospective Study	40	24.00 (3.00)	40	15.00 (1.50)	3.79 (3.06, 4.53)	1.49
Laborda-Guirao T.(2020)	Spain	Retrospective Study	40	21.80 (5.30)	40	14.40 (0.90)	1.95 (1.41, 2.48)	1.71
Marcos Parra, M.T.(2019)	Spain	Retrospective Study	17	22.20 (6.80)	17	15.50 (3.70)	1.22 (0.49, 1.96)	1.49
Teus, M.A.(2019)	Spain	Retrospective Study	10	19.00 (4.60)	10	10.20 (5.20)	1.79 (0.74, 2.85)	1.16
Urcola, A.(2021)	Spain	Retrospective Study	18	25.20 (7.90)	18	15.10 (3.30)	1.67 (0.90, 2.43)	1.46
Arnljots TS.(2018)	Sweden	Retrospective Study	12	24.90 (7.40)	12	17.00 (5.50)	1.21 (0.34, 2.09)	1.34
Busch, T.(2020)	Sweden	Retrospective Study	113	23.80 (6.20)	113	16.10 (4.70)	1.40 (1.11, 1.69)	1.94
Gillmann, K.(2019)	Switzerland	Prospective Study	16	20.50 (4.00)	16	14.90 (2.50)	1.68 (0.87, 2.49)	1.41
Gillmann, K.(2020)	Switzerland	Prospective Study	26	21.00 (7.40)	26	12.90 (2.90)	1.44 (0.83, 2.05)	1.63
Gillmann, K.(2020)	Switzerland	Prospective Study	37	19.00 (6.60)	37	13.70 (4.00)	0.97 (0.49, 1.45)	1.77
Mansouri, K.(2018)	Switzerland	Prospective Study	57	19.80 (5.83)	57	13.90 (4.60)	1.12 (0.73, 1.52)	1.85
Mansouri, K.(2019)	Switzerland	Prospective Study	40	21.20 (6.80)	40	13.80 (4.10)	1.32 (0.83, 1.80)	1.77
Midha, N.(2019)	Switzerland	Prospective Study	40	20.05 (9.35)	40	10.33 (4.41)	1.33 (0.84, 1.82)	1.76
Midha, N.(2020)	Switzerland	Prospective Study	13	23.00 (7.40)	13	13.40 (4.60)	1.56 (0.67, 2.44)	1.33
Bravetti, G.E.(2020)	Switzerland	Retrospective Study	60	29.90 (13.20)	47	15.20 (6.60)	1.36 (0.94, 1.78)	1.83
Gabbay, I. E.(2020)	UK	Prospective Study	94	22.90 (6.50)	94	15.00 (3.30)	1.53 (1.21, 1.86)	1.91
Sng, C. C.(2018)	UK	Prospective Study	24	30.50 (9.80)	24	12.20 (3.10)	2.52 (1.75, 3.28)	1.46
Karimi, A.(2018)	UK	Retrospective Study	17	21.50 (2.40)	17	13.60 (3.40)	2.68 (1.74, 3.63)	1.27
Karimi, A.(2018)	UK	Retrospective Study	186	19.60 (2.00)	71	14.30 (2.10)	2.61 (2.26, 2.97)	1.89
Linton, E.(2020)	UK	Retrospective Study	151	26.10 (8.30)	149	16.30 (3.70)	1.52 (1.26, 1.78)	1.96
Qureshi, A.(2019)	UK	Retrospective Study	37	36.10 (9.60)	37	12.60 (4.10)	3.18 (2.49, 3.88)	1.54
Smith, M.(2019)	UK	Retrospective Study	68	21.10 (6.40)	68	14.80 (5.10)	1.09 (0.73, 1.45)	1.88
Tan, S. Z.(2018)	UK	Retrospective Study	43	24.90 (7.80)	43	14.50 (3.40)	1.73 (1.23, 2.23)	1.75
Grover, Ds(2017)	US	Prospective Study	65	25.10 (3.70)	65	15.90 (5.20)	2.04 (1.61, 2.46)	1.82
Hong, K.(2020)	US	Prospective Study	28	21.60 (6.60)	28	12.50 (3.60)	1.71 (1.10, 2.33)	1.63
Kalina AG.(2019)	US	Prospective Study	20	24.18 (8.18)	14	13.04 (4.50)	1.61 (0.82, 2.40)	1.43
Sheybani, A.(2016)	US	Prospective Study	27	22.60 (4.11)	27	15.50 (3.65)	1.83 (1.19, 2.47)	1.60
Do, A.(2020)	US	Retrospective Study	76	26.40 (8.40)	29	13.60 (6.30)	1.62 (1.14, 2.11)	1.77
Laroche, D.(2019)	US	Retrospective Study	12	15.30 (6.20)	12	12.90 (4.50)	0.44 (-0.37, 1.25)	1.41
Rather, P.A.(2020)	US	Retrospective Study	73	16.80 (5.40)	73	13.60 (3.50)	0.70 (0.37, 1.04)	1.91
Tan, N.E.(2021)	US	Retrospective Study	50	23.52 (8.53)	36	14.72 (0.79)	1.35 (0.87, 1.82)	1.78
Developed Country								
Subgroup, DL			3306		2996		1.66 (1.49, 1.83)	100.00
(I^2 = 87.3%, p = 0.000)								
Chao, Y.J.(2021)	China	Retrospective Study	37	21.70 (7.70)	37	15.00 (2.00)	1.19 (0.70, 1.69)	28.18
Başer, E. F.(2021)	Turkey	Retrospective Study	29	24.55 (8.70)	29	15.03 (3.70)	1.42 (0.85, 2.00)	27.63
Olgun, A.(2020)	Turkey	Retrospective Study	51	24.40 (4.30)	51	14.20 (2.20)	2.99 (2.42, 3.55)	27.70
Ozal, S.A.(2017)	Turkey	Retrospective Study	9	36.70 (4.10)	9	17.60 (4.20)	4.60 (2.76, 6.44)	16.49
Developing Country								
Subgroup, DL			126		126		2.32 (1.20, 3.43)	100.00
(I^2 = 90.9%, p = 0.000)								
Heterogeneity between groups: p = 0.256								
Overall, DL			3432		3122		1.69 (1.52, 1.86)	
(I^2 = 87.4%, p = 0.000)								

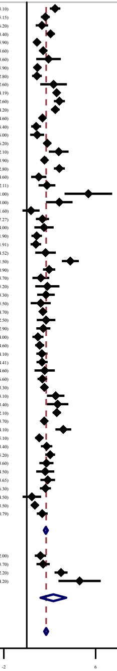

NOTE: Weights and between-subgroup heterogeneity test are from random-effects model

sFig9 Meta analysis of NOAM by developed and developing country before and after XEN surgery

Country (Developed) and First author (year)	Country	Design	Before XEN N	Before XEN Mean (SD)	After XEN N	After XEN Mean (SD)	Medication (95% CI)	% Weight
Lenzhofer, M.(2019)	Austria	Prospective Study	64	2.40 (1.30)	64	1.20 (1.30)	0.92 (0.56, 1.29)	2.91
Reitsamer, H.(2019)	Austria	Prospective Study	114	2.70 (0.90)	86	1.20 (1.20)	1.44 (1.13, 1.76)	2.95
Heidinger, A.(2019)	Austria	Retrospective Study	119	2.90 (1.00)	119	1.80 (1.40)	0.90 (0.64, 1.17)	2.98
Rauchegger, T(2020)	Austria	Retrospective Study	79	2.70 (1.10)	79	1.00 (1.20)	1.48 (1.12, 1.83)	2.92
Ilveskoski, L.(2017)	Finland	Prospective Study	10	2.40 (1.10)	10	0.82 (0.30)	1.96 (0.87, 3.05)	2.11
Theillac, V.(2020)	France	Retrospective Study	47	2.66 (1.10)	47	0.49 (1.00)	2.06 (1.56, 2.57)	2.79
Hengerer, F. H.(2017)	Germany	Retrospective Study	200	3.09 (1.00)	200	0.30 (0.68)	3.26 (2.96, 3.56)	2.96
Hengerer, F. H.(2018)	Germany	Retrospective Study	67	3.25 (0.80)	67	0.30 (0.70)	3.92 (3.34, 4.51)	2.71
Hengerer, F. H.(2019)	Germany	Retrospective Study	103	3.00 (1.00)	103	0.30 (0.70)	3.13 (2.72, 3.54)	2.88
Schargus, M.(2020)	Germany	Retrospective Study	113	2.50 (1.20)	113	0.80 (1.30)	1.36 (1.07, 1.65)	2.96
Dar, N.(2020)	Israel	Retrospective Study	23	3.58 (0.53)	23	0.36 (0.80)	4.75 (3.60, 5.89)	2.04
Cutolo CA.(2020)	Italy	Prospective Study	93	2.90 (1.00)	93	0.38 (0.95)	2.58 (2.19, 2.97)	2.89
Fea, A. M.(2017)	Italy	Prospective Study	21	2.92 (1.16)	21	0.50 (0.53)	2.68 (1.84, 3.53)	2.41
Fea, A. M.(2020)	Italy	Prospective Study	115	3.25 (1.10)	89	0.53 (1.00)	2.57 (2.20, 2.95)	2.90
Scheres, M. J.（2020）	Netherlands	Retrospective Study	26	2.70 (1.30)	16	1.10 (1.50)	1.16 (0.49, 1.83)	2.61
Post, M.(2020)	Poland	Prospective Study	20	3.20 (0.77)	20	1.60 (0.99)	1.80 (1.06, 2.54)	2.53
Barão, R.C.(2020)	Portugal	Retrospective Study	14	2.93 (0.92)	14	1.07 (0.92)	2.02 (1.10, 2.94)	2.31
Hu, J. Y.(2020)	Singapore	Retrospective Study	19	4.00 (1.00)	11	1.08 (1.15)	2.77 (1.73, 3.80)	2.17
Fernández-García, A.(2020)	Spain	Retrospective Study	40	1.60 (0.94)	40	1.00 (0.87)	0.66 (0.21, 1.11)	2.84
Fernández-García, A.(2020)	Spain	Retrospective Study	93	1.87 (0.94)	14	1.00 (0.88)	0.93 (0.36, 1.51)	2.72
Ibáñez-Muñoz A.(2019)	Spain	Retrospective Study	13	3.00 (1.10)	13	1.30 (1.10)	1.55 (0.66, 2.43)	2.36
Laborda-Guirao T.(2020)	Spain	Retrospective Study	40	2.90 (0.62)	40	1.80 (0.40)	2.11 (1.56, 2.66)	2.74
Marcos Parra, M.T.(2019)	Spain	Retrospective Study	17	2.50 (0.80)	17	0.20 (0.60)	3.25 (2.21, 4.30)	2.16
Urcola, A.(2021)	Spain	Retrospective Study	18	2.90 (0.30)	18	0.40 (0.50)	6.06 (4.48, 7.65)	1.57
Busch, T.(2020)	Sweden	Retrospective Study	113	3.40 (1.00)	113	1.34 (0.80)	2.27 (1.94, 2.61)	2.93
Gillmann, K.(2020)	Switzerland	Prospective Study	37	2.16 (1.14)	37	0.46 (0.77)	1.75 (1.21, 2.29)	2.76
Mansouri, K.(2018)	Switzerland	Prospective Study	57	1.98 (1.16)	57	0.40 (0.80)	1.59 (1.16, 2.01)	2.86
Bravetti, G.E.(2020)	Switzerland	Retrospective Study	60	3.00 (1.30)	47	1.30 (0.90)	1.49 (1.06, 1.92)	2.86
Gabbay, I. E.(2020)	UK	Prospective Study	94	2.80 (1.10)	94	0.40 (0.90)	2.39 (2.01, 2.76)	2.90
Sng, C. C.(2018)	UK	Prospective Study	24	3.30 (0.80)	24	0.40 (0.90)	3.41 (2.51, 4.30)	2.34
Karimi, A.(2018)	UK	Retrospective Study	17	2.80 (0.60)	17	1.00 (1.30)	1.78 (0.98, 2.58)	2.46
Qureshi, A.(2019)	UK	Retrospective Study	37	3.69 (0.47)	37	0.62 (1.10)	3.63 (2.88, 4.38)	2.52
Smith, M.(2019)	UK	Retrospective Study	68	2.90 (0.80)	68	1.10 (1.10)	1.87 (1.47, 2.28)	2.88
Grover, Ds(2017)	US	Prospective Study	65	3.50 (1.00)	65	1.70 (1.50)	1.41 (1.03, 1.80)	2.90
Kalina AG.(2019)	US	Prospective Study	20	2.96 (1.20)	14	0.75 (1.27)	1.80 (0.99, 2.61)	2.44
Sheybani, A.(2016)	US	Prospective Study	27	2.70 (1.00)	27	1.50 (0.50)	1.52 (0.91, 2.13)	2.68
Do, A.(2020)	US	Retrospective Study	76	3.60 (0.94)	29	0.90 (1.40)	2.49 (1.94, 3.04)	2.75
Laroche, D.(2019)	US	Retrospective Study	12	3.58 (0.70)	12	1.75 (1.50)	1.56 (0.64, 2.49)	2.31
Developed Country								
Subgroup, DL			2175		1958		2.14 (1.86, 2.42)	100.00
(I^2 = 92.0%, p = 0.000)								
Chao, Y.J.(2021)	China	Retrospective Study	37	3.40 (0.90)	37	1.30 (1.50)	1.70 (1.16, 2.23)	28.55
Başer, E. F.(2021)	Turkey	Retrospective Study	29	2.83 (0.80)	29	0.86 (1.30)	1.83 (1.21, 2.44)	27.27
Olgun, A.(2020)	Turkey	Retrospective Study	51	3.40 (0.50)	51	2.00 (2.20)	0.88 (0.47, 1.28)	30.33
Ozal, S.A.(2017)	Turkey	Retrospective Study	9	3.70 (0.50)	9	0.60 (1.10)	3.63 (2.07, 5.19)	13.84
Developing Country								
Subgroup, DL			126		126		1.75 (0.99, 2.51)	100.00
(I^2 = 82.6%, p = 0.001)								
Heterogeneity between groups: p = 0.348								
Overall, DL			2301		2084		2.11 (1.84, 2.38)	
(I^2 = 91.7%, p = 0.000)								

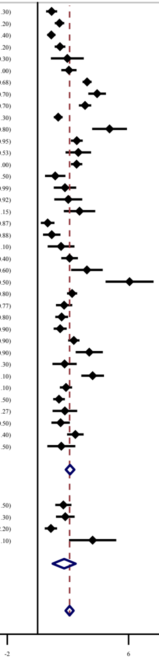

NOTE: Weights and between-subgroup heterogeneity test are from random-effects model

sFig10 Meta analysis of NOAM by ethnicity before after XEN surgery

Continents and First author (year)	Country	Design	N	Before XEN Mean (SD)	N	After XEN Mean (SD)	Medication (95% CI)	% Weight
Chao, Y.J.(2021)	China	Retrospective Study	37	3.40 (0.90)	37	1.30 (1.50)	1.70 (1.16, 2.23)	18.55
Dar, N.(2020)	Israel	Retrospective Study	23	3.58 (0.53)	23	0.36 (0.80)	4.75 (3.60, 5.89)	15.34
Hu, J. Y.(2020)	Singapore	Retrospective Study	19	4.00 (1.00)	11	1.08 (1.15)	2.77 (1.73, 3.80)	15.98
Başer, E. F.(2021)	Turkey	Retrospective Study	29	2.83 (0.80)	29	0.86 (1.30)	1.83 (1.21, 2.44)	18.20
Olgun, A.(2020)	Turkey	Retrospective Study	51	3.40 (0.50)	51	2.00 (2.20)	0.88 (0.47, 1.28)	19.01
Ozal, S.A.(2017)	Turkey	Retrospective Study	9	3.70 (0.50)	9	0.60 (1.10)	3.63 (2.07, 5.19)	12.92
Asia								
Subgroup, DL			168		160		2.45 (1.50, 3.41)	100.00
(I^2 = 90.5%, p = 0.000)								
Lenzhofer, M.(2019)	Austria	Prospective Study	64	2.40 (1.30)	64	1.20 (1.30)	0.92 (0.56, 1.29)	3.51
Reitsamer, H.(2019)	Austria	Prospective Study	114	2.70 (0.90)	86	1.20 (1.20)	1.44 (1.13, 1.76)	3.55
Heidinger, A.(2019)	Austria	Retrospective Study	119	2.90 (1.00)	119	1.80 (1.40)	0.90 (0.64, 1.17)	3.59
Rauchegger, T(2020)	Austria	Retrospective Study	79	2.70 (1.10)	79	1.00 (1.20)	1.48 (1.12, 1.83)	3.52
Ilveskoski, L.(2017)	Finland	Prospective Study	10	2.40 (1.10)	10	0.82 (0.30)	1.96 (0.87, 3.05)	2.57
Theillac, V.(2020)	France	Retrospective Study	47	2.66 (1.10)	47	0.49 (1.00)	2.06 (1.56, 2.57)	3.37
Hengerer, F. H.(2017)	Germany	Retrospective Study	200	3.09 (1.00)	200	0.30 (0.68)	3.26 (2.96, 3.56)	3.56
Hengerer, F. H.(2018)	Germany	Retrospective Study	67	3.25 (0.80)	67	0.30 (0.70)	3.92 (3.34, 4.51)	3.28
Hengerer, F. H.(2019)	Germany	Retrospective Study	103	3.00 (1.00)	103	0.30 (0.70)	3.13 (2.72, 3.54)	3.47
Schargus, M.(2020)	Germany	Retrospective Study	113	2.50 (1.20)	113	0.80 (1.30)	1.36 (1.07, 1.65)	3.57
Cutolo CA.(2020)	Italy	Prospective Study	93	2.90 (1.00)	93	0.38 (0.95)	2.58 (2.19, 2.97)	3.49
Fea, A. M.(2017)	Italy	Prospective Study	21	2.92 (1.16)	21	0.50 (0.53)	2.68 (1.84, 3.53)	2.92
Fea, A. M.(2020)	Italy	Prospective Study	115	3.25 (1.10)	89	0.53 (1.00)	2.57 (2.20, 2.95)	3.50
Scheres, M. J.（2020）	Netherlands	Retrospective Study	26	2.70 (1.30)	16	1.10 (1.50)	1.16 (0.49, 1.83)	3.16
Post, M.(2020)	Poland	Prospective Study	20	3.20 (0.77)	20	1.60 (0.99)	1.80 (1.06, 2.54)	3.07
Barão, R.C.(2020)	Portugal	Retrospective Study	14	2.93 (0.92)	14	1.07 (0.92)	2.02 (1.10, 2.94)	2.81
Fernández-García, A.(2020)	Spain	Retrospective Study	40	1.60 (0.94)	40	1.00 (0.87)	0.66 (0.21, 1.11)	3.43
Fernández-García, A.(2020)	Spain	Retrospective Study	93	1.87 (0.94)	14	1.00 (0.88)	0.93 (0.36, 1.51)	3.28
Ibáñez-Muñoz A.(2019)	Spain	Retrospective Study	13	3.00 (1.10)	13	1.30 (1.10)	1.55 (0.66, 2.43)	2.86
Laborda-Guirao T.(2020)	Spain	Retrospective Study	40	2.90 (0.62)	40	1.80 (0.40)	2.11 (1.56, 2.66)	3.32
Marcos Parra, M.T.(2019)	Spain	Retrospective Study	17	2.50 (0.80)	17	0.20 (0.60)	3.25 (2.21, 4.30)	2.63
Urcola, A.(2021)	Spain	Retrospective Study	18	2.90 (0.30)	18	0.40 (0.50)	6.06 (4.48, 7.65)	1.92
Busch, T.(2020)	Sweden	Retrospective Study	113	3.40 (1.00)	113	1.34 (0.80)	2.27 (1.94, 2.61)	3.54
Gillmann, K.(2020)	Switzerland	Prospective Study	37	2.16 (1.14)	37	0.46 (0.77)	1.75 (1.21, 2.29)	3.33
Mansouri, K.(2018)	Switzerland	Prospective Study	57	1.98 (1.16)	57	0.40 (0.80)	1.59 (1.16, 2.01)	3.46
Bravetti, G.E.(2020)	Switzerland	Retrospective Study	60	3.00 (1.30)	47	1.30 (0.90)	1.49 (1.06, 1.92)	3.45
Gabbay, I. E.(2020)	UK	Prospective Study	94	2.80 (1.10)	94	0.40 (0.90)	2.39 (2.01, 2.76)	3.50
Sng, C. C.(2018)	UK	Prospective Study	24	3.30 (0.80)	24	0.40 (0.90)	3.41 (2.51, 4.30)	2.84
Karimi, A.(2018)	UK	Retrospective Study	17	2.80 (0.60)	17	1.00 (1.30)	1.78 (0.98, 2.58)	2.98
Qureshi, A.(2019)	UK	Retrospective Study	37	3.69 (0.47)	37	0.62 (1.10)	3.63 (2.88, 4.38)	3.06
Smith, M.(2019)	UK	Retrospective Study	68	2.90 (0.80)	68	1.10 (1.10)	1.87 (1.47, 2.28)	3.47
Europe								
Subgroup, DL			1933		1777		2.12 (1.80, 2.44)	100.00
(I^2 = 92.9%, p = 0.000)								
Grover, Ds(2017)	US	Prospective Study	65	3.50 (1.00)	65	1.70 (1.50)	1.41 (1.03, 1.80)	26.95
Kalina AG.(2019)	US	Prospective Study	20	2.96 (1.20)	14	0.75 (1.27)	1.80 (0.99, 2.61)	15.98
Sheybani, A.(2016)	US	Prospective Study	27	2.70 (1.00)	27	1.50 (0.50)	1.52 (0.91, 2.13)	20.78
Do, A.(2020)	US	Retrospective Study	76	3.60 (0.94)	29	0.90 (1.40)	2.49 (1.94, 3.04)	22.43
Laroche, D.(2019)	US	Retrospective Study	12	3.58 (0.70)	12	1.75 (1.50)	1.56 (0.64, 2.49)	13.85
North America								
Subgroup, DL			200		147		1.76 (1.31, 2.21)	100.00
(I^2 = 62.4%, p = 0.031)								
Heterogeneity between groups: p = 0.290								
Overall, DL			2301		2084		2.11 (1.84, 2.38)	
(I^2 = 91.7%, p = 0.000)								

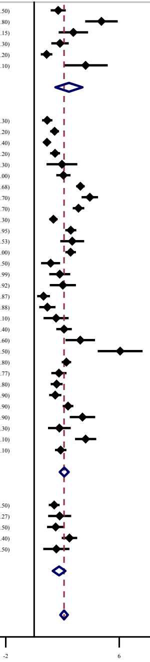

NOTE: Weights and between-subgroup heterogeneity test are from random-effects model

sFig11Meta analysis of IOP before XEN surgery betweem POAG and PEXG

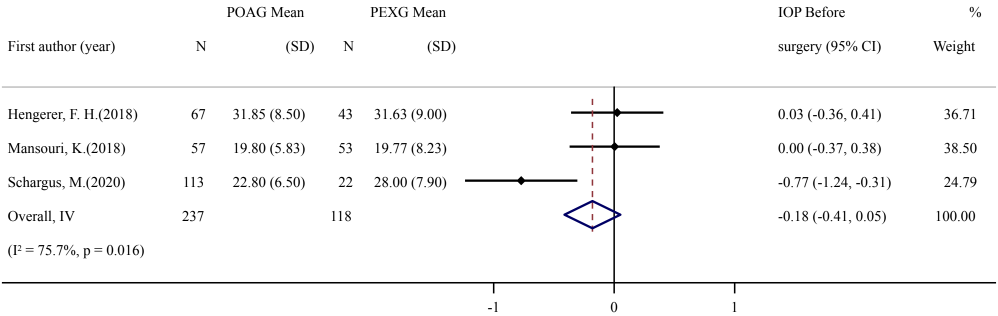

sFig12 Meta analysis of IOP after XEN surgery betweem POAG and PEXG

First author (year)	N	POAG Mean (SD)	N	PEXG Mean (SD)	IOP After surgery (95% CI)	% Weight
Hengerer, F. H.(2018)	67	13.99 (2.60)	43	13.28 (3.10)	0.25 (-0.13, 0.64)	34.77
Mansouri, K.(2018)	57	13.90 (4.60)	53	13.60 (4.30)	0.07 (-0.31, 0.44)	35.53
Schargus, M.(2020)	113	15.10 (4.60)	22	17.10 (6.60)	-0.40 (-0.86, 0.06)	29.70
Overall, DL	237		118		-0.01 (-0.37, 0.35)	100.00
(I^2 = 57.6%, p = 0.094)						

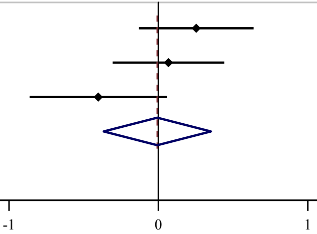

NOTE: Weights are from random-effects model

sFig13 Meta analysis of NOAM before XEN surgery betweem POAG and PEXG

First author (year)	N	POAG Mean (SD)	N	PEXG Mean (SD)	Medication Before surgery (95% CI)	% Weight
Hengerer, F. H.(2018)	67	3.25 (0.80)	43	3.05 (1.00)	0.23 (-0.16, 0.61)	36.27
Mansouri, K.(2018)	57	1.98 (1.16)	53	2.02 (1.34)	-0.03 (-0.41, 0.34)	38.26
Schargus, M.(2020)	113	2.50 (1.20)	22	2.90 (1.20)	-0.33 (-0.79, 0.13)	25.46
Overall, IV	237		118		-0.01 (-0.25, 0.22)	100.00
($I^2 = 40.8\%$, $p = 0.185$)						

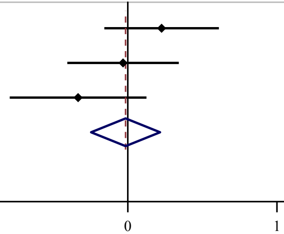

sFig14 Meta analysis of NOAM after XEN surgery betweem POAG and PEXG

First author (year)	N	POAG Mean (SD)	N	PEXG Mean (SD)	Medication After surgery (95% CI)	% Weight
Hengerer, F. H.(2018)	67	0.30 (0.70)	43	0.30 (0.60)	0.00 (-0.38, 0.38)	36.38
Mansouri, K.(2018)	57	0.40 (0.80)	53	0.50 (0.80)	-0.12 (-0.50, 0.25)	38.08
Schargus, M.(2020)	113	0.80 (1.30)	22	1.00 (1.30)	-0.15 (-0.61, 0.30)	25.54
Overall, IV	237		118		-0.09 (-0.32, 0.14)	100.00
(I^2 = 0.0%, p = 0.852)						

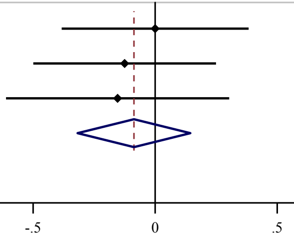

sFig15 Meta analysis of IOP after XEN surgery between those with and without prior interventions

First author (year)	N	Without Prior Intervention Mean (SD)	N	With Prior Intervention Mean (SD)	IOP After surgery (95% CI)	% Weight
Hengerer, F. H.(2019)	45	14.20 (3.40)	103	14.30 (4.20)	-0.03 (-0.38, 0.33)	49.56
Sheybani, A.(2016)	27	15.50 (3.65)	22	14.10 (3.90)	0.37 (-0.20, 0.94)	18.85
Widder, R. A.(2020)	60	14.20 (3.70)	30	13.70 (5.00)	0.12 (-0.32, 0.56)	31.60
Overall, IV	132		155		0.10 (-0.15, 0.34)	100.00
(I^2 = 0.0%, p = 0.502)						

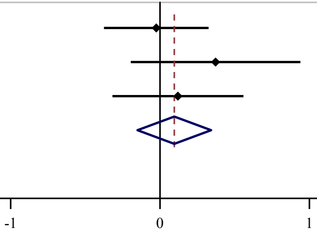

sFig16 Meta analysis of NOAM after XEN surgery between those with and without prior interventions

First author (year)	N	Without Prior Intervention Mean (SD)	N	With Prior Intervention Mean (SD)	Medication After surgery (95% CI)	% Weight
Hengerer, F. H.(2019)	45	0.30 (0.70)	103	0.30 (0.70)	0.00 (-0.35, 0.35)	36.23
Sheybani, A.(2016)	27	1.50 (0.50)	22	1.00 (0.50)	1.00 (0.40, 1.60)	29.76
Widder, R. A.(2020)	60	0.30 (0.60)	30	0.50 (1.10)	-0.25 (-0.69, 0.19)	34.01
Overall, DL	132		155		0.21 (-0.41, 0.84)	100.00
(I^2 = 82.5%, p = 0.003)						

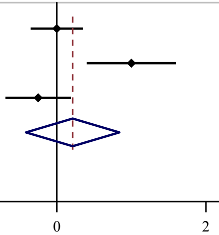

NOTE: Weights are from random-effects model

sFig17 Meta analysis of bleb needling rate between those with and without prior interventions

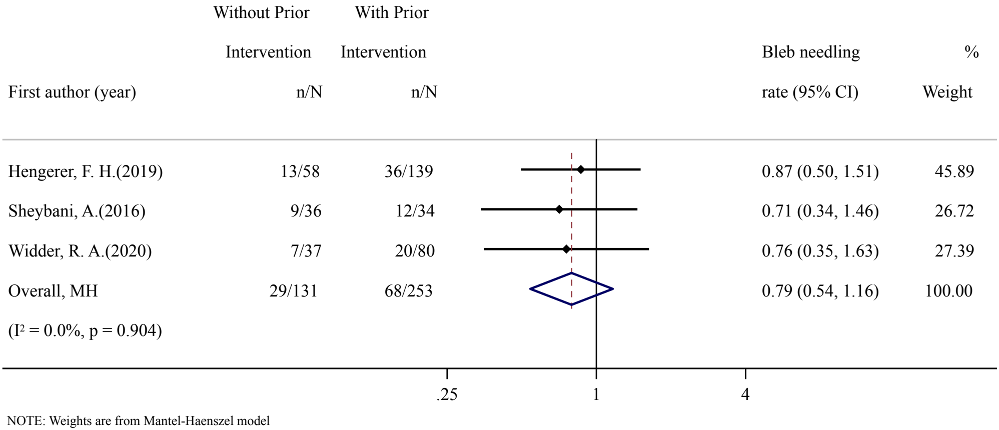

sFig18 Meta analysis of IOP after XEN surgery between phakic and pseudophakic eyes

First author (year)	Phakic eye N	Phakic eye Mean (SD)	Pseudophakic N	Pseudophakic Mean (SD)	IOP After surgery (95% CI)	% Weight
Fea, A. M.(2020)	43	14.70 (2.50)	72	15.70 (2.50)	-0.40 (-0.78, -0.02)	58.33
Olgun, A.(2020)	51	14.20 (2.20)	18	13.70 (2.80)	0.21 (-0.33, 0.75)	29.25
Wałek, E.(2020)	9	13.00 (1.00)	15	13.00 (2.10)	0.00 (-0.83, 0.83)	12.42
Overall, IV	103		105		-0.17 (-0.46, 0.12)	100.00
(I^2 = 42.6%, p = 0.175)						

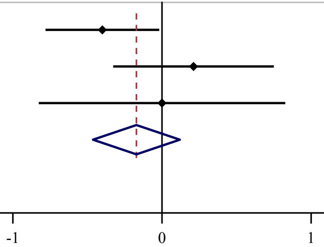

sFig19 Meta analysis of NOAM after XEN surgery between phakic and pseudophakic eyes

First author (year)	Phakic eye N	Phakic eye Mean (SD)	Pseudophakic N	Pseudophakic Mean (SD)	Medication After surgery (95% CI)	% Weight
Fea, A. M.(2020)	43	0.47 (0.90)	72	0.58 (0.90)	-0.12 (-0.50, 0.26)	67.08
Olgun, A.(2020)	51	2.00 (2.20)	18	1.40 (1.40)	0.30 (-0.24, 0.84)	32.92
Wałek, E.(2020)	9	0.00 (0.00)	15	0.00 (0.50)	(Insufficient data)	
Overall, IV	103		105		0.02 (-0.29, 0.33)	100.00
(I^2 = 35.4%, p = 0.214)						

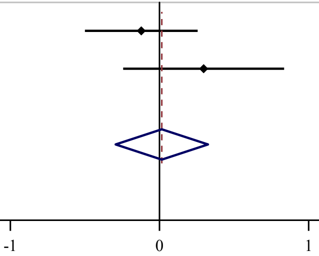

sFig20 Meta analysis of IOP by ethnicity before and after phaco-XEN surgery

Continents and First author (year)	Country	Design	N	Before XEN Mean (SD)	N	After XEN Mean (SD)	IOP (95% CI)	% Weight
Hu, J. Y.(2020)	Singapore	Retrospective Study	44	17.00 (3.35)	25	15.00 (2.25)	0.67 (0.16, 1.17)	36.96
Olgun, A.(2020)	Turkey	Retrospective Study	45	24.80 (3.50)	45	13.40 (1.40)	4.28 (3.52, 5.03)	36.53
Ozal, S.A.(2017)	Turkey	Retrospective Study	6	35.20 (3.20)	6	15.50 (2.30)	7.07 (3.77, 10.37)	26.50
Asia								
Subgroup, DL			95		76		3.68 (0.53, 6.84)	100.00
(I² = 97.2%, p = 0.000)								
Lenzhofer, M.(2019)	Austria	Prospective Study	68	23.40 (6.30)	68	12.70 (6.88)	1.62 (1.23, 2.01)	3.92
Lenzhofer, M.(2019)	Austria	Prospective Study	44	23.80 (6.10)	40	15.30 (4.60)	1.56 (1.07, 2.05)	3.72
Reitsamer, H.(2019)	Austria	Prospective Study	75	20.60 (3.60)	75	14.30 (2.70)	1.98 (1.59, 2.37)	3.92
Buffault, J.(2020)	France	Retrospective Study	30	19.90 (5.80)	30	17.10 (5.00)	0.52 (0.00, 1.03)	3.67
Galal, A.(2017)	Germany	Prospective Study	13	16.00 (4.00)	13	12.00 (3.00)	1.13 (0.30, 1.96)	2.97
Hengerer, F. H.(2017)	Germany	Retrospective Study	39	35.72 (12.00)	39	13.86 (2.46)	2.52 (1.93, 3.12)	3.49
Widder, R. A. (2018)	Germany	Retrospective Study	49	23.10 (7.30)	49	18.00 (7.30)	0.70 (0.29, 1.11)	3.88
De Gregorio, A.(2018)	Italy	Prospective Study	33	22.50 (3.70)	33	13.10 (2.40)	3.01 (2.30, 3.73)	3.24
Fea, A. M.(2020)	Italy	Prospective Study	56	21.40 (7.00)	48	15.81 (3.90)	0.97 (0.56, 1.37)	3.89
Oddone, F.(2020)	Italy	Prospective Study	40	23.00 (5.90)	40	13.70 (3.90)	1.86 (1.33, 2.39)	3.65
Scheres, M. J.（2020）	Netherlands	Retrospective Study	15	18.40 (3.30)	10	12.50 (1.70)	2.12 (1.11, 3.13)	2.59
Wałek, E.(2020)	Poland	Prospective Study	15	19.00 (2.00)	15	13.00 (2.00)	3.00 (1.94, 4.06)	2.48
Barão, R.C.(2020)	Portugal	Retrospective Study	28	21.60 (7.05)	28	16.90 (8.40)	0.61 (0.07, 1.14)	3.63
Marques, R. E.(2019)	Portugal	Retrospective Study	31	22.19 (7.79)	31	14.92 (3.32)	1.21 (0.67, 1.76)	3.61
Lavin-Dapena, C.(2020)	Spain	Prospective Study	9	16.60 (3.40)	9	15.40 (3.00)	0.37 (-0.56, 1.31)	2.75
Olate-Pérez, Á. (2017)	Spain	Prospective Study	30	21.20 (3.40)	30	14.80 (1.89)	2.33 (1.67, 2.99)	3.36
Pérez-Torregrosa, V. T.(2016)	Spain	Prospective Study	30	21.20 (3.07)	30	15.03 (0.17)	2.84 (2.11, 3.56)	3.21
Ibáñez-Muñoz A.(2019)	Spain	Retrospective Study	8	21.63 (4.39)	8	16.50 (2.50)	1.44 (0.32, 2.55)	2.38
Ibáñez-Muñoz, A.(2020)	Spain	Retrospective Study	33	22.00 (2.50)	33	15.50 (1.30)	3.26 (2.52, 4.01)	3.17
Laborda-Guirao T.(2020)	Spain	Retrospective Study	40	20.10 (5.10)	40	14.90 (0.90)	1.42 (0.93, 1.91)	3.72
Marcos Parra, M.T.(2019)	Spain	Retrospective Study	48	18.00 (4.50)	48	14.90 (1.50)	0.92 (0.50, 1.35)	3.86
Arnljots TS.(2018)	Sweden	Retrospective Study	7	23.80 (8.80)	7	17.50 (3.50)	0.94 (-0.17, 2.05)	2.39
Gillmann, K.(2019)	Switzerland	Prospective Study	41	19.00 (2.30)	41	14.70 (1.00)	2.42 (1.85, 3.00)	3.55
Gillmann, K.(2020)	Switzerland	Prospective Study	66	20.00 (6.90)	66	12.90 (3.40)	1.31 (0.93, 1.68)	3.94
Mansouri, K.(2019)	Switzerland	Prospective Study	109	20.00 (7.10)	109	14.20 (3.70)	1.02 (0.74, 1.31)	4.09
Midha, N.(2019)	Switzerland	Prospective Study	109	19.45 (5.75)	109	11.81 (5.36)	1.37 (1.08, 1.67)	4.07
Midha, N.(2020)	Switzerland	Prospective Study	38	22.00 (8.60)	38	13.90 (4.10)	1.20 (0.71, 1.69)	3.73
Gabbay, I. E.(2020)	UK	Prospective Study	57	21.00 (6.50)	57	13.90 (3.30)	1.38 (0.97, 1.79)	3.88
Karimi, A.(2018)	UK	Retrospective Study	72	18.30 (1.20)	18	13.80 (1.80)	3.37 (2.65, 4.08)	3.23
Europe								
Subgroup, DL			1233		1162		1.64 (1.38, 1.89)	100.00
(I² = 85.8%, p = 0.000)								
Sheybani, A.(2015)	US	Prospective Study	37	22.40 (4.20)	34	15.40 (2.30)	2.04 (1.47, 2.62)	34.17
Rather, P.A. (2020)	US	Retrospective Study	19	15.90 (4.70)	19	14.00 (3.50)	0.46 (-0.19, 1.10)	33.07
Kalina AG.(2019)	USA	Prospective Study	27	20.98 (6.47)	18	13.61 (2.90)	1.38 (0.71, 2.04)	32.75
North America								
Subgroup, DL			83		71		1.30 (0.38, 2.22)	100.00
(I² = 84.5%, p = 0.002)								
Heterogeneity between groups: p = 0.347								
Overall, DL			1411		1309		1.68 (1.42, 1.95)	
(I² = 88.1%, p = 0.000)								

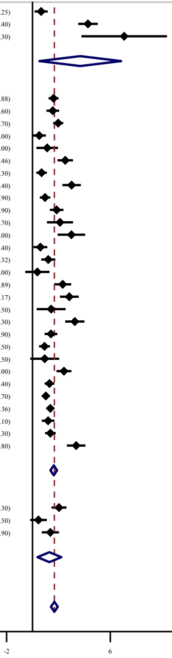

NOTE: Weights and between-subgroup heterogeneity test are from random-effects model

sFig21 Meta analysis of IOP by follow-up duration before and after phaco-XEN surgery

Follow-up and First author (year)	Country	Design	N	Before XEN Mean (SD)	N	After XEN Mean (SD)	IOP (95% CI)	% Weight
Lenzhofer, M.(2019)	Austria	Prospective Study	44	23.80 (6.10)	40	15.30 (4.60)	1.56 (1.07, 2.05)	7.02
Galal, A.(2017)	Germany	Prospective Study	13	16.00 (4.00)	13	12.00 (3.00)	1.13 (0.30, 1.96)	5.92
Hengerer, F. H.(2017)	Germany	Retrospective Study	39	35.72 (12.00)	39	13.86 (2.46)	2.52 (1.93, 3.12)	6.69
De Gregorio, A.(2018)	Italy	Prospective Study	33	22.50 (3.70)	33	13.10 (2.40)	3.01 (2.30, 3.73)	6.33
Fea, A. M.(2020)	Italy	Prospective Study	56	21.40 (7.00)	48	15.81 (3.90)	0.97 (0.56, 1.37)	7.24
Olate-Pérez, Á. (2017)	Spain	Prospective Study	30	21.20 (3.40)	30	14.80 (1.89)	2.33 (1.67, 2.99)	6.50
Pérez-Torregrosa, V. T.(2016)	Spain	Prospective Study	30	21.20 (3.07)	30	15.03 (0.17)	2.84 (2.11, 3.56)	6.29
Ibáñez-Muñoz A.(2019)	Spain	Retrospective Study	8	21.63 (4.39)	8	16.50 (2.50)	1.44 (0.32, 2.55)	4.97
Ibáñez-Muñoz, A.(2020)	Spain	Retrospective Study	33	22.00 (2.50)	33	15.50 (1.30)	3.26 (2.52, 4.01)	6.22
Laborda-Guirao T.(2020)	Spain	Retrospective Study	40	20.10 (5.10)	40	14.90 (0.90)	1.42 (0.93, 1.91)	7.01
Marcos Parra, M.T.(2019)	Spain	Retrospective Study	48	18.00 (4.50)	48	14.90 (1.50)	0.92 (0.50, 1.35)	7.20
Mansouri, K.(2019)	Switzerland	Prospective Study	109	20.00 (7.10)	109	14.20 (3.70)	1.02 (0.74, 1.31)	7.51
Ozal, S.A.(2017)	Turkey	Retrospective Study	6	35.20 (3.20)	6	15.50 (2.30)	7.07 (3.77, 10.37)	1.31
Sheybani, A.(2015)	US	Prospective Study	37	22.40 (4.20)	34	15.40 (2.30)	2.04 (1.47, 2.62)	6.76
Rather, P.A. (2020)	US	Retrospective Study	19	15.90 (4.70)	19	14.00 (3.50)	0.46 (-0.19, 1.10)	6.55
Kalina AG.(2019)	USA	Prospective Study	27	20.98 (6.47)	18	13.61 (2.90)	1.38 (0.71, 2.04)	6.48
12m								
Subgroup, DL			572		548		1.80 (1.39, 2.22)	100.00
(I^2 = 87.5%, p = 0.000)								
Widder, R. A. (2018)	Germany	Retrospective Study	49	23.10 (7.30)	49	18.00 (7.30)	0.70 (0.29, 1.11)	26.28
Barão, R.C.(2020)	Portugal	Retrospective Study	28	21.60 (7.05)	28	16.90 (8.40)	0.61 (0.07, 1.14)	25.71
Lavin-Dapena, C.(2020)	Spain	Prospective Study	9	16.60 (3.40)	9	15.40 (3.00)	0.37 (-0.56, 1.31)	23.30
Karimi, A.(2018)	UK	Retrospective Study	72	18.30 (1.20)	18	13.80 (1.80)	3.37 (2.65, 4.08)	24.72
18m								
Subgroup, DL			158		104		1.26 (0.06, 2.46)	100.00
(I^2 = 93.7%, p = 0.000)								
Lenzhofer, M.(2019)	Austria	Prospective Study	68	23.40 (6.30)	68	12.70 (6.88)	1.62 (1.23, 2.01)	11.48
Reitsamer, H.(2019)	Austria	Prospective Study	75	20.60 (3.60)	75	14.30 (2.70)	1.98 (1.59, 2.37)	11.47
Scheres, M. J.（2020）	Netherlands	Retrospective Study	15	18.40 (3.30)	10	12.50 (1.70)	2.12 (1.11, 3.13)	7.93
Wałek, E.(2020)	Poland	Prospective Study	15	19.00 (2.00)	15	13.00 (2.00)	3.00 (1.94, 4.06)	7.62
Arnljots TS.(2018)	Sweden	Retrospective Study	7	23.80 (8.80)	7	17.50 (3.50)	0.94 (-0.17, 2.05)	7.34
Gillmann, K.(2019)	Switzerland	Prospective Study	41	19.00 (2.30)	41	14.70 (1.00)	2.42 (1.85, 3.00)	10.51
Midha, N.(2019)	Switzerland	Prospective Study	109	19.45 (5.75)	109	11.81 (5.36)	1.37 (1.08, 1.67)	11.87
Midha, N.(2020)	Switzerland	Prospective Study	38	22.00 (8.60)	38	13.90 (4.10)	1.20 (0.71, 1.69)	10.97
Olgun, A.(2020)	Turkey	Retrospective Study	45	24.80 (3.50)	45	13.40 (1.40)	4.28 (3.52, 5.03)	9.43
Gabbay, I. E.(2020)	UK	Prospective Study	57	21.00 (6.50)	57	13.90 (3.30)	1.38 (0.97, 1.79)	11.38
24m								
Subgroup, DL			470		465		1.99 (1.52, 2.46)	100.00
(I^2 = 87.5%, p = 0.000)								
Gillmann, K.(2020)	Switzerland	Prospective Study	66	20.00 (6.90)	66	12.90 (3.40)	1.31 (0.93, 1.68)	100.00
36m								
Subgroup, DL			66		66		1.31 (0.93, 1.68)	100.00
(I^2 = 0.0%, p = .)								
Buffault, J.(2020)	France	Retrospective Study	30	19.90 (5.80)	30	17.10 (5.00)	0.52 (0.00, 1.03)	25.13
Oddone, F.(2020)	Italy	Prospective Study	40	23.00 (5.90)	40	13.70 (3.90)	1.86 (1.33, 2.39)	24.92
Marques, R. E.(2019)	Portugal	Retrospective Study	31	22.19 (7.79)	31	14.92 (3.32)	1.21 (0.67, 1.76)	24.61
Hu, J. Y.(2020)	Singapore	Retrospective Study	44	17.00 (3.35)	25	15.00 (2.25)	0.67 (0.16, 1.17)	25.33
6m								
Subgroup, DL			145		126		1.06 (0.46, 1.66)	100.00
(I^2 = 81.0%, p = 0.001)								
Heterogeneity between groups: p = 0.056								
Overall, DL			1411		1309		1.68 (1.42, 1.95)	
(I^2 = 88.1%, p = 0.000)								

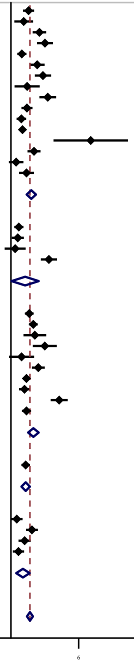

NOTE: Weights and between-subgroup heterogeneity test are from random-effects model

sFig22 Meta analysis of NOAM before and after phaco-XEN surgery

First author (year)	Country	Design	N (Before XEN)	Mean (SD) (Before XEN)	N (After XEN)	Mean (SD) (After XEN)	Medication (95% CI)	% Weight
De Gregorio, A.(2018)	Italy	Prospective Study	33	2.50 (0.90)	33	0.40 (0.80)	2.47 (1.82, 3.11)	6.80
Fea, A. M.(2020)	Italy	Prospective Study	56	2.51 (1.10)	48	0.50 (1.00)	1.91 (1.44, 2.37)	7.60
Gabbay, I. E.(2020)	UK	Prospective Study	57	2.70 (1.10)	57	0.50 (0.10)	2.82 (2.30, 3.34)	7.36
Hong, K.(2020)	US	Prospective Study	28	3.80 (0.90)	28	1.30 (1.00)	2.63 (1.91, 3.35)	6.44
Lavin-Dapena, C.(2020)	Spain	Prospective Study	9	2.00 (0.90)	9	0.10 (0.30)	2.83 (1.48, 4.18)	3.90
Reitsamer, H.(2019)	Austria	Prospective Study	88	2.50 (0.90)	75	1.00 (1.00)	1.58 (1.23, 1.94)	8.04
Sheybani, A.(2015)	US	Prospective Study	37	2.50 (1.40)	34	0.90 (0.50)	1.50 (0.97, 2.03)	7.33
Wałek, E.(2020)	Poland	Prospective Study	15	3.00 (1.00)	15	0.00 (0.50)	3.79 (2.57, 5.02)	4.31
Barão, R.C.(2020)	Portugal	Retrospective Study	28	2.46 (1.20)	28	0.71 (1.08)	1.53 (0.93, 2.13)	7.01
Hengerer, F. H.(2017)	Germany	Retrospective Study	39	3.26 (1.00)	39	0.36 (0.73)	3.31 (2.62, 4.00)	6.59
Hu, J. Y.(2020)	Singapore	Retrospective Study	44	3.00 (1.00)	25	0.56 (1.05)	2.40 (1.76, 3.03)	6.83
Laborda-Guirao T.(2020)	Spain	Retrospective Study	40	2.70 (0.61)	40	1.70 (0.40)	1.94 (1.41, 2.47)	7.31
Marcos Parra, M.T.(2019)	Spain	Retrospective Study	48	2.10 (0.90)	48	0.10 (0.30)	2.98 (2.40, 3.57)	7.08
Olgun, A.(2020)	Turkey	Retrospective Study	45	3.40 (0.50)	45	1.80 (1.70)	1.28 (0.82, 1.73)	7.65
Scheres, M. J.（2020）	Netherlands	Retrospective Study	15	2.10 (1.50)	10	0.50 (1.10)	1.18 (0.31, 2.05)	5.76
Overall, DL			582		534		2.21 (1.85, 2.57)	100.00
(I^2 = 81.2%, p = 0.000)								

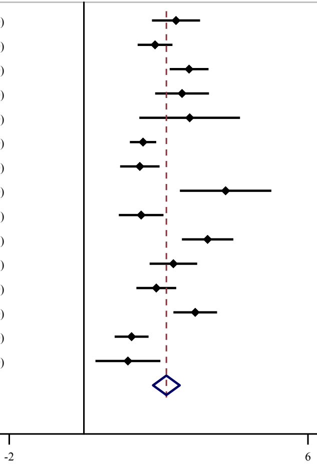

NOTE: Weights are from random-effects model

sFig23 Meta analysis of IOP after surgery between XEN-only and phaco-XEN surgery

First author (year)	Country	Design	XEN only N	XEN only Mean (SD)	phaco-XEN N	phaco-XEN Mean (SD)	IOP change (95% CI)	% Weight
Lenzhofer, M.(2019)	Austria	Prospective Study	69	13.00 (5.15)	68	12.70 (6.88)	0.05 (-0.29, 0.38)	6.54
Lenzhofer, M.(2019)	Austria	Prospective Study	32	15.00 (6.20)	40	15.30 (4.60)	-0.06 (-0.52, 0.41)	3.40
Reitsamer, H.(2019)	Austria	Prospective Study	86	14.40 (3.40)	75	14.30 (2.70)	0.03 (-0.28, 0.34)	7.65
Fea, A. M.(2020)	Italy	Prospective Study	89	15.36 (3.90)	48	15.81 (3.90)	-0.12 (-0.47, 0.24)	5.95
Oddone, F.(2020)	Italy	Prospective Study	68	12.40 (2.80)	40	13.70 (3.90)	-0.40 (-0.79, -0.01)	4.72
Wałek, E.(2020)	Poland	Prospective Study	9	13.00 (1.00)	15	13.00 (2.00)	0.00 (-0.83, 0.83)	1.07
Gillmann, K.(2019)	Switzerland	Prospective Study	16	14.90 (2.50)	41	14.70 (1.00)	0.13 (-0.45, 0.71)	2.19
Gillmann, K.(2020)	Switzerland	Prospective Study	26	12.90 (2.90)	66	12.90 (3.40)	0.00 (-0.45, 0.45)	3.56
Mansouri, K.(2019)	Switzerland	Prospective Study	40	13.80 (4.10)	109	14.20 (3.70)	-0.10 (-0.47, 0.26)	5.58
Midha, N.(2019)	Switzerland	Prospective Study	40	10.33 (4.41)	109	11.81 (5.36)	-0.29 (-0.65, 0.08)	5.54
Midha, N.(2020)	Switzerland	Prospective Study	13	13.40 (4.60)	38	13.90 (4.10)	-0.12 (-0.75, 0.51)	1.85
Gabbay, I. E.(2020)	UK	Prospective Study	94	15.00 (3.30)	57	13.90 (3.30)	0.33 (0.00, 0.66)	6.69
Kalina AG.(2019)	US	Prospective Study	14	13.04 (4.50)	18	13.61 (2.90)	-0.15 (-0.85, 0.54)	1.50
Buffault, J.(2020)	France	Retrospective Study	77	14.80 (5.90)	30	17.10 (5.00)	-0.41 (-0.83, 0.02)	4.06
Hengerer, F. H.(2017)	Germany	Retrospective Study	200	14.33 (4.19)	39	13.86 (2.46)	0.12 (-0.22, 0.46)	6.23
Widder, R. A. (2018)	Germany	Retrospective Study	45	18.80 (8.40)	49	18.00 (7.30)	0.10 (-0.30, 0.51)	4.48
Scheres, M. J.（2020）	Netherlands	Retrospective Study	16	14.60 (4.60)	10	12.50 (1.70)	0.56 (-0.25, 1.36)	1.13
Barão, R.C.(2020)	Portugal	Retrospective Study	14	20.90 (11.60)	28	16.90 (8.40)	0.42 (-0.23, 1.07)	1.75
Marques, R. E.(2019)	Portugal	Retrospective Study	29	15.00 (7.27)	31	14.92 (3.32)	0.01 (-0.49, 0.52)	2.86
Hu, J. Y.(2020)	Singapore	Retrospective Study	11	18.00 (4.00)	25	15.00 (2.25)	1.04 (0.29, 1.79)	1.30
Ibáñez-Muñoz A.(2019)	Spain	Retrospective Study	13	14.38 (4.52)	8	16.50 (2.50)	-0.54 (-1.44, 0.35)	0.91
Ibáñez-Muñoz, A.(2020)	Spain	Retrospective Study	40	15.00 (1.50)	33	15.50 (1.30)	-0.35 (-0.82, 0.11)	3.40
Laborda-Guirao T.(2020)	Spain	Retrospective Study	40	14.40 (0.90)	40	14.90 (0.90)	-0.56 (-1.00, -0.11)	3.68
Marcos Parra, M.T.(2019)	Spain	Retrospective Study	17	15.50 (3.70)	48	14.90 (1.50)	0.26 (-0.29, 0.82)	2.38
Arnljots TS.(2018)	Sweden	Retrospective Study	12	17.00 (5.50)	7	17.50 (3.50)	-0.10 (-1.04, 0.83)	0.84
Olgun, A.(2020)	Turkey	Retrospective Study	51	14.20 (2.20)	45	13.40 (1.40)	0.43 (0.02, 0.83)	4.46
Ozal, S.A.(2017)	Turkey	Retrospective Study	9	17.60 (4.20)	6	15.50 (2.30)	0.58 (-0.47, 1.64)	0.66
Karimi, A.(2018)-2	UK	Retrospective Study	71	14.30 (2.10)	18	13.80 (1.80)	0.24 (-0.27, 0.76)	2.73
Rather, P.A. (2020)	US	Retrospective Study	73	13.60 (3.50)	19	14.00 (3.50)	-0.11 (-0.62, 0.39)	2.88
Overall, IV ($I^2 = 35.9\%$, $p = 0.030$)			1314		1160		-0.01 (-0.09, 0.08)	100.00

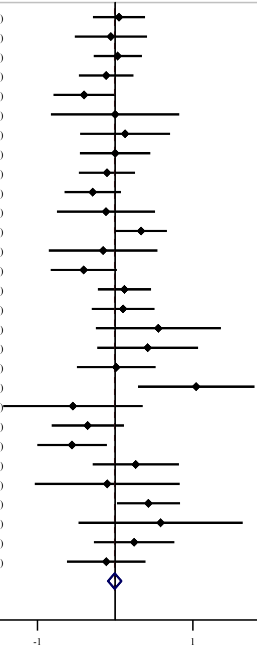

sFig24 Meta analysis of IOP after surgery by ethnicity between XEN-only and phaco-XEN surgery

Continents and First author (year)	Country	Design	N	XEN only Mean (SD)	N	phaco-XEN Mean (SD)	IOP After surgery (95% CI)	% Weight
Hu, J. Y.(2020)	Singapore	Retrospective Study	11	18.00 (4.00)	25	15.00 (2.25)	1.04 (0.29, 1.79)	20.26
Olgun, A.(2020)	Turkey	Retrospective Study	51	14.20 (2.20)	45	13.40 (1.40)	0.43 (0.02, 0.83)	69.52
Ozal, S.A.(2017)	Turkey	Retrospective Study	9	17.60 (4.20)	6	15.50 (2.30)	0.58 (-0.47, 1.64)	10.23
Asia								
Subgroup, IV			71		76		0.57 (0.23, 0.91)	100.00
(I² = 0.0%, p = 0.369)								
Lenzhofer, M.(2019)	Austria	Prospective Study	69	13.00 (5.15)	68	12.70 (6.88)	0.05 (-0.29, 0.38)	7.33
Lenzhofer, M.(2019)	Austria	Prospective Study	32	15.00 (6.20)	40	15.30 (4.60)	-0.06 (-0.52, 0.41)	3.81
Reitsamer, H.(2019)	Austria	Prospective Study	86	14.40 (3.40)	75	14.30 (2.70)	0.03 (-0.28, 0.34)	8.58
Buffault, J.(2020)	France	Retrospective Study	77	14.80 (5.90)	30	17.10 (5.00)	-0.41 (-0.83, 0.02)	4.55
Hengerer, F. H.(2017)	Germany	Retrospective Study	200	14.33 (4.19)	39	13.86 (2.46)	0.12 (-0.22, 0.46)	6.98
Widder, R. A. (2018)	Germany	Retrospective Study	45	18.80 (8.40)	49	18.00 (7.30)	0.10 (-0.30, 0.51)	5.02
Fea, A. M.(2020)	Italy	Prospective Study	89	15.36 (3.90)	48	15.81 (3.90)	-0.12 (-0.47, 0.24)	6.67
Oddone, F.(2020)	Italy	Prospective Study	68	12.40 (2.80)	40	13.70 (3.90)	-0.40 (-0.79, -0.01)	5.29
Scheres, M. J.（2020）	Netherlands	Retrospective Study	16	14.60 (4.60)	10	12.50 (1.70)	0.56 (-0.25, 1.36)	1.27
Wałek, E.(2020)	Poland	Prospective Study	9	13.00 (1.00)	15	13.00 (2.00)	0.00 (-0.83, 0.83)	1.20
Barão, R.C.(2020)	Portugal	Retrospective Study	14	20.90 (11.60)	28	16.90 (8.40)	0.42 (-0.23, 1.07)	1.96
Marques, R. E.(2019)	Portugal	Retrospective Study	29	15.00 (7.27)	31	14.92 (3.32)	0.01 (-0.49, 0.52)	3.21
Ibáñez-Muñoz A.(2019)	Spain	Retrospective Study	13	14.38 (4.52)	8	16.50 (2.50)	-0.54 (-1.44, 0.35)	1.02
Ibáñez-Muñoz, A.(2020)	Spain	Retrospective Study	40	15.00 (1.50)	33	15.50 (1.30)	-0.35 (-0.82, 0.11)	3.81
Laborda-Guirao T.(2020)	Spain	Retrospective Study	40	14.40 (0.90)	40	14.90 (0.90)	-0.56 (-1.00, -0.11)	4.12
Marcos Parra, M.T.(2019)	Spain	Retrospective Study	17	15.50 (3.70)	48	14.90 (1.50)	0.26 (-0.29, 0.82)	2.67
Arnljots TS.(2018)	Sweden	Retrospective Study	12	17.00 (5.50)	7	17.50 (3.50)	-0.10 (-1.04, 0.83)	0.95
Gillmann, K.(2019)	Switzerland	Prospective Study	16	14.90 (2.50)	41	14.70 (1.00)	0.13 (-0.45, 0.71)	2.46
Gillmann, K.(2020)	Switzerland	Prospective Study	26	12.90 (2.90)	66	12.90 (3.40)	0.00 (-0.45, 0.45)	3.99
Mansouri, K.(2019)	Switzerland	Prospective Study	40	13.80 (4.10)	109	14.20 (3.70)	-0.10 (-0.47, 0.26)	6.26
Midha, N.(2019)	Switzerland	Prospective Study	40	10.33 (4.41)	109	11.81 (5.36)	-0.29 (-0.65, 0.08)	6.22
Midha, N.(2020)	Switzerland	Prospective Study	13	13.40 (4.60)	38	13.90 (4.10)	-0.12 (-0.75, 0.51)	2.07
Gabbay, I. E.(2020)	UK	Prospective Study	94	15.00 (3.30)	57	13.90 (3.30)	0.33 (0.00, 0.66)	7.50
Karimi, A.(2018)-2	UK	Retrospective Study	71	14.30 (2.10)	18	13.80 (1.80)	0.24 (-0.27, 0.76)	3.06
Europe								
Subgroup, IV			1156		1047		-0.04 (-0.13, 0.05)	100.00
(I² = 22.6%, p = 0.158)								
Kalina AG.(2019)	US	Prospective Study	14	13.04 (4.50)	18	13.61 (2.90)	-0.15 (-0.85, 0.54)	34.27
Rather, P.A. (2020)	US	Retrospective Study	73	13.60 (3.50)	19	14.00 (3.50)	-0.11 (-0.62, 0.39)	65.73
North America								
Subgroup, IV			87		37		-0.13 (-0.54, 0.28)	100.00
(I² = 0.0%, p = 0.926)								
Heterogeneity between groups: p = 0.002								
Overall, IV			1314		1160		-0.01 (-0.09, 0.08)	
(I² = 35.9%, p = 0.030)								

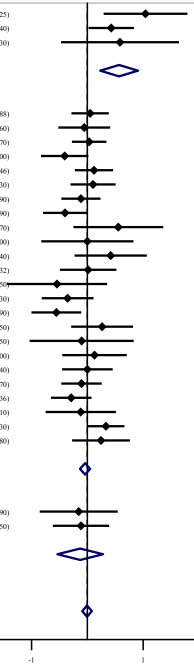

sFig25 Meta analysis of IOP before surgery by ethnicity between XEN-only and phaco-XEN surgery

Continents and First author (year)	Country	Design	XEN only N	XEN only Mean (SD)	phaco-XEN N	phaco-XEN Mean (SD)	IOP Before surgery (95% CI)	% Weight
Hu, J. Y.(2020)	Singapore	Retrospective Study	19	24.00 (4.00)	44	17.00 (3.35)	1.97 (1.33, 2.61)	33.99
Olgun, A.(2020)	Turkey	Retrospective Study	51	24.40 (4.30)	45	24.80 (3.50)	-0.10 (-0.50, 0.30)	35.48
Ozal, S.A.(2017)	Turkey	Retrospective Study	9	36.70 (4.10)	6	35.20 (3.20)	0.40 (-0.65, 1.44)	30.53
Asia								
Subgroup, DL			79		95		0.75 (-0.67, 2.18)	100.00
(I^2 = 93.1%, p = 0.000)								
Lenzhofer, M.(2019)	Austria	Prospective Study	69	22.50 (6.50)	68	23.40 (6.30)	-0.14 (-0.48, 0.19)	4.86
Lenzhofer, M.(2019)	Austria	Prospective Study	32	22.20 (4.60)	44	23.80 (6.10)	-0.29 (-0.75, 0.17)	4.09
Reitsamer, H.(2019)	Austria	Prospective Study	86	22.10 (4.00)	75	20.60 (3.60)	0.39 (0.08, 0.71)	5.00
Reitsamer, H.(2021)	Austria	Retrospective Study	116	20.40 (4.70)	96	20.40 (5.50)	0.00 (-0.27, 0.27)	5.26
Buffault, J.(2020)	France	Retrospective Study	77	20.60 (6.60)	30	19.90 (5.80)	0.11 (-0.31, 0.53)	4.31
Hengerer, F. H.(2017)	Germany	Retrospective Study	200	31.53 (8.36)	39	35.72 (12.00)	-0.46 (-0.81, -0.12)	4.80
Widder, R. A. (2018)	Germany	Retrospective Study	45	25.20 (7.30)	49	23.10 (7.30)	0.29 (-0.12, 0.69)	4.41
Fea, A. M.(2020)	Italy	Prospective Study	115	25.03 (7.60)	56	21.40 (7.00)	0.49 (0.17, 0.81)	4.94
Oddone, F.(2020)	Italy	Prospective Study	68	22.70 (4.30)	40	23.00 (5.90)	-0.06 (-0.45, 0.33)	4.51
Scheres, M. J.（2020）	Netherlands	Retrospective Study	26	19.60 (4.90)	15	18.40 (3.30)	0.27 (-0.37, 0.91)	3.10
Wałek, E.(2020)	Poland	Prospective Study	9	25.00 (3.00)	15	19.00 (2.00)	2.49 (1.38, 3.59)	1.55
Barão, R.C.(2020)	Portugal	Retrospective Study	14	25.00 (10.60)	28	21.60 (7.05)	0.41 (-0.24, 1.05)	3.05
Marques, R. E.(2019)	Portugal	Retrospective Study	29	25.52 (8.11)	31	22.19 (7.79)	0.42 (-0.09, 0.93)	3.77
Lavin-Dapena, C.(2020)	Spain	Prospective Study	2	29.00 (7.10)	9	16.60 (3.40)	3.11 (1.01, 5.21)	0.52
Ibáñez-Muñoz A.(2019)	Spain	Retrospective Study	13	20.85 (3.30)	8	21.63 (4.39)	-0.21 (-1.09, 0.67)	2.12
Ibáñez-Muñoz, A.(2020)	Spain	Retrospective Study	40	24.00 (3.00)	33	22.00 (2.50)	0.72 (0.24, 1.19)	3.99
Laborda-Guirao T.(2020)	Spain	Retrospective Study	40	21.80 (5.30)	40	20.10 (5.10)	0.33 (-0.11, 0.77)	4.20
Marcos Parra, M.T.(2019)	Spain	Retrospective Study	17	22.20 (6.80)	48	18.00 (4.50)	0.81 (0.24, 1.38)	3.44
Arnljots TS.(2018)	Sweden	Retrospective Study	12	24.90 (7.40)	7	23.80 (8.80)	0.14 (-0.79, 1.07)	1.97
Gillmann, K.(2019)	Switzerland	Prospective Study	16	20.50 (4.00)	41	19.00 (2.30)	0.52 (-0.06, 1.11)	3.36
Gillmann, K.(2020)	Switzerland	Prospective Study	26	21.00 (7.40)	66	20.00 (6.90)	0.14 (-0.31, 0.60)	4.12
Mansouri, K.(2019)	Switzerland	Prospective Study	40	21.20 (6.80)	109	20.00 (7.10)	0.17 (-0.19, 0.53)	4.69
Midha, N.(2019)	Switzerland	Prospective Study	40	20.05 (9.35)	109	19.45 (5.75)	0.09 (-0.28, 0.45)	4.69
Midha, N.(2020)	Switzerland	Prospective Study	13	23.00 (7.40)	38	22.00 (8.60)	0.12 (-0.51, 0.75)	3.14
Gabbay, I. E.(2020)	UK	Prospective Study	94	22.90 (6.50)	57	21.00 (6.50)	0.29 (-0.04, 0.62)	4.89
Karimi, A.(2018)-2	UK	Retrospective Study	186	19.60 (2.00)	72	18.30 (1.20)	0.72 (0.44, 1.00)	5.21
Europe								
Subgroup, DL			1425		1223		0.27 (0.11, 0.43)	100.00
(I^2 = 69.1%, p = 0.000)								
Kalina AG.(2019)	US	Prospective Study	20	24.18 (8.18)	27	20.98 (6.47)	0.44 (-0.14, 1.03)	42.71
Rather, P.A. (2020)	US	Retrospective Study	73	16.80 (5.40)	19	15.90 (4.70)	0.17 (-0.33, 0.68)	57.29
North America								
Subgroup, DL			93		46		0.29 (-0.10, 0.67)	100.00
(I^2 = 0.0%, p = 0.492)								
Heterogeneity between groups: p = 0.800								
Overall, DL			1597		1364		0.31 (0.15, 0.47)	
(I^2 = 73.3%, p = 0.000)								

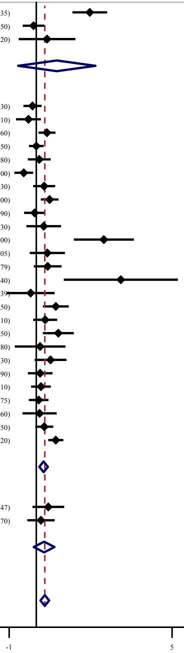

NOTE: Weights and between-subgroup heterogeneity test are from random-effects model

sFig26 sensitive analysis of IOP before and after XEN surgery

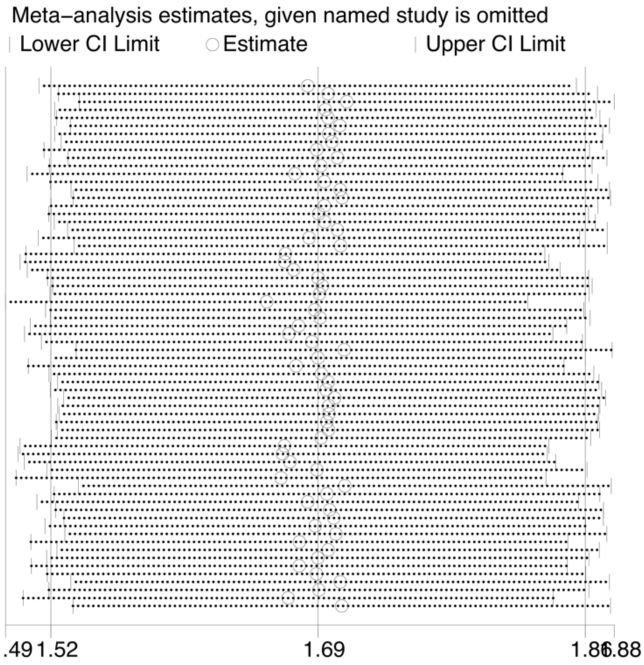

sFig27 sensitive analysis of NOAM before and after XEN surgery

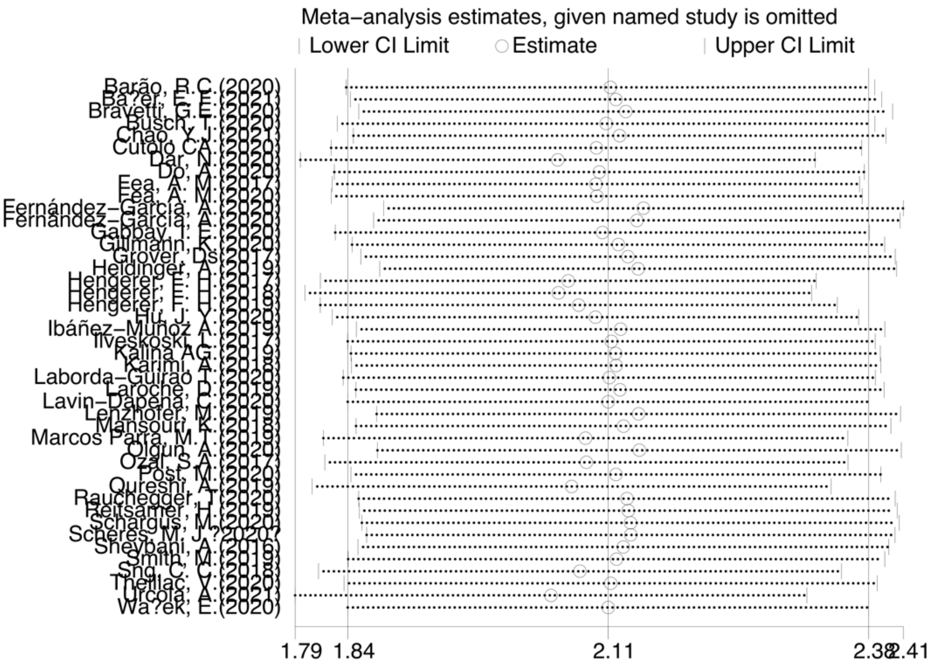

sFig28 sensitive analysis of IOP before and after phaco-XEN surgery

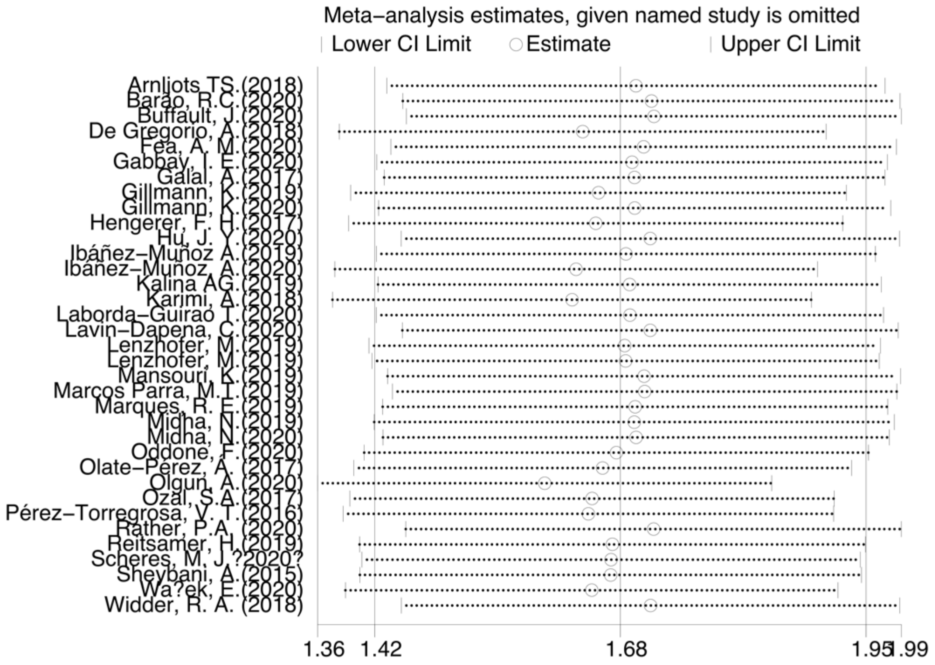

sFig29 sensitive analysis of NOAM before and after phaco-XEN surgery

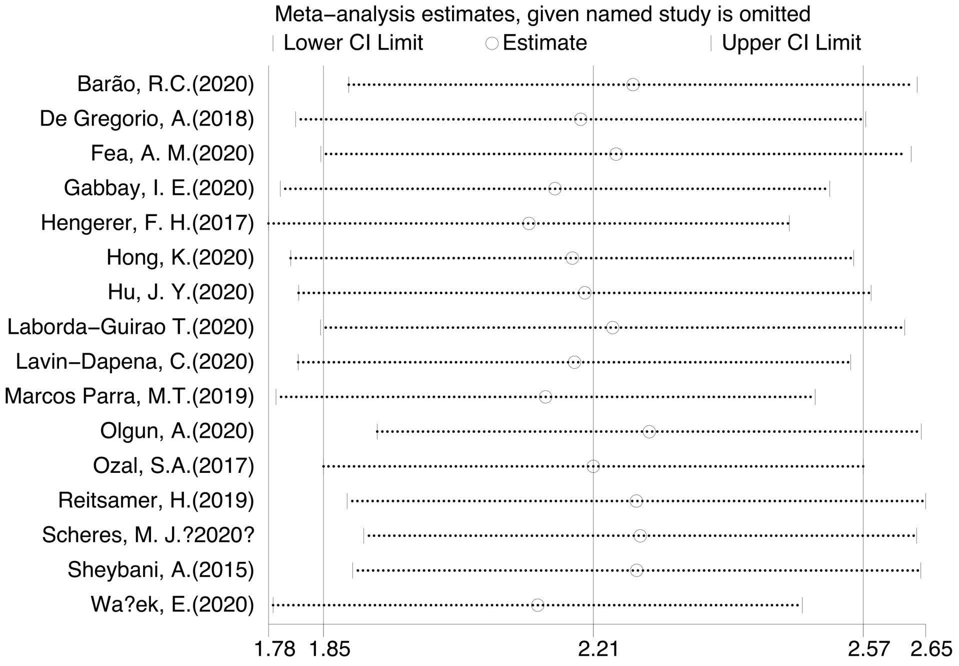

sFig30 sensitive analysis of IOP before XEN surgery and phaco-XEN surgery

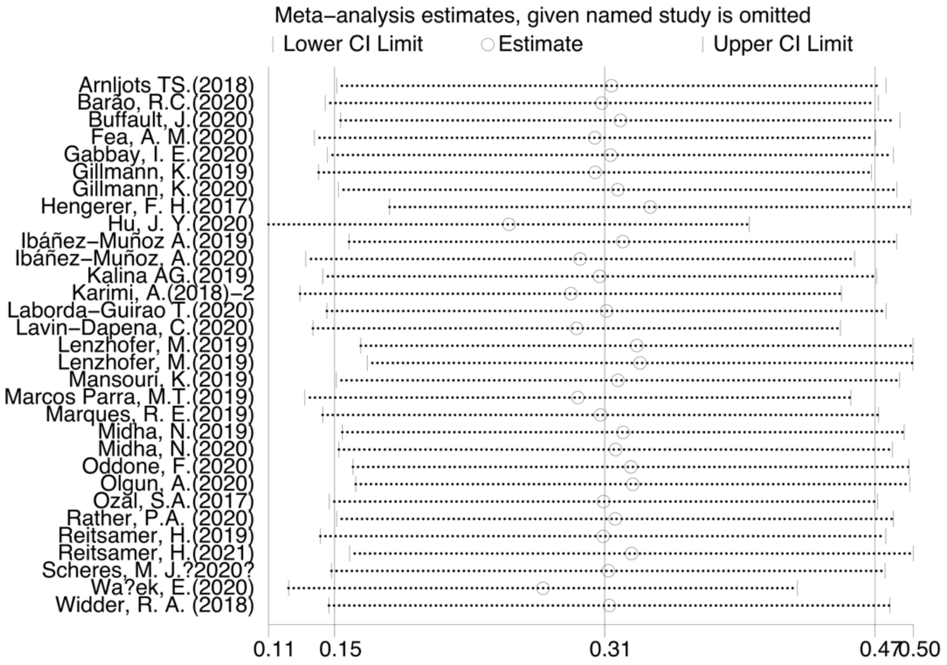

sFig31 sensitive analysis of IOP after XEN surgery and phaco-XEN surgery

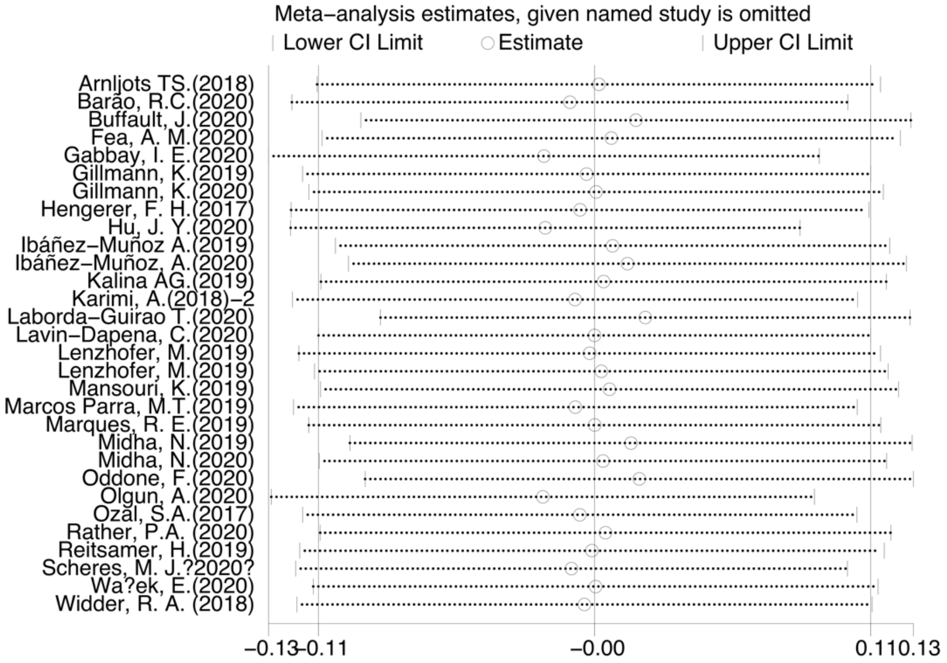

sFig32 sensitive analysis of NOAM before XEN surgery and phaco-XEN surgery

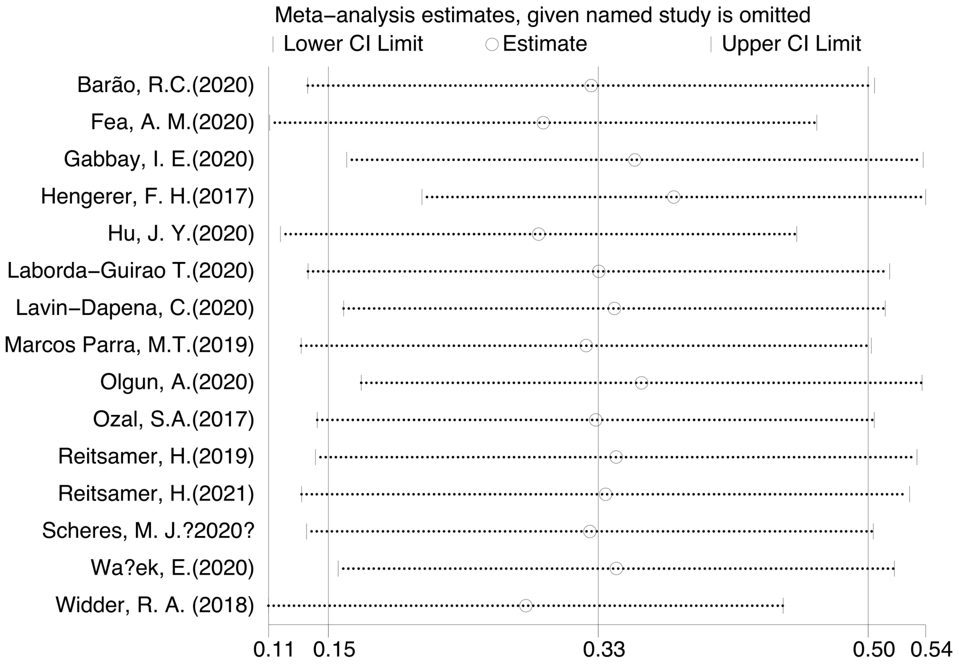

sFig33 sensitive analysis of NOAM after XEN surgery and phaco-XEN surgery

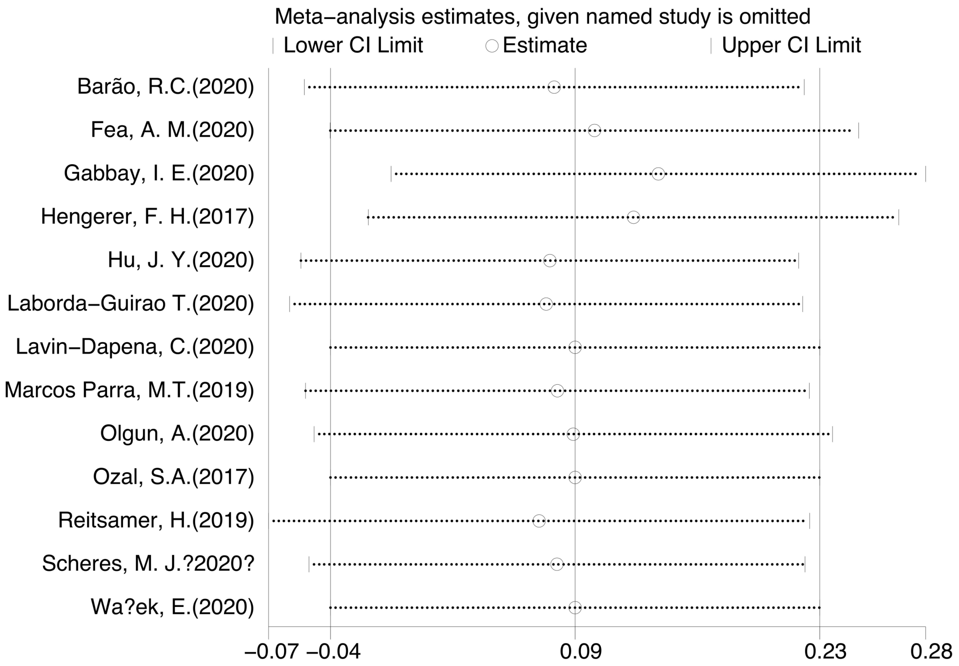

sFig34 filled funnel plot of IOP before and after phaco-XEN surgery

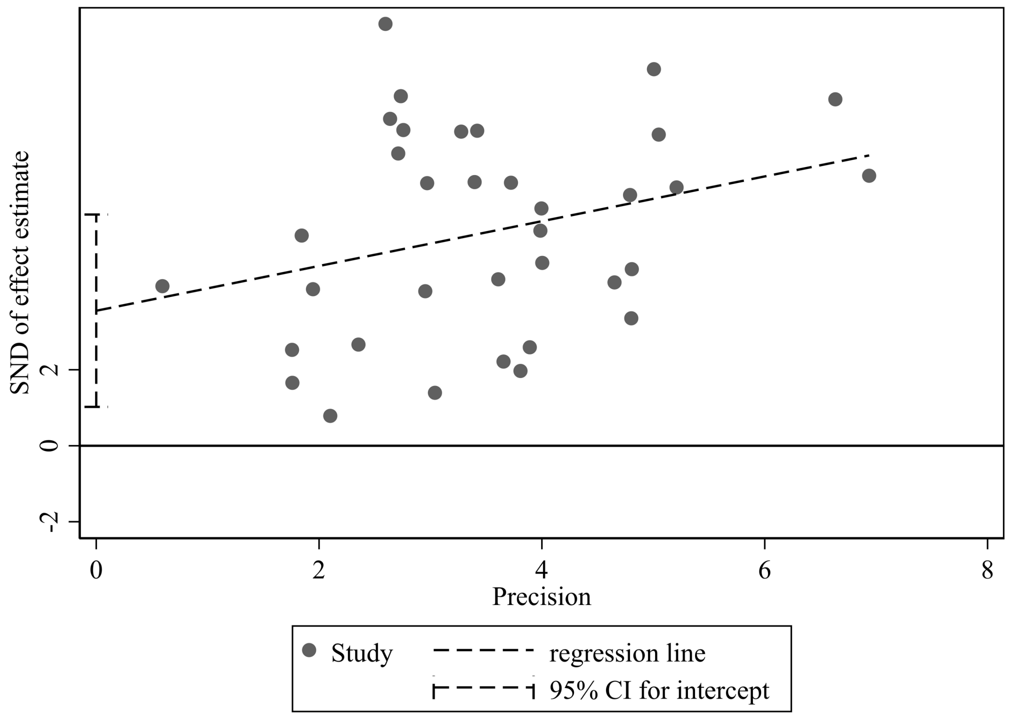

sFig35 filled funnel plot of NOAM before and after phaco-XEN surgery

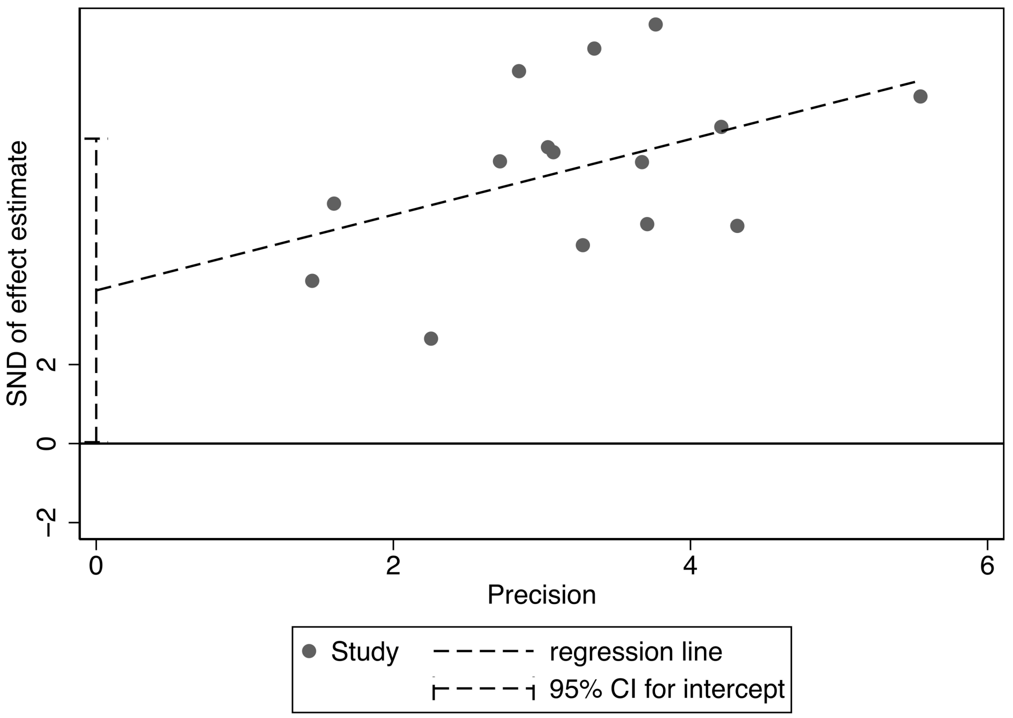

sFig36 filled funnel plot of NOAM after XEN and phaco-XEN surgery

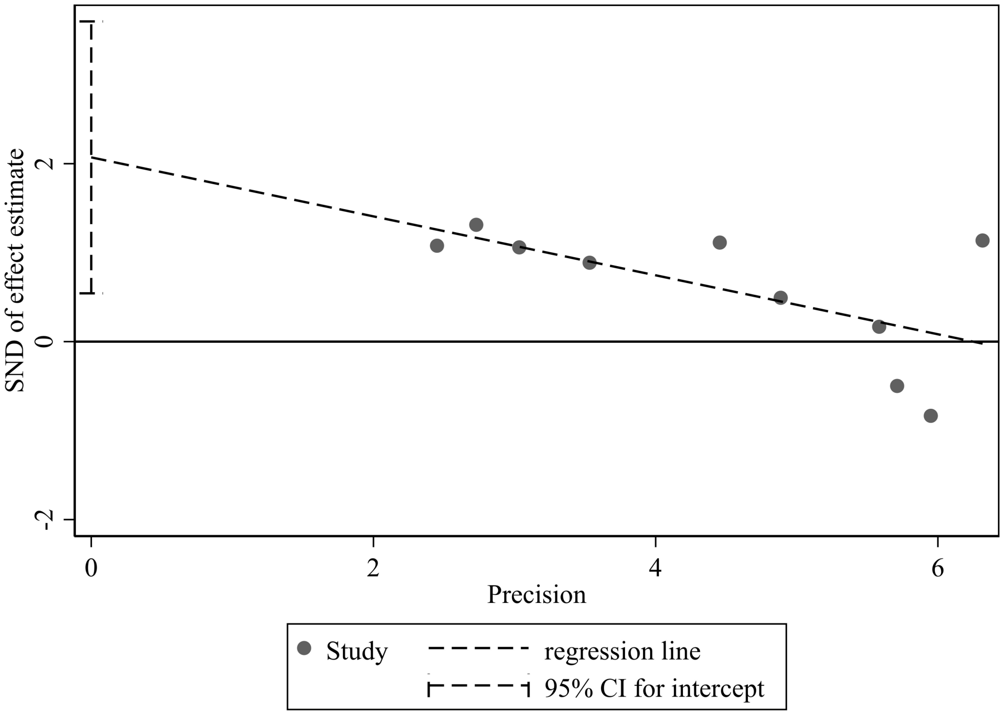

Supplement: Supplementary file 4 — Additional file 4:SFig1. Meta analysis of IOP by study design before and after XEN surgery. SFig2. Meta analysis of NOAM by study design before and after XEN surgery. sFig3. Meta analysis of IOP before and after XEN surgery. sFig4. Meta analysis of NOAM before and after XEN surgery. sFig5. Meta analysis of IOP by follow-up duration before and after XEN surgery. sFig6. Meta analysis of NOAM by follow-up duration before and after XEN surgery. sFig7. Meta analysis of IOP by ethnicity before and after XEN surgery. sFig8. Meta analysis of IOP by developed and developing country and after XEN surgery. sFig9. Meta analysis of NOAM by developed and developing country before and after XEN surgery. sFig10. Meta analysis of NOAM by ethnicity before after XEN surgery. sFig11. Meta analysis of IOP before XEN surgery betweem POAG and PEXG. sFig12. Meta analysis of IOP after XEN surgery betweem POAG and PEXG. sFig13. Meta analysis of NOAM before XEN surgery betweem POAG and PEXG. sFig14. Meta analysis of NOAM after XEN surgery betweem POAG and PEXG. sFig15. Meta analysis of IOP after XEN surgery between those with and without prior interventions. sFig16. Meta analysis of NOAM after XEN surgery between those with and without prior interventions. sFig17. Meta analysis of bleb needling rate between those with and without prior interventions. sFig18. Meta analysis of IOP after XEN surgery between phakic and pseudophakic eyes. sFig19. Meta analysis of NOAM after XEN surgery between phakic and pseudophakic eyes. sFig20. Meta analysis of IOP by ethnicity before and after phaco-XEN surgery. sFig21. Meta analysis of IOP by follow-up duration before and after phaco-XEN surgery. sFig22. Meta analysis of NOAM before and after phaco-XEN surgery. sFig23. Meta analysis of IOP after surgery between XEN-only and phaco-XEN surgery. sFig24. Meta analysis of IOP after surgery by ethnicity between XEN-only and phaco-XEN surgery. sFig25. Meta analysis of IOP before surgery by ethnicity between XEN-only [file 12886_2022_2502_MOESM4_ESM.pdf]
